# Myosin II regulatory light chain phosphorylation and formin availability modulate cytokinesis upon changes in carbohydrate metabolism

**DOI:** 10.7554/eLife.83285

**Published:** 2023-02-24

**Authors:** Francisco Prieto-Ruiz, Elisa Gómez-Gil, Rebeca Martín-García, Armando Jesús Pérez-Díaz, Jero Vicente-Soler, Alejandro Franco, Teresa Soto, Pilar Pérez, Marisa Madrid, José Cansado

**Affiliations:** 1 Yeast Physiology Group. Department of Genetics and Microbiology. Campus de Excelencia Internacional de Ámbito Regional (CEIR) Campus Mare Nostrum, Universidad de Murcia Murcia Spain; 2 The Francis Crick Institute London United Kingdom; 3 Instituto de Biología Funcional y Genómica (IBFG), Consejo Superior de Investigaciones Científicas, Universidad de Salamanca Salamanca Spain; University of Warwick United Kingdom; Utrecht University Netherlands

**Keywords:** fission yeast, cytokinesis, respiration, myosin II, formin, p21-activated kinase, *Schizosaccharomyces pombe*

## Abstract

Cytokinesis, the separation of daughter cells at the end of mitosis, relies in animal cells on a contractile actomyosin ring (CAR) composed of actin and class II myosins, whose activity is strongly influenced by regulatory light chain (RLC) phosphorylation. However, in simple eukaryotes such as the fission yeast *Schizosaccharomyces pombe*, RLC phosphorylation appears dispensable for regulating CAR dynamics. We found that redundant phosphorylation at Ser35 of the *S. pombe* RLC homolog Rlc1 by the p21-activated kinases Pak1 and Pak2, modulates myosin II Myo2 activity and becomes essential for cytokinesis and cell growth during respiration. Previously, we showed that the stress-activated protein kinase pathway (SAPK) MAPK Sty1 controls fission yeast CAR integrity by downregulating formin For3 levels (Gómez-Gil et al., 2020). Here, we report that the reduced availability of formin For3-nucleated actin filaments for the CAR is the main reason for the required control of myosin II contractile activity by RLC phosphorylation during respiration-induced oxidative stress. Thus, the restoration of For3 levels by antioxidants overrides the control of myosin II function regulated by RLC phosphorylation, allowing cytokinesis and cell proliferation during respiration. Therefore, fine-tuned interplay between myosin II function through Rlc1 phosphorylation and environmentally controlled actin filament availability is critical for a successful cytokinesis in response to a switch to a respiratory carbohydrate metabolism.

## Introduction

Cytokinesis enables the physical separation of daughter cells after mitosis has been completed (Green et al., 2012). In non-muscle animal cells, this process relies on the formation of a contractile actomyosin ring (‘CAR’), composed of actin filaments and myosin II (NMII), which provides the mechanical force for actomyosin contractility (Garrido-Casado et al., 2021; Mangione and Gould, 2019). The prototype NMII is a complex assembled from two heavy chains, two essential light chains (ELC), and two regulatory light chains (RLC), which, in response to phosphorylation, cause NMII to fold into an extended and active conformation (Garrido-Casado et al., 2021). Phosphorylation of RLC at Ser19 is critical for NMII activation and results in the formation of bipolar filaments with increased actin binding affinity and ATPase motor activity (Garrido-Casado et al., 2021; Craig et al., 1983; Trybus and Lowey, 1984). Similar to its deletion or pharmacological inhibition (Bao et al., 2005; Ma et al., 2010), NMII is enzymatically inactive in the absence of RLC phosphorylation at Ser19 (Trybus, 1989), resulting in defective cytokinesis and an increased multinucleation (Komatsu et al., 2000). Several kinases are involved in RLC phosphorylation at Ser19 and NMII activation during cleavage furrow accumulation and CAR contraction during cytokinesis (Garrido-Casado et al., 2021). RLC phosphorylation at sites other than Ser19 provides additional layers of regulation for the positive or negative modulation of NMII contractile activity within specific cellular contexts (Garrido-Casado et al., 2021).

*S. pombe* is a Crabtree-positive fission yeast that grows through either fermentative or respiratory metabolism and is a well-established model organism for the study of cytokinesis (Pollard and Wu, 2010; Rincon and Paoletti, 2016; Balasubramanian et al., 2004). This simple eukaryote uses a CAR with two myosin-II heavy chains, Myo2 and Myp2/Myo3, to divide (Wang et al., 2020). Myo2 is essential for viability and cytokinesis during unperturbed growth, whereas Myp2 plays a non-essential but important role during CAR constriction, and in response to salt stress (Laplante et al., 2015; Palani et al., 2017; Okada et al., 2019). In contrast to NMII, Myo2 does not form filaments at physiological saline concentrations, but instead adopts a unipolar organization with head domains exposed to the cytoplasm and tails anchored in medial precursor nodes of the CAR at mitotic onset (Pollard et al., 2017; Laporte et al., 2011; McDonald et al., 2017; Laplante et al., 2016). The essential formin Cdc12 nucleates and elongates actin filaments at the nodes, whereas Myo2 promotes the fusion of the equatorial nodes to form a mature CAR (Chang et al., 1997; Kovar et al., 2003; Vavylonis et al., 2008). For3, a non-essential diaphanous-like formin that assembles actin cables for cellular transport, also plays an important role in nucleating actin filaments for the CAR during cytokinesis (Coffman et al., 2013; Gómez-Gil et al., 2020). In response to environmental cues and cytoskeletal damage, Sty1, a p38 MAPK ortholog and core effector of the SAPK pathway, blocks cell division by reducing For3 levels and the availability of actin filaments for the CAR (Gómez-Gil et al., 2020).

Cdc4 and Rlc1 are the respective fission yeast ELC and RLC shared by Myo2 and Myp2 (Le Goff et al., 2000; Naqvi et al., 2000; McCollum et al., 1995). Early evidence indicated that the p21/Cdc42-activated kinase (PAK) ortholog Pak1/Shk1/Orb2 phosphorylates Ser35 and Ser36 of Rlc1, which are homologous to RLC Thr18 and Ser19 in NMIIs (Loo and Balasubramanian, 2008). Phosphorylation of Rlc1 at Ser35 and Ser36 by Pak1 delayed cytokinesis, whereas expression of a non-phosphorylatable mutant (*rlc1-S35A S36A*), resulted in premature CAR constriction (Loo and Balasubramanian, 2008). This is consistent with in vitro data showing that Rlc1 phosphorylation reduces the interaction of Myo2 with actin during force generation (Pollard et al., 2017). This and subsequent work identified Ser35 as the sole target of Pak1 both in vitro and in vivo (Pollard et al., 2017; Magliozzi et al., 2020). However, another study described that the average in vitro motility rate of purified Myo2 bound to the Rlc1-*S35A S36A* mutant is reduced by ∼25% compared to that of the myosin bound to wild-type Rlc1, and that phosphorylation at both sites is positive for CAR constriction dynamics (Sladewski et al., 2009). While the essential role of RLC phosphorylation for NMII activity is well established in animal cells, the biological significance of Rlc1 phosphorylation at Ser35 during cytokinesis remains unclear.

Here, we show that modulation of Myo2 activity by Rlc1 phosphorylation at Ser35 is essential for fission yeast cytokinesis and proliferation during respiratory growth. This modification is exerted by Pak1 together with Pak2, a second PAK ortholog whose expression increases during respiration. Rlc1 phosphorylation at Ser35 becomes essential due to the reduced availability of For3-nucleated actin filaments caused by SAPK activation during respiration-induced oxidative stress. Thus, formin-dependent actin filament nucleation and myosin II activity are coupled for optimal control of cytokinesis in response to changes in MAPK signaling and carbon source metabolism.

## Results

### Myosin II regulatory light chain phosphorylation is essential for *S. pombe* cytokinesis and growth during respiration

**Figure 1.**
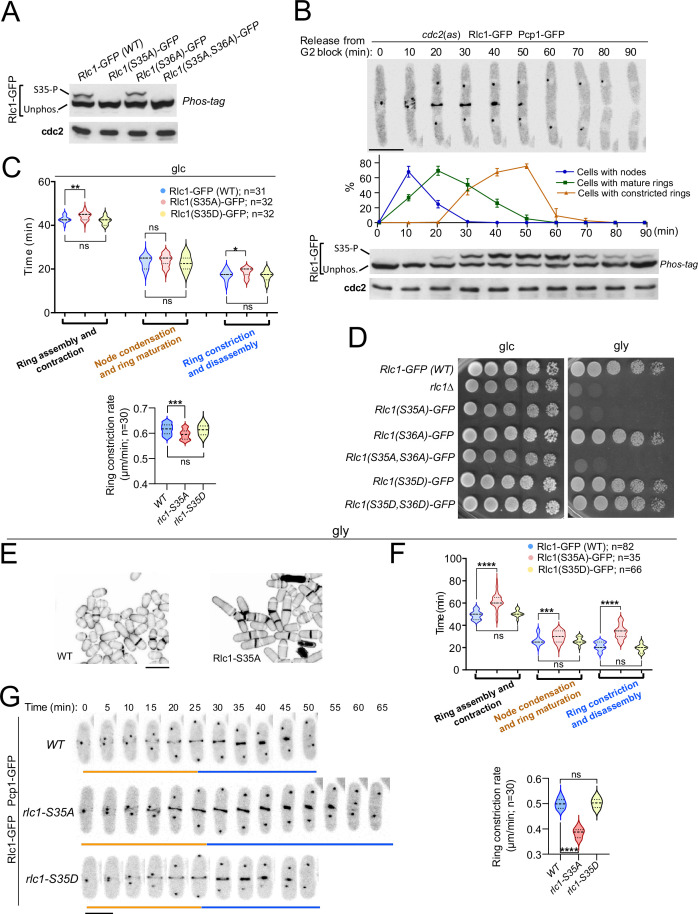
Myosin-II regulatory light chain phosphorylation is essential for *S*. *pombe* cytokinesis and growth during respiration. (**A**) Total protein extracts from the indicated strains grown exponentially in YES-glucose medium were resolved by Phos-tag SDS-PAGE, and the Rlc1-GFP fusion was detected by incubation with anti-GFP antibody. Anti-Cdc2 was used as a loading control. Rlc1 isoforms, phosphorylated (**S35–P**), and non-phosphorylated at Ser35 (Unphos), are indicated. The blot corresponds to a representative experiment that was repeated at least three times and the trend of the mobility shift was reproducible. (**B**) Cells with a *cdc2-asM17* analog-sensitive mutant allele expressing a Rlc1-GFP genomic fusion were arrested at G2 in YES-glucose medium supplemented with 3-NM-PP1 and incubated in the same medium without the kinase analog for the indicated times. Upper panels: time-lapse images of a representative cell showing Rlc1-GFP localization and mitotic progression monitored by Pcp1-GFP-labeled SPBs (scale bar: 10 µm). The ratios of cells with nodes, mature rings, and constricted rings over time after release from the G2 arrest (as mean ± SD from three different experiments) are shown. Lower panels: Western blot analysis of Rlc1-GFP mobility by Phos-tag SDS-PAGE after release from the G2 block. The image corresponds to a representative experiment that was repeated at least three times with similar results. (**C**) Upper: times for ring assembly and contraction, node condensation/ring maturation, and ring constriction and disassembly were estimated for the indicated strains growing exponentially in YES-glucose (glc) medium, by time-lapse confocal fluorescence microscopy. Mitotic progression was monitored using Pcp1-GFP-labeled SPBs. Lower: ring constriction rates (μm/min), were determined for the indicated strains. *n* is the total number of cells scored from three independent experiments, and data are presented as violin plots. Statistical comparison between the two groups was performed by unpaired Student’s *t*-test. ***, p<0.005; **, p<0.005; *, p<0.05; ns, not significant. (**D**) Decimal dilutions of strains of the indicated genotypes were spotted on solid plates with YES-glucose (glc), or YES-glycerol (gly), incubated at 30 °C for 3 (glc) or 5 days (gly), and photographed. The image corresponds to a representative experiment that was repeated at least three times with similar results. (**E**) Representative maximum projection confocal images of cells grown in YES-glycerol for 12 hr after cell-wall staining with calcofluor white. Scale bar: 10 µm (**F**) Upper: times for total ring assembly and contraction, node condensation/ring maturation, and ring constriction were estimated cells of the indicated strains grown exponentially in YES-glycerol medium by time-lapse fluorescence confocal microscopy. Lower: ring constriction rates (μm/min), were determined for the indicated strains. *n* is the total number of cells, and data are presented as violin plots. Statistical comparison between the two groups was performed by unpaired Student’s *t*-test. ****, p<0.0001; ***, p<0.0005; ns, not significant. (**G**) Representative maximum-projection time-lapse images of Rlc1 dynamics at the equatorial region of cells growing in YES-glycerol. Mitotic progression was monitored using Pcp1-GFP-labeled SPBs. Time interval is 5 min. Scale bar: 10 µm. Figure 1—source data 1.Source data for Figure 1. Figure 1—source data 2.Western blot images for Figure 1A and B.

To gain further insight into the contribution of RLC phosphorylation to the myosin II-dependent control of cytokinesis in *S. pombe*, we expressed a C-terminal GFP-tagged version of Rlc1 under the control of its native promoter in *rlc1∆* cells. This construct was fully functional and suppressed the defective CAR positioning and multiseptation associated with the lack of Rlc1 function (Figure 1—figure supplement 1A; Le Goff et al., 2000). The Rlc1-GFP fusion migrates as two distinct bands by Phos-tag SDS-PAGE analysis in extracts from exponentially growing cells (Figure 1A). The Rlc1 mobility of a mutant in which Ser36 was changed to alanine (Rlc1(S36A)-GFP) was similar to that of the wild-type. In contrast, only the faster-migrating band was present in mutants expressing Rlc1(S35A)-GFP or Rlc1(S35A S36A)-GFP fusions (Figure 1A), confirming that the slower-migrating band corresponds to Rlc1 phosphorylated at Ser35.

**Figure 1—figure supplement 1.**
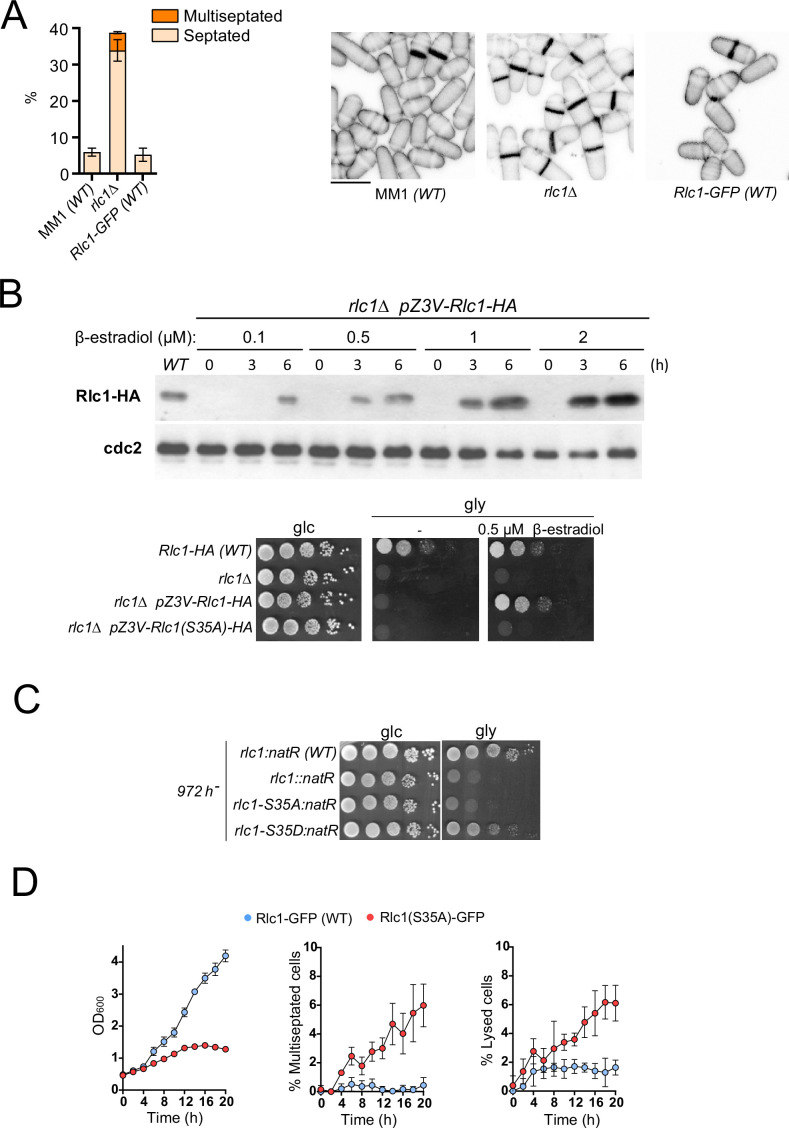
Rlc1 phosphorylation at Ser35 is essential for *S*. *pombe* respiratory growth. (**A**) Left: Strains of the indicated genotypes were grown in YES-glucose liquid medium for 24 hr, and the percentage of septated and multiseptated cells were quantified. Data correspond to three independent experiments and are presented as mean ± SD. Right: representative maximum projection confocal images of cells from the indicated strains after cell-wall staining with calcofluor white. Scale bar: 10 µm. (**B**) Upper: the strain rlc1Δ Z3EVpr:Rlc1-HA was grown in YES-glucose medium to mid-log phase, and the culture was then treated with either 0.1, 0.5. 1, or 2 µM β-estradiol for 0, 3, and 6 hr. Total extracts were resolved by SDS-PAGE, and Rlc1 levels were detected by incubation with anti-HA antibody. Anti-Cdc2 was used as a loading control. The Western blot image corresponds to a representative experiment that was repeated at least three times with similar results. Lower: decimal dilutions of strains of the indicated genotypes were spotted on plates with YES-glucose (glc) or YES-glycerol (gly), with or without 0.5 µM β-estradiol, incubated at 30°C for three days, and photographed. (**C**) Decimal dilutions of strains of the indicated genotypes were spotted on plates with YES-glucose (glc) or YES-glycerol (gly), and photographed. (**D**) The indicated strains were grown in YES-glucose to a final OD_600_ = 0.2 and shifted to YES-glycerol and incubated at 28°C. The OD_600_ value and the percentage of multiseptated and lysed cells were determined at the indicated times. Data correspond to three independent experiments and are presented as mean ± SD. Figure 1—figure supplement 1—source data 1.Source data for Figure 1—figure supplement 1. Figure 1—figure supplement 1—source data 2.Western blot images for Figure 1B.

**Figure 1—figure supplement 2.**
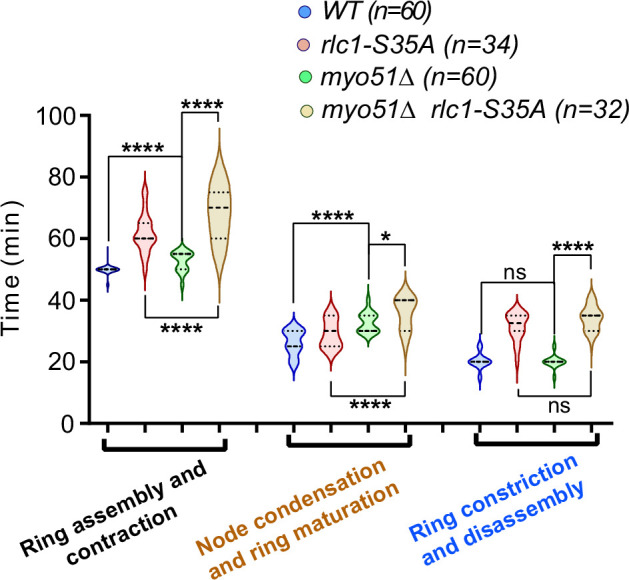
Myo51 and Ser35-phosphorylated Rlc1 collaborate for contractile actomyosin ring (CAR) assembly during respiration. Times for ring assembly and contraction, node condensation/ring maturation, and ring constriction and disassembly were estimated for the indicated strains growing exponentially in YES-glucose medium (glc), by time-lapse confocal fluorescence microscopy. Mitotic progression was monitored using Pcp1-GFP-labeled SPBs. *n* is the total number of cells scored from three independent experiments, and data are presented as violin plots. Statistical comparison between the two groups was performed by unpaired Student’s *t*-test. ****, p<0.0001; *, p<0.05; ns, not significant. Figure 1—figure supplement 2—source data 1.Source data for Figure 1—figure supplement 2.

To precisely follow the dynamics of Rlc1 phosphorylation and localization during the cell cycle, we expressed the Rlc1-GFP version in cells carrying an analog-sensitive version of the Cdk1 kinase ortholog Cdc2 (*cdc2-asM17*) (Aoi et al., 2014) and a Pcp1-GFP fusion (pericentrin SPB component; internal control for mitotic progression). Simultaneous live fluorescence microscopy and Phos-tag SDS-PAGE analysis of synchronized cells released from the G2 arrest showed that in vivo Rlc1 phosphorylation at Ser35 was very low at nodes during CAR assembly, gradually increased during ring maturation, peaked at the onset of CAR contraction until the final stages, and slowly decreased slowly during septum closure and cell separation (Figure 1B). As previously suggested (Loo and Balasubramanian, 2008), these results confirm that in vivo Rlc1 phosphorylation at Ser35 is enhanced during CAR constriction and septum formation.

Time-lapse fluorescence microscopy of asynchronous glucose-growing cells revealed a minimal but statistically significant increase in the total time for ring constriction and disassembly in Rlc1(S35A)-GFP cells as compared to wild-type cells (18.36±2.06 *vs* 17.26±2.17 min, respectively), with a slightly slower ring constriction rate (0.595±0.02 *vs* 0.616+0.02 μm/min, respectively) (Figure 1C). Cells expressing a dual phospho-mimicking form of Rlc1 (Rlc1-S35D S36D) exhibit normal CAR dynamics and support cytokinesis like wild-type cells (Sladewski et al., 2009). Similarly, CAR dynamics and ring constriction rates were identical between wild-type and Rlc1(S35D)-GFP cells (Figure 1C). In contrast to non-muscle animal cells, where phosphorylation of the regulatory light chain is essential for NMII activity (Garrido-Casado et al., 2021), in vivo Rlc1 phosphorylation during *S. pombe* growth in the presence of glucose only minimally affects myosin II function for CAR dynamics.

These findings prompted us to search for other environmental and/or nutritional condition/s where Rlc1 phosphorylation-dependent control of myosin II activity might become essential for fission yeast cytokinesis. A recent study described that *rlc1∆* cells struggle to grow in a glycerol-based medium, which imposes a respiratory metabolism (Malecki et al., 2016), as *rlc1∆* cell growth was strongly reduced in plates containing 3% glycerol plus 0.08% glucose (Figure 1D). Strikingly, the unphosphorylated mutants *rlc1-S35A* and *rlc1-S35A S36A*, but not *rlc1-S36A* or the phosphomimetic versions *rlc1-S35D* and *rlc1-S35D S36D*, also grew very slowly in this medium (Figure 1D). This phenotype was dependent on Rlc1 phosphorylation at Ser35, as a conditional expression of an Rlc1-HA fusion in *rlc1∆* cells by the β-estradiol-regulated promoter (Ohira et al., 2017) allowed their growth on glycerol, whereas conditional expression of the unphosphorylated Rlc1(S35A)-HA mutant did not (Figure 1—figure supplement 1B). *rlc1∆* and *rlc1-S35A* cells on a prototrophic 972 hr background, but not those expressing the *rlc1-S35D* allele, were also growth defective on glycerol (Figure 1—figure supplement 1C), confirming that nutritional auxotrophic markers in the strains are not responsible for this phenotype.

In contrast to wild-type Rlc1, the growth of *rlc1-S35A* cells transferred to a glycerol-based liquid medium was limited to 3–4 additional divisions (Figure 1—figure supplement 1D), with a progressive increase in multiseptated cells with thickened septa and lysed cells, indicating the presence of a cytokinetic defect (Figure 1E, Figure 1—figure supplement 1D). Accordingly, the total ring assembly and contraction time in glycerol-incubated *rlc1-S35A* cells were much longer than in wild-type cells (61.67±7.36 *vs* 49.27±3.69 min, respectively) (Figure 1F–G). The cytokinetic delay was most pronounced during ring constriction and disassembly (34.00±6.16 *vs* 21.40±3.62 min, respectively), with a marked reduction in the ring constriction rate (0.384±0.02 *vs* 0.500+0.02 μm/min, respectively) (Figure 1F–G). Conversely, expression of the phosphomimetic *rlc1-S35D* allele did not alter CAR dynamics and constriction rate when grown with glycerol (Figure 1F–G).

Myo51 is a type V myosin that plays an important role in *S. pombe* during ring assembly, as *myo51∆* cells complete this process later than normal (Figure 1—figure supplement 2; Laplante et al., 2015). Myo51 deletion further and specifically increased the CAR assembly time in *rlc1-S35A* cells during glycerol growth (Figure 1—figure supplement 2), suggesting that Myo51 cooperates with Myo2 in this process. Thus, in vivo Rlc1 phosphorylation at Ser35 is essential for the modulation of *S. pombe* cytokinesis and cell division during respiratory growth.

### p21-activated kinases Pak2 and Pak1 phosphorylate Rlc1 at Ser35 to positively control fission yeast cytokinesis during respiration

**Figure 2.**
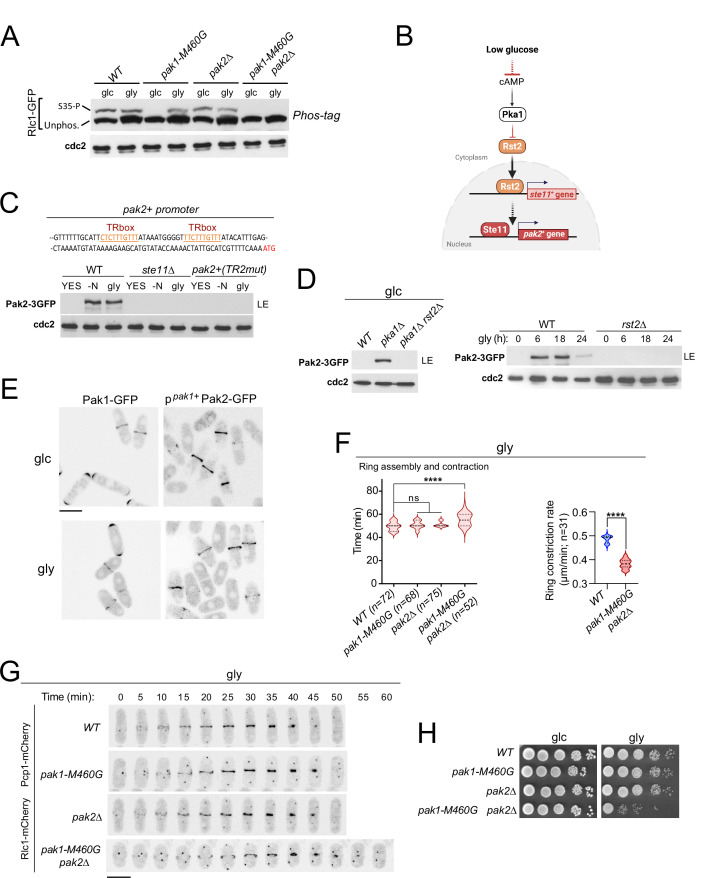
p21/Cdc42-activated kinase Pak2 phosphorylates Rlc1 at Ser35 in concert with Pak1 to positively regulate fission yeast cytokinesis during respiratory growth. (**A**) Total protein extracts from strains of the indicated genotypes growing exponentially in YES-glucose (glc) or YES-glycerol (gly), were resolved by Phos-tag SDS-PAGE, and the Rlc1-GFP fusion was detected by incubation with anti-GFP antibody. Anti-Cdc2 was used as a loading control. Rlc1 isoforms, phosphorylated (**S35–P**) and non-phosphorylated at Ser35 (Unphos), are indicated. The blot corresponds to a representative experiment that was repeated at least three times and the trend of the mobility shift was reproducible. (**B**) Pak2 expression increases specifically during respiratory growth in an Rst2- and Ste11-dependent manner in the absence of cAMP-PKA signaling. See text for further details. (**C**) Upper: Partial nucleotide sequence of the promoter region of the *pak2^+^* gene. The two putative Ste11-binding motifs (TR boxes) are shown in color. Lower: Western blot analysis of Pak2-3GFP levels in wild-type, *ste11∆*, and a mutant strain in which the conserved G in the two putative TR boxes in the *pak2^+^* promoter was replaced by A, grown in YES-glucose, after nitrogen starvation (-N) and in YES-glycerol (gly) for 12 hr. Pak2-3GFP was detected by incubation with anti-GFP antibody, while anti-Cdc2 was used as a loading control. The image corresponds to a representative experiment, which was repeated at least three times with identical results. (**D**) Left: total protein extracts from strains of the indicated genotypes grown exponentially in YES-glucose (left) or in YES-glycerol for the indicated times (right) were resolved by SDS-PAGE, and the Pak2-3GFP fusion was detected by incubation with anti-GFP antibody. Anti-Cdc2 was used as a loading control. Images are representative of experiments repeated at least three times with identical results. (**E**) Representative maximum projection confocal images of exponentially growing cells from Pak1-GFP and p*^pak1+^*-Pak2-GFP cells in YES-glucose (glc) or YES-glycerol (gly). (**F**) Total assembly and contraction times (min) and ring constriction rates (μm/min) were estimated by time-lapse confocal fluorescence microscopy for the indicated strains growing exponentially in YES-glycerol (gly) medium. Mitotic progression was monitored using Pcp1-GFP-labeled SPBs. *n* is the total number of cells scored from three independent experiments, and data are presented as violin plots. Statistical comparison between groups was performed by one-way ANOVA. ****, p<0.0001; ns, not significant. (**G**) Representative maximum-projection time-lapse images of Rlc1 dynamics at the equatorial region in cells growing with YES-glycerol. Mitotic progression was monitored using Pcp1-GFP-labeled SPBs. The time interval is 5 min. Scale bar: 10 µm. (**H**) Decimal dilutions of strains of the indicated genotypes were spotted on plates with YES-glucose or YES-glycerol, incubated at 30 °C for four days, and photographed. The image corresponds to a representative experiment that was repeated at least three times with similar results. Figure 2—source data 1.Source data for Figure 2. Figure 2—source data 2.Western blot images for Figure 2A, C and D.

The essential fission yeast p21 (cdc42/rac)-activated protein kinase (PAK) Pak1/Shk1/Orb2, phosphorylates Rlc1 at Ser35 both in vitro and in vivo (Loo and Balasubramanian, 2008; Magliozzi et al., 2020). Consistently, in vivo phosphorylation of Rlc1-GFP at Ser35 was gradually reduced in glucose-grown cells expressing the analog-sensitive (as) kinase mutant *pak1-M460A* treated with the specific kinase inhibitor 3-BrB-PP1, but not in the presence of the solvent control (Figure 2—figure supplement 1A). Interestingly, light chain phosphorylation at Ser35 was absent in glucose-growing cells expressing the hypomorphic mutant allele *pak1-M460G* (Figure 2A; Loo and Balasubramanian, 2008), suggesting that this kinase version is constitutively inactive towards Rlc1. Unexpectedly, Rlc1 remained phosphorylated at Ser35 in *pak1-M460G* cells during glycerol growth (Figure 2A). Rlc1 levels increased by approximately ~1.5-fold during glycerol growth, regardless of the presence of Pak1/2 activity (Figure 2A; Figure 2—figure supplement 1B), although increased Rlc1 expression does not alter fission yeast CAR integrity and/or cytokinesis (Stark et al., 2010). Therefore, other kinase(s) in addition to Pak1 specifically phosphorylate Rlc1 at Ser35 in vivo during respiratory growth.

**Figure 2—figure supplement 1.**
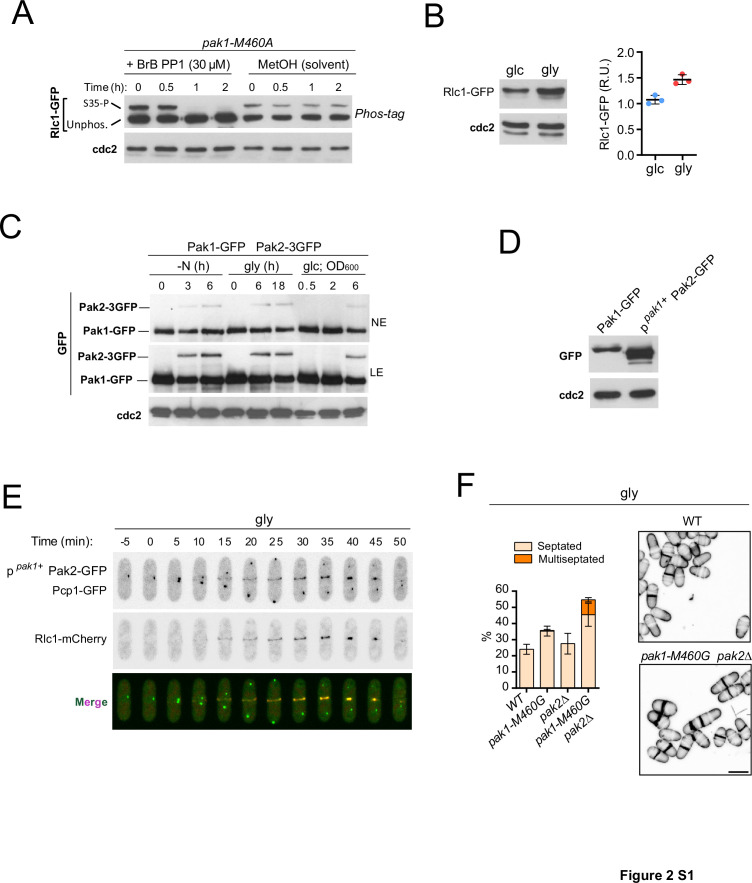
Pak2 regulates cytokinesis during respiration. (**A**) Exponentially growing cells of the analog-sensitive strain *pak1-M460A* growing in YES-glucose were treated with 30 µM of 3-BrB-PP1 for the indicated times, or left untreated in the presence of solvent alone (MetOH). The corresponding protein extracts were resolved by Phos-tag SDS-PAGE, and Rlc1 isoforms were detected by incubation with anti-GFP antibody. Anti-Cdc2 was used as a loading control. S35-P: Rlc1 phosphorylated at Ser35 in vivo. Unphos.: Rlc1 not phosphorylated at Ser35. The blot corresponds to a representative experiment that was repeated at least three times and the trend of the mobility shift was reproducible. (**B**) Left: the Rlc1-GFP fusion was detected in wild-type cells grown in YES-glucose (glc) or YES-glycerol (gly) by Western blot analysis using anti-GFP antibody. Anti-Cdc2 was used as a loading control. Right: Rlc1 levels are expressed as mean relative units ± SD and correspond to experiments performed as biological triplicates. (**C**) Glucose-grown cells of a *S. pombe* strain co-expressing Pak1-GFP and Pak2-3GFP genomic fusions were starved of nitrogen (-N), incubated in YES-glycerol for the indicated times, or incubated in YES-glucose medium until the indicated OD_600_ values were reached. Protein extracts were resolved by SDS-PAGE, and Pak1-GFP and Pak2-3GFP fusions were detected by incubation with anti-GFP antibody. The image corresponds to a representative experiment that was repeated at least three times. NE: bands observed after 5 min film exposure. LE: immunoreactive bands observed after a prolonged exposure of 25 min. (**D**) Total protein extracts from strains expressing either Pak1-GFP or p*^pak1+^*-Pak2-GFP fusions and growing exponentially in YES-glucose were resolved by SDS-PAGE. Fusions were detected by incubation with an anti-GFP antibody. The image corresponds to a representative experiment, which was repeated at least three times with similar results. (**E**) Representative maximum-projection time-lapse images of Pak2 and Rlc1 dynamics at the CAR in cells co-expressing p*^pak1+^*-Pak2-GFP and Rlc1-mCherry genomic fusions and grown in YES-glycerol (gly). Mitotic progression was monitored using Pcp1-GFP-labeled SPBs. Time interval is 5 min. (**F**) Left: Strains were grown in YES-glycerol for 12 hr, and the percentage of septated and multiseptated cells was quantified. Data correspond to three independent experiments and are presented as mean ± SD. Right: representative maximum projection confocal images of cells from the indicated strains after cell-wall staining with calcofluor white. Figure 2—figure supplement 1—source data 1.Source data for Figure 2—figure supplement 1. Figure 2—figure supplement 1—source data 2.Western blot images for Figure 1A, B, C and D.

A reasonable candidate for this role is Pak2, a PAK homolog whose overexpression restores the viability and normal morphology of fission yeast cells in the absence of Pak1 function (Sells et al., 1998; Yang et al., 1998). Indeed, Rlc1 phosphorylation at Ser35 was absent during respiratory growth in a *pak1-M460G pak2∆* double mutant, but remained in *pak2∆* cells grown on either glucose or glycerol (Figure 2A). Pak2 was not detected in a glucose-grown strain co-expressing Pak1-GFP and Pak2-3GFP genomic fusions, but its expression level increased in the absence of nitrogen, in the presence of glycerol, or during the stationary phase in a glucose-rich medium (Figure 2—figure supplement 1C). Pak2 expression was very low under these conditions, and could only be detected after long exposure times of the immunoblots (>20 min; LE; Figure 2—figure supplement 1C). In *S. pombe*, *pak2^+^* mRNA levels increase during nitrogen starvation through a mechanism dependent on Ste11, a transcription factor that activates gene expression during the early steps of the sexual differentiation ( +) (Mata and Bähler, 2006).

The *pak2^+^* promoter contains two consecutive copies of a putative Ste11-binding motif known as the TR box (consensus sequence 5 +-TTCTTTGTTY-3') (Figure 2C; Sugimoto et al., 1991). Induced expression of Pak2-3GFP during nitrogen starvation or glycerol growth was abolished in *ste11∆* cells, and in a strain in which *pak2^+^* + is controlled by its endogenous promoter mutated at both TR boxes (Figure 2C). The Zn-finger transcriptional factor Rst2, whose activity is negatively regulated by the cAMP-PKA signaling pathway in the presence of glucose, positively regulates *ste11^+^* expression during nitrogen or glucose starvation ( +) (Kunitomo et al., 2000). Rst2 deletion suppressed the constitutive expression of Pak2 in glucose-grown *pka1∆* cells and in the presence of glycerol (Figure 2D). Thus, Pak2 expression is constitutively repressed by glucose cAMP-PKA signaling and increases specifically during respiration in an Rst2- and Ste11-dependent manner.

The very low expression levels of the Pak2-3GFP genomic fusion prevented its microscopic visualization during nutrient starvation. To circumvent this situation, we obtained a strain expressing a Pak2-GFP fusion under the control of the native *pak1^+^* promoter (p*^pak1+^*-Pak2-GFP). The relative expression levels of p*^pak1+^*-Pak2-GFP were approximately +–3-fold higher than those of the Pak1-GFP genomic fusion (Figure 2—figure supplement 1D). However, in contrast to Pak1-GFP, which is targeted to the cell poles and the CAR during vegetative growth with either glucose or glycerol, the p*^pak1+^*-Pak2-GFP fusion localized exclusively to the CAR under both conditions (Figure 2E). Pak2 co-localized with Rlc1 throughout the cytokinetic process in glycerol-grown cells, from the early steps of CAR assembly and maturation to the later stages of ring constriction (Figure 2—figure supplement 1E).

Compared to wild-type cells, *pak1-M460G* and *pak2∆* cells showed no defects in cytokinesis, septation, or growth during respiration (Figure 2F–H; Figure 2—figure supplement 1F). Strikingly, the mean times for CAR assembly and constriction, as well as ring constriction rates, were longer in *pak1-M460G pak2∆* double mutant cells (Figure 2F and G). *pak1-M460G pak2∆* cells were also multiseptated (Figure 2—figure supplement 1F) and displayed a growth defect in this carbon source (Figure 2H). Our observations support that Pak1 is entirely responsible for the in vivo Rlc1 phosphorylation at Ser35 during fermentation, whereas Pak2, whose expression is induced upon nutrient starvation, collaborates with Pak1 to phosphorylate Rlc1 at this residue to regulate cytokinesis during respiratory growth.

### Rlc1 phosphorylation is critical for cytokinesis during respiration due to reduced actin cable nucleation imposed by SAPK activation

For3 assembles actin cables for cellular transport and cooperates with the essential formin Cdc12 to nucleate actin filaments for the CAR during cytokinesis (Coffman et al., 2013; Gómez-Gil et al., 2020). CAR assembly and contraction time increased as the rate of ring constriction was significantly reduced in *for3∆* cells grown with glycerol (Figure 3—figure supplement 1A–B). This led to an accumulation of multiseptated and lysed cells and a marked growth defect (Figure 3—figure supplement 1C–E). Thus, For3-mediated actin cable nucleation is crucial for proper cytokinesis and growth of *S. pombe* during respiration.

**Figure 3.**
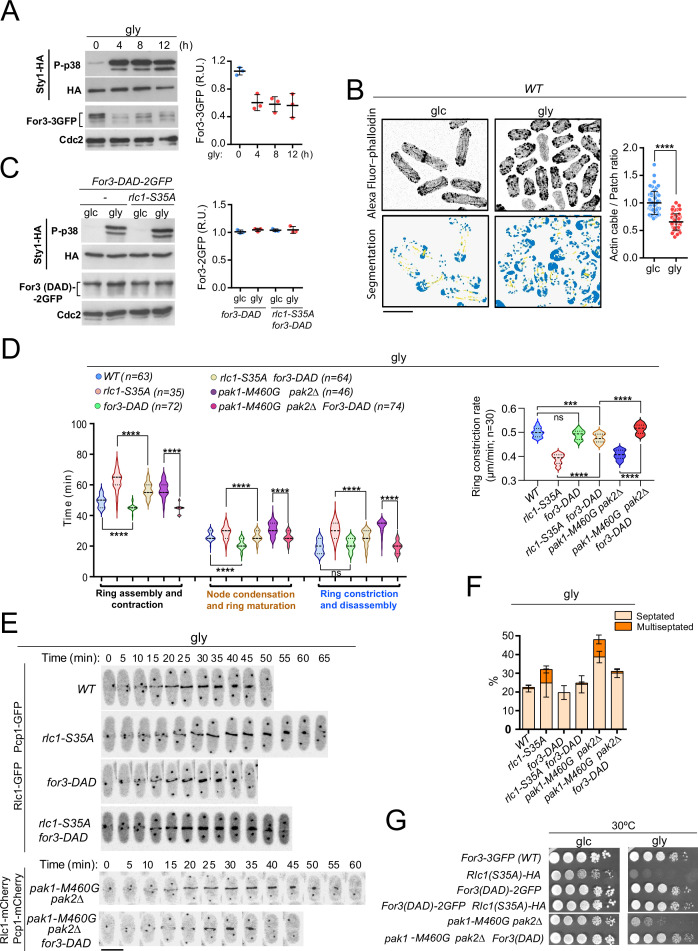
PAK phosphorylation of Rlc1 is critical for *S*. *pombe* cytokinesis during respiration due to impaired For3-dependent actin cable nucleation imposed by SAPK activation. (**A**) Left: *S. pombe* cells expressing genomic Sty1-HA and For3-3GFP fusions were transferred to YES-glycerol medium (gly) for the indicated times. Activated/total Sty1 was detected using anti-phospho-p38 and anti-HA antibodies, respectively. Total For3 was detected with anti-GFP antibody. Anti-Cdc2 was used as a loading control. Right: For3 expression levels are expressed as mean relative units ± SD and correspond to experiments performed as biological triplicates. (**B**) Representative maximum projection images of Alexa Fluor phalloidin-stained *S. pombe* cells grown in YES-glucose medium (glc) or in YES-glycerol (gly) for 12 hr. Segmentation analysis using the Ilastik routine is shown below each image. Quantification data correspond to actin cable to patch ratio of G2 cells (n=51) and are presented as mean relative units ± SD. ****, p<0.0001, as calculated by unpaired Student’s *t*-test. Scale bar: 10 µm. (**C**) Left: *S. pombe* wild-type and *rlc1-S35A* strains expressing genomic Sty1-HA and For3 (DAD)–2GFP fusions were grown in YES-glucose (glc) until mid-log phase and transferred to YES-glycerol (gly) medium for 12 hr. Activated/total Sty1 was detected using anti-phospho-p38 and anti-HA antibodies, respectively. Total For3 was detected with anti-GFP antibody. Anti-Cdc2 was used as a loading control. Right: For3 expression levels are expressed as mean relative units ± SD and correspond to experiments performed as biological triplicates. (**D**) Left: times for ring assembly and contraction, node condensation/ring maturation, and ring constriction and disassembly were estimated for the indicated strains growing exponentially in YES-glycerol medium by time-lapse confocal fluorescence microscopy. Right: ring constriction rates (μm/min), were determined for the indicated strains. *n* is the total number of cells scored from three independent experiments, and data are presented as violin plots. ****, p<0.0001; ***, p<0.001; ns, not significant, as calculated by unpaired Student’s *t*-test. (**E**) Representative maximum-projection time-lapse images of Rlc1 dynamics at the equatorial region in cells from the indicated strains growing in YES-glycerol. Mitotic progression was monitored using Pcp1-GFP-labeled SPBs. Time interval is 5 min. (**F**) The indicated strains were grown in YES-glycerol liquid medium for 12 hr, and the percentage of septated and multiseptated cells was quantified. Data correspond to three independent experiments and are presented as mean ± SD. (**G**) Decimal dilutions of strains of the indicated genotypes were spotted on plates with YES-glucose (glc) or YES-glycerol (gly), incubated at 30 °C or five days, and photographed. Figure 3—source data 1.Source data for Figure 3. Figure 3—source data 2.Western blot images for Figure 3A and C.

Glucose limitation activates Sty1, a p38 MAPK ortholog and the key effector of the SAPK pathway in fission yeast (Madrid et al., 2004). Activated Sty1 down-regulates CAR integrity in *S. pombe* in response to environmental stress by reducing For3 levels (Gómez-Gil et al., 2020). Notably, the transfer of fission yeast cells co-expressing genomic Sty1-HA and For3-3GFP fusions from glucose to glycerol media induced a sustained Sty1 activation and a decrease in For3 protein levels (Figure 3A). Similar to environmental stress (Gómez-Gil et al., 2020), For3 downregulation is associated with increased ubiquitination, as it was attenuated in the proteasome mutant *mts3-1* (Figure 3—figure supplement 1F). The ratio of actin cables to patches was significantly lower in wild-type cells grown in glycerol than in glucose, as revealed by image segmentation analysis of AlexaFluor-488-phalloidin-immunostained cells using the machine learning routine Ilastik (Figure 3B; Berg et al., 2019). Actin patches appeared partially depolarized and their density increased during glycerol growth (Figure 3B). Fluorescence intensity at the cell poles and CAR of activated Cdc42 GTPase (CRIB-3GFP probe), which triggers For3 activation in vivo (Martin et al., 2007), decreased during respiration (Figure 3—figure supplement 2A–B), as did the targeting of a For3-3GFP fusion (Figure 3—figure supplement 2C).

**Figure 3—figure supplement 1.**
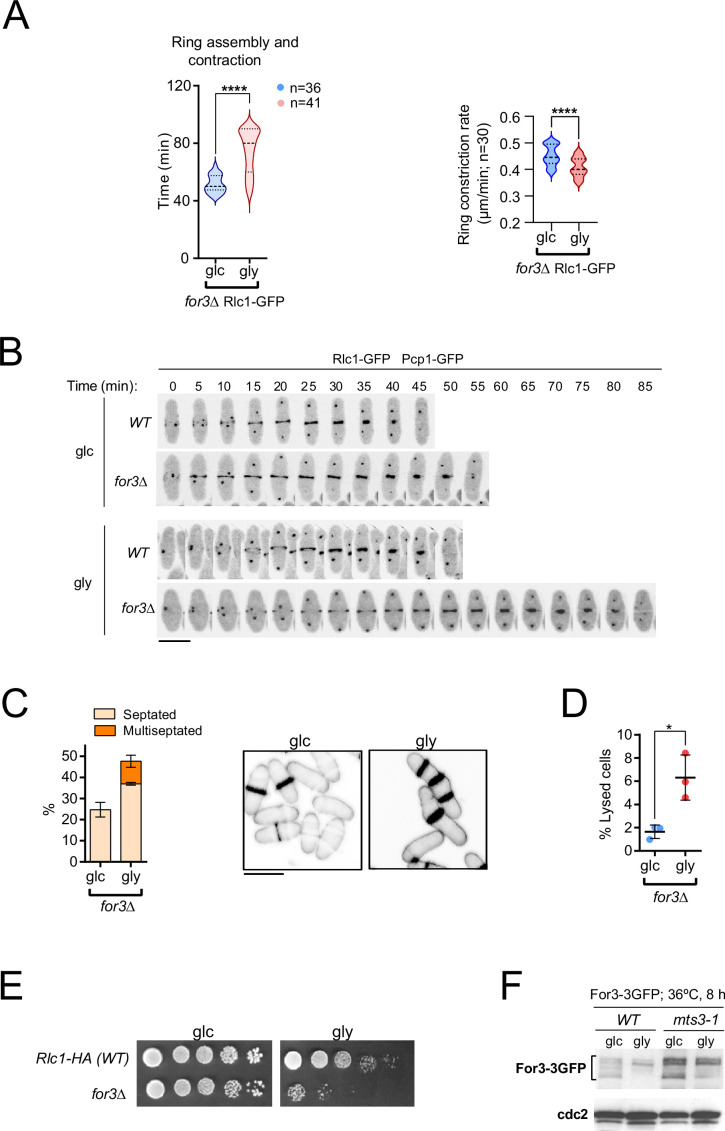
For3 formin is required for *S*. *pombe* cytokinesis during respiratory growth. (**A**) Total ring assembly and contraction times (left) and ring constriction rates (right) (μm/min) were estimated for *for3∆* Rlc1-GFP cells growing exponentially in either YES-glucose (glc) or YES-glycerol (gly) medium by time-lapse confocal fluorescence microscopy. n>30 cells from three independent experiments were scored in each condition, and data are presented as violin plots. ****, p<0.0001, as calculated by unpaired Student’s *t-*test. (**B**) Representative maximum projection time-lapse images of Rlc1 dynamics at the equatorial region in cells from the indicated strains growing in YES-glucose or YES-glycerol. Mitotic progression was monitored using Pcp1-GFP-labeled SPBs. Time interval is 5 min. Scale bar: 10 µm. (**C**) Left: the percentage of septated and multiseptated cells were quantified in *for3∆* cells grown for 12 h in either YES-glucose (glc) or YES-glycerol (gly) medium. Data correspond to three independent experiments and are presented as mean ± SD. Right: representative maximum projection confocal images after cell-wall staining with calcofluor white. Scale bar: 10 µm. (**D**) The percentage of cell lysis was quantified in *for3∆* cells grown for 12 hr in either YES-glucose (glc) or YES-glycerol (gly) medium. Data correspond to three independent experiments and are presented as mean ± SD. *, p<0.05, as calculated by unpaired Student’s *t*-test. (**E**) Decimal dilutions of strains of the indicated genotypes were spotted on plates with YES-glucose (glc) or YES-glycerol (gly), incubated at 30°C for five days, and photographed. The image corresponds to a representative experiment that was repeated at least three times with similar results. (**F**) For3-3GFP levels were determined by Western blot analysis with anti-GFP antibody in extracts from wild-type and the proteasome mutant *mts3-1* grown in YES-glucose (glc) or YES-glycerol (gly) and incubated at 36°C for 8 hr. Anti-Cdc2 was used as a loading control. The image corresponds to a representative experiment that was repeated at least three times with similar results. Figure 3—figure supplement 1—source data 1.Source data for Figure 3—figure supplement 1. Figure 3—figure supplement 1—source data 2.Western blot images for Figure 1F.

**Figure 3—figure supplement 2.**
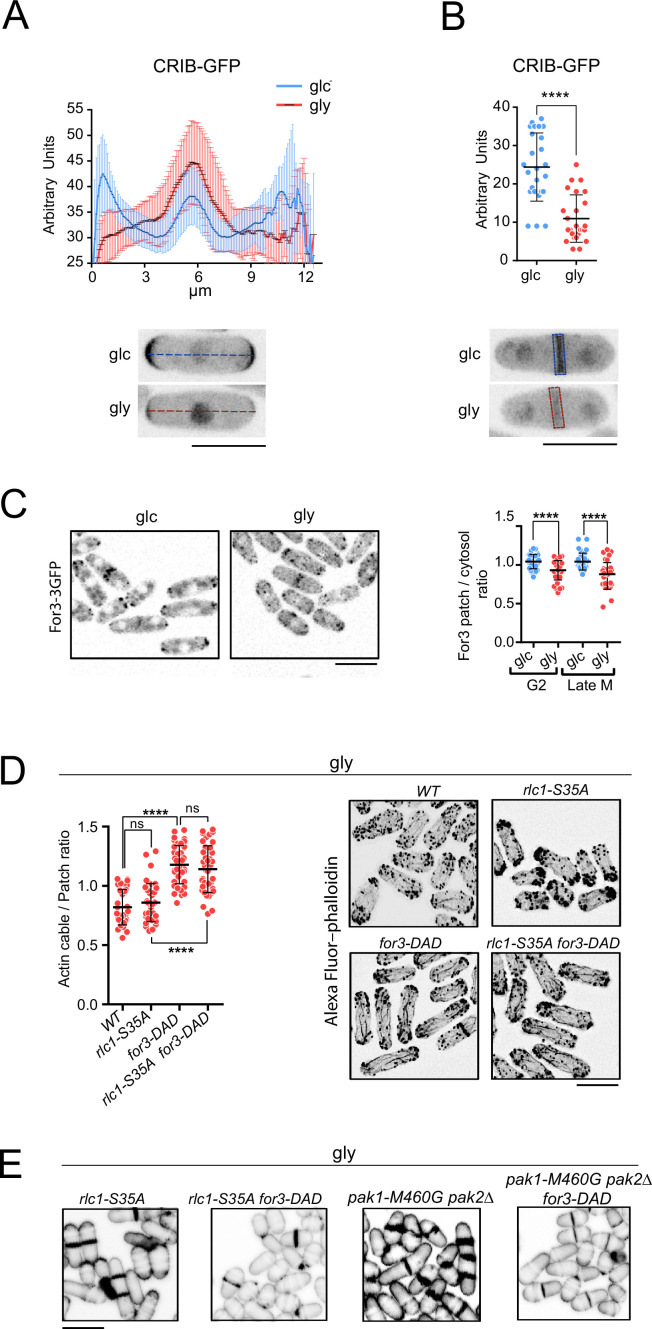
Localization at the cell poles and the contractile actomyosin ring (CAR) of Ccd42 (active) and For3 is reduced during respiratory growth. (**A**) Upper: intensity plots of the CRIB-3GFP fusion (shown as arbitrary fluorescence units), were generated from line scans across the equatorial region of *S. pombe* G2 cells (n=30) grown in YES-glucose (glc) or YES-glycerol (gly). Data are presented as mean ± SD. Lower: representative maximum-projection images of cells grown with glucose and glycerol are shown. Scale bar: 10 µm. (**B**) Upper: the intensity of the CRIB-3GFP fusion at the medial region of dividing cells (n=25) in YES-glucose (glc) or YES-glycerol (gly) was measured and is shown as arbitrary fluorescence units. Data are presented as mean ± SD. ****, p<0.0001, *t*-test. Lower: representative maximum-projection images of dividing cells are shown. Scale bar: 10 µm. (**C**) Left: representative maximum projection images of *S. pombe* cells expressing a For3-3GFP genomic fusion grown in YES-glucose medium or in YES-glycerol for 12 hr. Right: quantification data (mean relative units ± SD) correspond to the For3 dot to cytosol ratio in G2 and late M cells (n=36), grown in glucose or glycerol. ****, p<0.0001, as calculated by unpaired Student’s *t*-test. (**D**) Actin cable to patch ratio of G2 cells from the indicated strains grown in YES-glycerol medium for 12 hr. Quantification data (n=41 cells for each strain) are presented as mean relative units ± SD. ****, p<0.0001; ns, not significant, as calculated by unpaired Student’s *t*-test. (**E**) Representative maximum projection images of Alexa Fluor–phalloidin stained *S. pombe* cells of the indicated strains grown in YES-glycerol medium for 12 hr. Scale bar: 10 µm. (**E**) Representative maximum projection confocal images of cells from the indicated strains after cell-wall staining with calcofluor white. Scale bar: 10 µm. Figure 3—figure supplement 2—source data 1.Source data for Figure 3—figure supplement 2.

In contrast to wild-type cells (Figure 3A), total levels of the constitutively active For3 allele *for3-DAD* fused to GFP, which lacks the intramolecular interaction between the autoregulatory (DAD) and inhibitory (DID) domains to adopt an open and constitutively active conformation (Martin et al., 2007), were not reduced upon Sty1 activation by glycerol (Figure 3C). *for3-DAD* cells displayed thickened actin cables with an increased actin cable-to-patch ratio (Figure 3—figure supplement 2D) and required a shorter time for CAR assembly than the wild-type (Figure 3D–E). Most importantly, *for3-DAD* expression largely suppressed the altered cable organization (Figure 3—figure supplement 2D), the cytokinetic delay and reduced constriction rate (Figure 3D–E), the multiseptated phenotype (Figure 3F; Figure 3—figure supplement 2E), and the defective growth in glycerol of *rlc1-S35A* cells (Figure 3G). *for3-DAD* also rescued the cytokinetic and growth defects in glycerol of *pak1-M460G pak2∆* cells (Figure 3D–G; Figure 3—figure supplement 2E), which lack Rlc1 phosphorylation at Ser35 (Figure 2A). Nevertheless, CAR assembly and contraction took longer in *for3-DAD rlc1-S35A* cells than in *for3-DAD pak1-M460G pak2∆* cells (Figure 3D), suggesting that the regulation of other targets in addition to Rlc1 by Pak1/2 could affect cytokinesis in fission yeast. Thus, For3 and PAK-phosphorylated Rlc1 play a collaborative and biologically significant role during cytokinesis when *S. pombe* grows by respiration.

**Figure 4.**
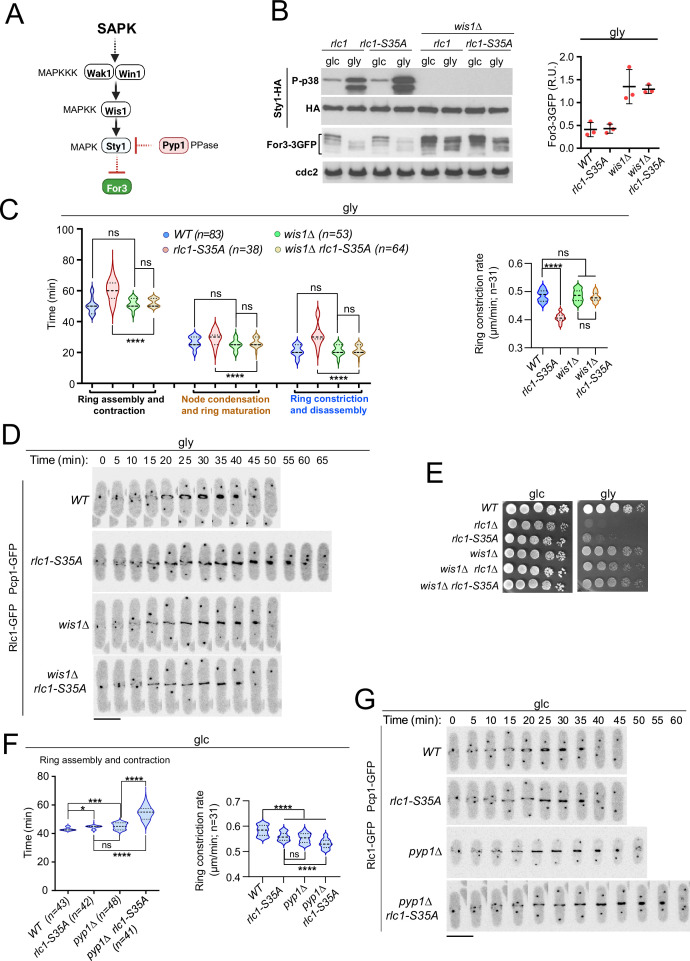
Lack of stress-activated protein kinase (SAPK) signaling restores *S*. *pombe* cytokinesis and growth during respiration in the absence of Rlc1 phosphorylation. (**A**) SAPK signaling triggers For3 downregulation. Please see the text for specific details on the components of the MAPK cascade. (**B**) Left: *S. pombe* strains of the indicated genotypes expressing genomic Sty1-HA and For3-3GFP fusions were grown in either YES-glucose (glc) or YES-glycerol (gly) medium for 12 hr. Activated/total Sty1 were detected with anti-phospho-p38 and anti-HA antibodies, respectively. Total For3 levels were detected with anti-GFP antibody. Anti-Cdc2 was used as a loading control. Right: For3 expression levels in glycerol are presented as mean relative units ± SD and correspond to experiments performed as biological triplicates. (**C**) Left: times for ring assembly and contraction, node condensation/ring maturation, and ring constriction and disassembly were estimated for the indicated strains growing exponentially in YES-glycerol medium by time-lapse confocal fluorescence microscopy. Right: ring constriction rates (μm/min), were determined for the indicated strains. *n* is the total number of cells scored from three independent experiments, and data are presented as violin plots. ****, p<0.0001; ns, not significant, as calculated by unpaired Student’s *t*-test. (**D**) Representative maximum-projection time-lapse images of Rlc1 dynamics at the equatorial region in cells from the indicated strains growing in YES-glycerol. Mitotic progression was monitored using Pcp1-GFP-labeled SPBs. Time interval is 5 min. (**E**) Decimal dilutions of strains of the indicated genotypes were spotted on plates with YES-glucose (glc) or YES-glycerol (gly), incubated at 30 °C or five days, and photographed. The image corresponds to a representative experiment that was repeated at least three times with similar results. (**F**) Total ring assembly and contraction time (left) and ring constriction rates (μm/min) (right) were determined by time-lapse confocal fluorescence microscopy for the indicated strains growing exponentially in YES-glucose medium (glc). *n* is the total number of cells scored from three independent experiments, and data are presented as violin plots. ****, p<0.0001;***, p<0.005; *, p<0.05; ns, not significant, as calculated by unpaired Student’s *t*-test. (**G**) Representative maximum-projection time-lapse images of Rlc1 dynamics at the equatorial region in cells from the indicated strains growing in YES-glucose. Mitotic progression was monitored using Pcp1-GFP-labeled SPBs. Time interval is 5 min. Figure 4—source data 1.Source data for Figure 4. Figure 4—source data 2.Western blot images for Figure 4B.

In agreement with previous observations (Gómez-Gil et al., 2020), For3 levels increased in glucose-grown wild-type or *rlc1-S35A* cells lacking Sty1 activity by deletion of Wis1, the only MAPKK of the SAPK (Pérez and Cansado, 2010), and also in the presence of glycerol (Figure 4A–B). We could not analyze the cytokinetic and growth defects of *rlc1-S35A* cells during respiration in the absence of Sty1, because, unlike mutants lacking Wis1 or Wak1/Win1 (MAPKKs), *sty1∆* and *atf1∆* cells (lacking the Sty1 downstream transcription factor Atf1) are growth defective on this carbon source (Figure 4—figure supplement 1C; Zuin et al., 2008). Nevertheless, similar to the *sty1∆* mutant (Gómez-Gil et al., 2020), *wis1∆* cells exhibited thickened actin cables and an increased actin cable-to-patch ratio during growth on glycerol (Figure 4—figure supplement 1A). Furthermore, Wis1 deletion increased the actin cable- to-patch ratio in *rlc1-S35A* cells (Figure 4—figure supplement 1A), suppressed their delayed cytokinesis and reduced constriction rate (Figure 4C–D) and multiseptation (Figure 4—figure supplement 1B), and restored cell growth in glycerol (Figure 4E).

**Figure 4—figure supplement 1.**
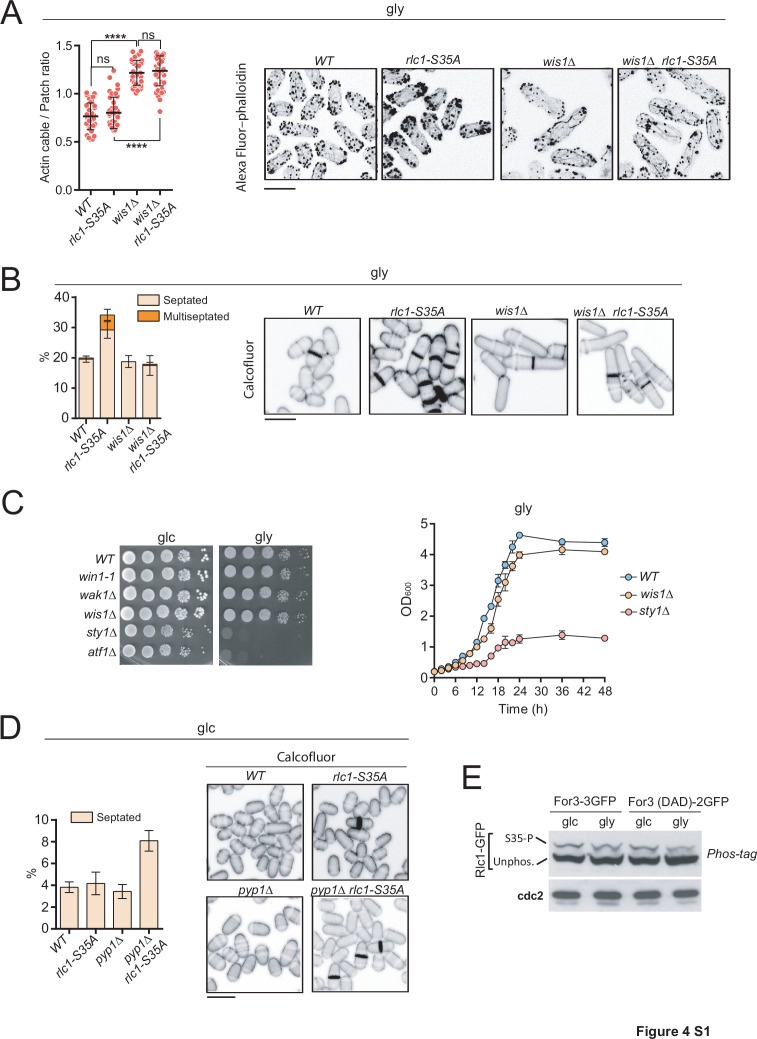
stress-activated protein kinase (SAPK) activation impairs *S*.*pombe* cytokinesis and growth during respiration in the absence of Rlc1 phosphorylation. (**A**) Left: actin cable to patch ratio of G2 cells from the indicated strains grown in YES-glycerol medium for 12 hr. Quantification data (n=41 cells for each strain) are represented as mean relative units ± SD. ****, p<0.0001, *t*-test. Right: representative maximum projection images of Alexa Fluor–phalloidin stained *S. pombe* cells of the indicated strains grown in YES-glycerol medium for 12 hr. Scale bar: 10 µm. (**B**) Left: the indicated strains were grown in liquid YES-glycerol medium for 12 hr, and the percentage of septated and multiseptated cells was quantified. Data correspond to three independent experiments and are presented as mean ± SD. Right: representative maximum projection confocal images of cells from the indicated strains after cell-wall staining with calcofluor white. Scale bar: 10 µm. (**C**) Left: decimal dilutions of strains of the indicated genotypes were spotted on plates with YES-glucose (glc) or YES-glycerol (gly), and photographed. Right: strains were grown in YES-glucose to a final OD_600_ = 0.2, shifted to YES-glycerol, and incubated at 28 °C. The OD_600_ value was determined at the times indicated. Data correspond to three independent experiments and are presented as mean ± SD. (**D**) Left: strains were grown in YES-glucose liquid medium (glc), and the percentage of septated cells was quantified. Data correspond to three independent experiments and are presented as mean ± SD. Right: representative maximum projection confocal images of cells from the indicated strains after cell-wall staining with calcofluor white. (**E**) Total protein extracts from the indicated strains grown exponentially in YES-glucose (glc) or YES-glycerol (gly) were resolved by Phos-tag SDS-PAGE, and the Rlc1-GFP fusion was detected by incubation with anti-GFP antibody. Anti-Cdc2 was used as a loading control. Rlc1 isoforms, phosphorylated (**S35–P**) and non-phosphorylated at Ser35 (Unphos), are indicated. The blot corresponds to a representative experiment that was repeated at least three times and the trend of the mobility shift was reproducible. Figure 4—figure supplement 1—source data 1.Source data for Figure 4—figure supplement 1. Figure 4—figure supplement 1—source data 2.Western blot images for Figure 1E.

Lack of Rlc1 phosphorylation at Ser35 has a limited effect on *S. pombe* CAR dynamics during glucose fermentation (Figure 1C), where basal Sty1 activity is very low (Figure 4B). Our previous data suggest that a constitutive increase in Sty1 activity should be detrimental to cytokinesis in glucose-grown *rlc1-S35A*. Indeed, deletion of the MAPK tyrosine phosphatase Pyp1 (Figure 4A), which increases basal Sty1 activity and reduces For3 levels (Gómez-Gil et al., 2020), further delayed CAR assembly and contraction time andthe constriction rate of glucose-grown *rlc1-S35A* cells (Figure 4F–G), leading to an accumulation of septated cells (Figure 4—figure supplement 1D). Therefore, when For3 levels are reduced by SAPK activation, the tight control of Rlc1 function by phosphorylation at Ser35 becomes essential for *S. pombe* cytokinesis.

SAPK-For3 may act downstream of the PAK-Rlc1 cascade, as the cytokinesis and growth defects of *rlc1-S35A* and *pak1-M460G pak2∆* mutants are suppressed by *for3-DAD*. However, the normal degradation of For3 during respiration in *rlc1-S35A* cells (Figure 4A) excludes this possibility. Moreover, *for3-DAD* expression did not reduce Rlc1 phosphorylation at Ser35 in the presence of glucose or glycerol (Figure 4—figure supplement 1E), suggesting that Pak1/2-Rlc1 is not activated by changes in For3 levels. Thus, both the SAPK-For3 and PAK-Rlc1 pathways act independently and are critical for the successful completion of cytokinesis during respiration.

### Regulation of Myo2 by Rlc1 phosphorylation is essential for *S. pombe* cytokinesis during respiration

**Figure 5.**
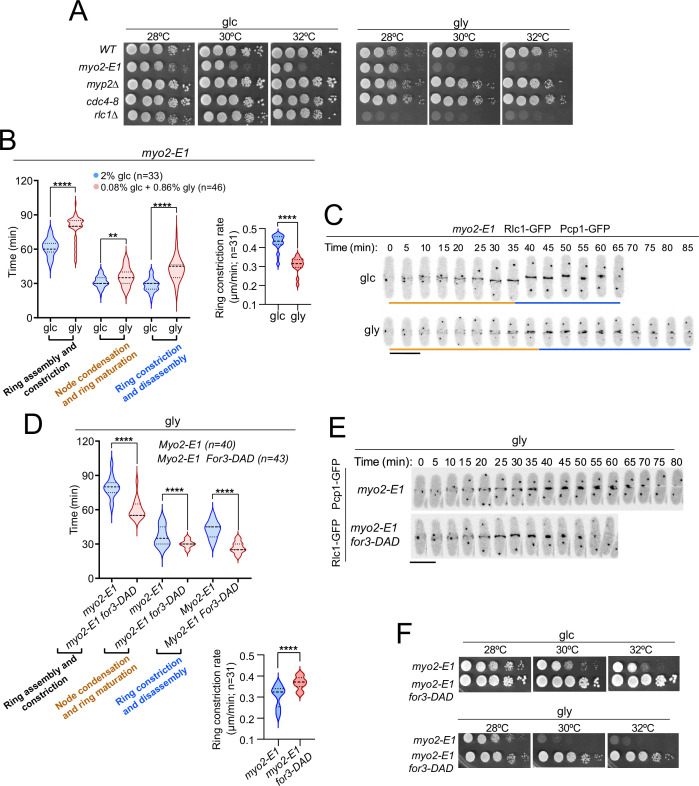
Control of Myo2 activity by Rlc1 phosphorylation regulates *S*. *pombe* cytokinesis and growth during respiration. (**A**) Decimal dilutions of strains of the indicated genotypes were spotted on plates with YES-glucose or YES-glycerol, incubated at 28, 30, and 32 °C for three (glc) or five (gly) days, and photographed. The images correspond to a representative experiment, which was repeated at least three times with similar results. (**B**) Left: times for ring assembly and contraction, node condensation/ring maturation, and ring constriction and disassembly were estimated for *myo2-E1* cells growing in YES-glucose (glc) and YES-glycerol medium (gly) by time-lapse confocal fluorescence microscopy. Mitotic progression was monitored using Pcp1-GFP-labeled SPBs. Right: ring constriction rates (μm/min). *n* is the total number of cells scored from three independent experiments, and data are presented as violin plots. ****, p<0.0001; **, p<0.005, as calculated by unpaired Student’s *t*-test. (**C**) Representative maximum-projection time-lapse images of Rlc1 dynamics at the equatorial region in *myo2-E1* Rlc1-GFP cells growing in YES-glucose (glc) or YES-glycerol (gly). Mitotic progression was monitored using Pcp1-GFP-marked SPBs. Time interval is 5 min. Scale bar: 10 µm. (**D**) Upper: times for ring assembly and contraction, node condensation/ring maturation, and ring constriction and disassembly were estimated for the indicated strains grown on YES-glycerol (gly) using time-lapse confocal fluorescence microscopy. Lower: ring constriction rates (μm/min). *n* is the total number of cells scored from three independent experiments, and data are presented as violin plots. ****, p<0.0001, as calculated by unpaired Student’s *t*-test. (**E**) Representative maximum-projection time-lapse images of Rlc1-GFP dynamics at the equatorial region in *myo2-E1* and *myo2-E1 for3-DAD* cells growing in YES-glycerol. Mitotic progression was monitored using Pcp1-GFP-labeled SPBs. Time interval is 5 min. (**F**) Decimal dilutions of strains of the indicated genotypes were spotted on plates with YES-glucose or YES-glycerol, incubated at 28, 30, and 32°C for three (glc) or five (gly) days, and photographed. The images correspond to a representative experiment that was repeated at least three times with similar results. Figure 5—source data 1.Source data for Figure 5.

The thermosensitive myosin II allele *myo2-E1* shows reduced ATPase activity and actin-filament binding in vitro (Wang et al., 2020). Similar to *rlc1-S35A* cells, *myo2-E1* cells showed a growth defect on glycerol. This was not the case for a hypomorphic mutant in the essential myosin II light chain (*cdc4-8*) or for cells lacking the heavy chain Myp2, which cooperates with Myo2 for cytokinesis (Wang et al., 2020; Okada et al., 2019; Alonso-Matilla et al., 2019; Figure 5A). Accordingly, CAR assembly and constriction times were much longer in *myo2-E1* cells incubated at a semi-restrictive temperature (30 °C), and switched to the permissive temperature in a glycerol-based medium compared to glucose (78.59±11.09 *vs* 61.67±7.56 min, respectively) (Figure 5B–C). The respiration-induced cytokinetic delay was more pronounced during ring constriction/disassembly, with a much slower constriction rate (Figure 5B–C) and the accumulation of multiseptated cells with thickened septa (Figure 5—figure supplement 1A). However, the delay in CAR closure was very similar between *myp2∆* and wild-type cells were grown with glucose or glycerol (~7.5 *vs*~7.7 min) (Figure 5—figure supplement 2A). The *myp2∆* mutant accumulated multiseptated cells in the presence of glycerol, and this phenotype was exacerbated when combined with the *rlc1-S35A* allele (Figure 5—figure supplement 2B).

**Figure 5—figure supplement 1.**
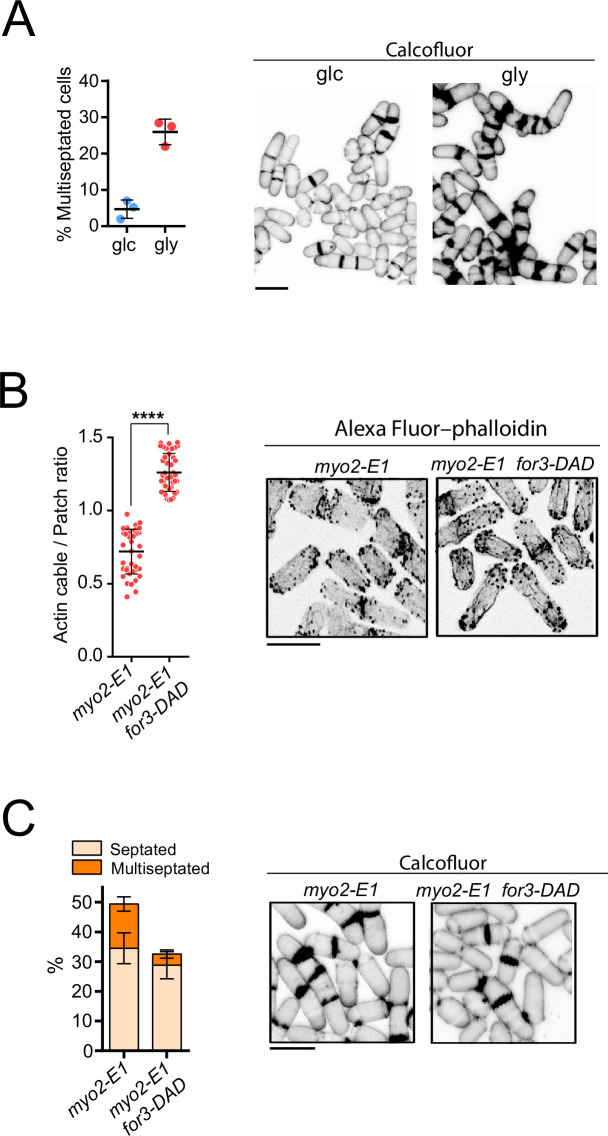
*for3-DAD* expression restores actin filaments and alleviates the septation defects of *myo2-E1* cells during respiration. (**A**) Left: the percentage of multiseptated cells was quantified in *myo2-E1* cells grown exponentially in YES-glucose (glc) or YES-glycerol (gly) for 12 hr. Data correspond to three independent experiments and are presented as mean ± SD. Right: representative maximum projection confocal images of *myo2-E1* cells growing in YES-glucose or YES-glycerol after cell-wall staining with calcofluor white. (**B**) Left: actin cable to patch ratio of G2 cells from the indicated strains growing in YES-glycerol medium for 12 hr. Quantification data (n=40 cells for each strain) are presented as mean relative units ± SD. ****, p<0.0001 *S. pombe* cells of the indicated strains grown in YES-glycerol medium for 12 hr. (**C**) Left: the percentage of septated and multiseptated cells was quantified in *myo2-E1* and myo2-E1 *for3-DAD* cells grown for 12 hr in YES-glycerol medium. Data correspond to three independent experiments and are presented as mean ± SD. Right: representative maximum projection confocal images after cell-wall staining with calcofluor white. Figure 5—figure supplement 1—source data 1.Source data for Figure 5—figure supplement 1.

**Figure 5—figure supplement 2.**
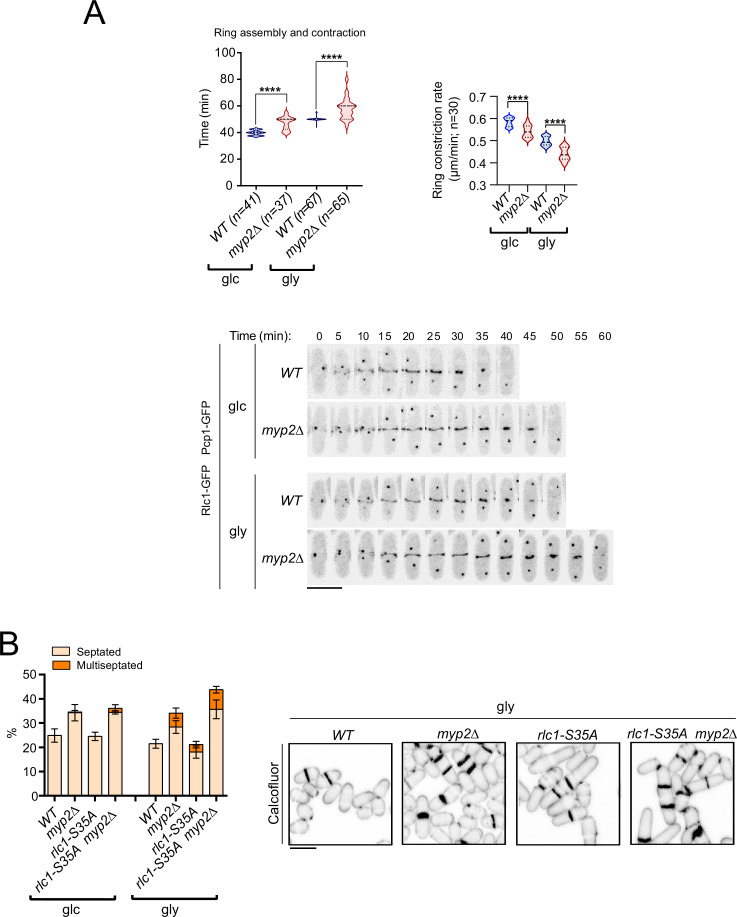
Role of Myp2 on *S*. *pombe* cytokinesis during respiration. (**A**) Upper: ring assembly and contraction times and the ring constriction rates were estimated by time-lapse confocal fluorescence microscopy for wild-type and *myp2∆* cells growing in YES-glucose (glc) or YES-glycerol (gly) medium. Mitotic progression was monitored using Pcp1-GFP-labeled SPBs. *n* is the total number of cells scored from three independent experiments, and data are presented as violin plots. p<0.0001, as calculated by unpaired Student’s *t*-test. Lower: representative maximum-projection time-lapse images of Rlc1 dynamics at the equatorial region of wild-type and *myp2∆* cells growing in YES-glucose (glc) or YES-glycerol (gly). Mitotic progression was monitored using Pcp1-GFP-labeled SPBs. Time interval is 5 min. Scale bar: 10 µm. (**B**) Left: the percentage of septated and multiseptated cells was quantified in the indicated strains grown for 12 hr in YES-glucose (glc) or YES-glycerol (gly). Data correspond to three independent experiments and are presented as mean ± SD. Right: representative maximum projection confocal images of cells grown in YES-glycerol after cell-wall staining with calcofluor white. Scale bar: 10 µm. Figure 5—figure supplement 2—source data 1.Source data for Figure 5—figure supplement 2.

Expression of *for3-DAD* in *myo2-E1* cells restored their altered cable organization (Figure 5—figure supplement 1B), suppressed the cytokinetic delay, the slow rate of ring constriction, and the multiseptated phenotype (Figure 5D–E; Figure 5—figure supplement 1C), and defective growth with glycerol at semi-restrictive temperatures (Figure 5F). Therefore, regulation of Myo2 function by in vivo phosphorylated Rlc1 is critical for *S. pombe* cytokinesis and division during respiration due to the reduced actin filament nucleation imposed by SAPK activation.

### Exogenous antioxidants bypass the need to regulate Myo2 by Rlc1 phosphorylation during respiratory growth cytokinesis

**Figure 6.**
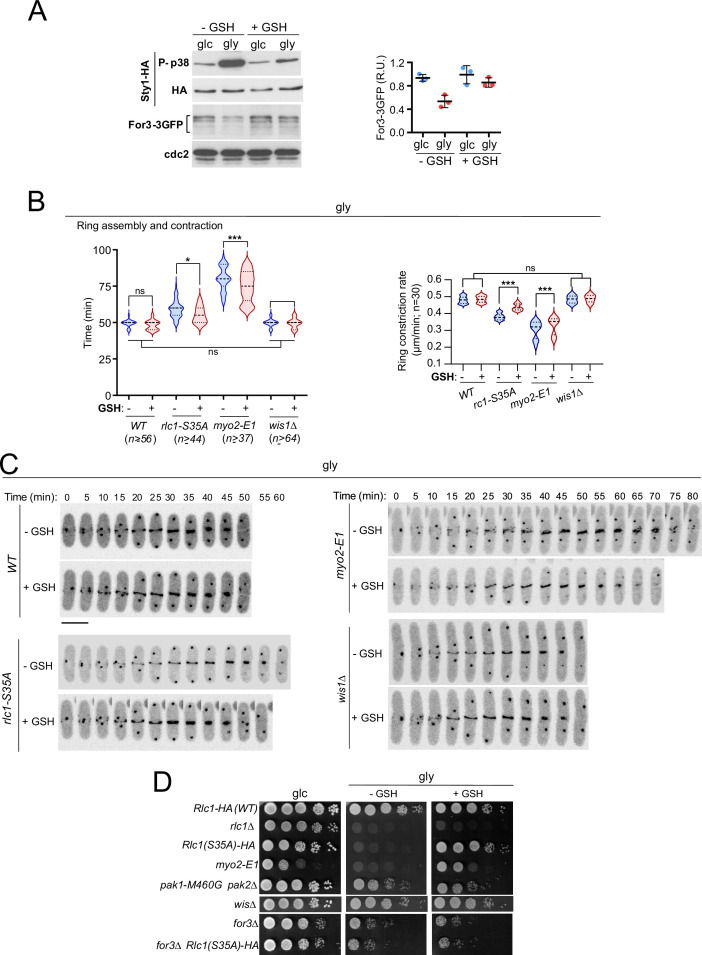
Exogenous antioxidants bypass the need for Rlc1 phosphorylation to regulate myosin II activity and cytokinesis during respiratory growth. (**A**) Left: *S. pombe* wild-type cells expressing genomic Sty1-HA and For3-3GFP fusions were grown to mid-log phase in YES-glucose (glc) or YES-glycerol (gly), with or without 0.16 mM reduced glutathione (GSH). Activated/total Sty1 was detected with anti-phospho-p38 and anti-HA antibodies, respectively. Total For3 levels were detected with anti-GFP antibody. Anti-Cdc2 was used as a loading control. Right: For3 expression levels are expressed as mean relative units ± SD and correspond to experiments performed as biological triplicates. (**B**) Total ring assembly and contraction times (left) and ring constriction rates (μm/min) (right) were estimated by time-lapse confocal fluorescence microscopy for the indicated strains growing exponentially in YES-glycerol medium with or without 0.16 mM GSH. *n* is the total number of cells scored from three independent experiments, and data are presented as violin plots. ***, p<0.005; *, p<0.05; ns, not significant, as calculated by unpaired Student’s *t*-test. (**C**) Representative maximum-projection time-lapse images of Rlc1 dynamics at the equatorial region in cells from the indicated strains growing in YES-glycerol with or without 0.16 mM GSH. Mitotic progression was monitored using Pcp1-GFP-labeled SPBs. Time interval is 5 min. Scale bar: 10 µm. (**D**) Decimal dilutions of strains of the indicated genotypes were spotted onto plates with YES-glucose or YES-glycerol plates with or without 0.16 mM GSH, incubated at 28 °C for three (Glc) or five (Gly) days, and photographed. The images correspond to a representative experiment that was repeated at least three times with similar results. Figure 6—source data 1.Source data for Figure 6. Figure 6—source data 2.Western blot images for Figure 6A.

Animal cells produce reactive oxygen species (ROS) during aerobic respiration due to electron leakage from mitochondria (Ostrow et al., 1994). Oxidative stress from free radicals produced during respiration in *S. pombe* (Malina et al., 2021) activates Sty1 and antioxidant responses at both transcriptional and translational levels (Figure 6A; Zambon et al., 2017). Remarkably, 0.16 mM of the antioxidant tripeptide reduced glutathione (GSH) in the growth medium counteracted many effects of oxidative stress in *rlc1-S35A* and *myo2-E1* cells grown in glycerol. Changes included recovery of For3 levels (Figure 6A), the cable-to-patch ratio (Figure 6—figure supplement 1A), the timing of CAR assembly/contraction and constriction rate (Figure 6B–C), septation (Figure 6—figure supplement 1B) and growth (Figure 6D). Normal growth depended on For3 (Figure 6D). In contrast, in *wis1∆* cells, GSH had no significant effect on the above-mentioned characteristics (Figure 6B–D). Hence, respiration-induced oxidative stress reduces the nucleation of actin cables by formins and renders the execution of cytokinesis dependent on the phosphorylation of the Myo2 light chain Rlc1.

**Figure 6—figure supplement 1.**
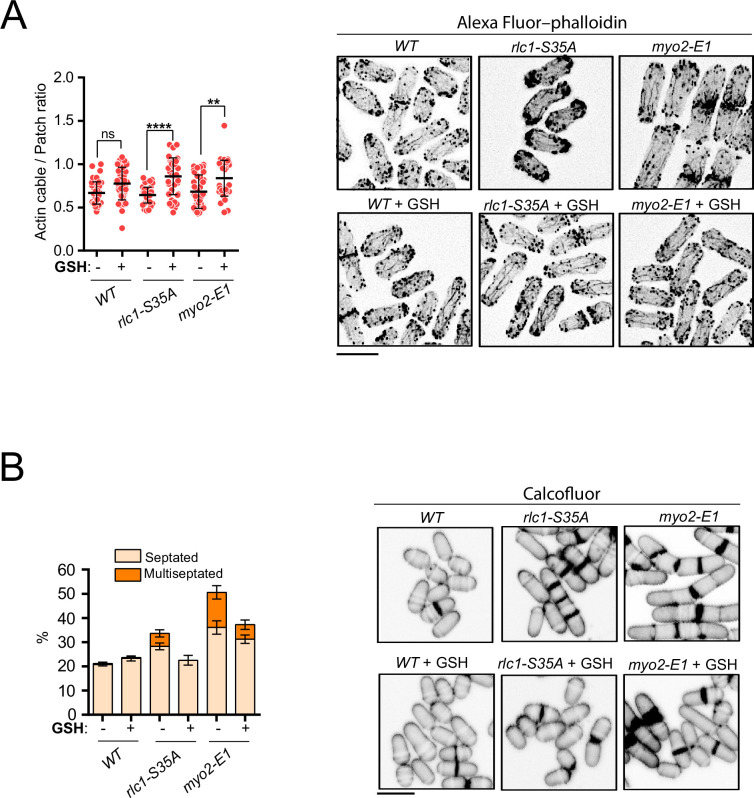
Reduced glutathione restores actin cables and alleviates septation defects in *rlc1-S35A* and *myo2-E1* cells during respiration. (**A**) Left: Actin cable to patch ratio of G2 cells from the indicated strains growing in YES-glycerol with or without 0.16 mM GSH. Quantification data (n=41 cells for each strain), are represented as mean relative units ± SD. ****, p<0.0001; **, p<0.01; ns, not significant, as calculated by unpaired Student’s *t*-test. Right: representative maximum projection images of Alexa Fluor–phalloidin stained *S. pombe* cells of the indicated strains grown for 12 hr in YES-glycerol medium with or without 0.16 mM GSH. Scale bar: 10 µm. (**B**) Left: the percentage of septated and multiseptated cells was quantified in the indicated strains grown for 12 hr in YES-glycerol medium with or without 0.16 mM GSH. Data correspond to three independent experiments and are presented as mean ± SD. Right: representative maximum projection confocal images of cells grown in YES-glycerol after cell-wall staining with calcofluor white. Figure 6—figure supplement 1—source data 1.Source data for Figure 6—figure supplement 1.

**Figure 6—figure supplement 2.**
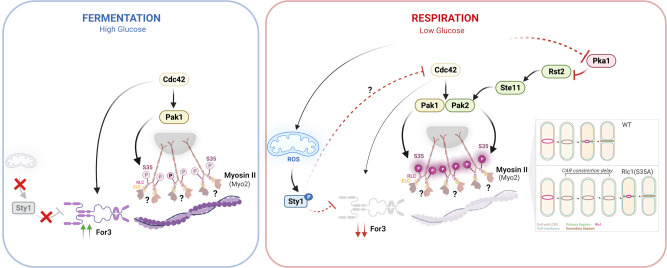
Model depicting the signaling pathways and mechanisms that regulate *S*. *pombe* cytokinesis by myosin II (Myo2) through regulatory light chain phosphorylation during respiratory metabolism. See text for specific details.

## Discussion

RLC phosphorylation regulates myosin II activity in both muscle and non-muscle cells. It plays a key positive role as a regulator of myosin II function in cardiac muscle contraction under normal and disease conditions (Yuan et al., 2015). In non-muscle vertebrate cells, RLC phosphorylation at Ser19 is essential for NMII contractile activity during cell migration and division (Garrido-Casado et al., 2021; Komatsu et al., 2000). In *Drosophila melanogaster*, in vivo phosphorylation of *spaghetti-squash* RLC at the conserved Ser21 is critical for myosin II activation, preventing embryonic lethality and severe cytokinesis defects (Jordan and Karess, 1997). Conversely, in the unicellular amoeba *Dictyostelium discoideum* RLC phosphorylation at the conserved Ser13 is not essential for myosin II function (Ostrow et al., 1994).

The role of RLC phosphorylation on myosin II activity during cytokinesis in the fission yeast *S. pombe* has remained elusive. While some studies suggest that lack of Rlc1 phosphorylation at the conserved Ser35 delays CAR constriction (Pollard et al., 2017; Loo and Balasubramanian, 2008), others indicate the opposite (Sladewski et al., 2009). *S. pombe* uses aerobic fermentation instead of respiration for ATP production when glucose is available, while mitochondrial energy metabolism is significantly reduced (Malina et al., 2021). To our knowledge, all published studies investigating the mechanistic insights of fission yeast cytokinesis have been performed in glucose-fermenting cells. Here, we show that Rlc1 phosphorylation plays a modest role during cytokinesis in the presence of glucose, but becomes essential when *S. pombe* switches to respiratory metabolism. In this metabolic state, lack of Rlc1 phosphorylation at Ser35 results in a significant delay in CAR assembly and constriction, leading to multiseptation and limited growth using the respiratory carbon source glycerol. To allow cytokinesis during respiration, Ser35-phosphorylated Rlc1 targets Myo2, the major myosin II heavy chain involved in fission yeast CAR assembly and constriction (Zambon et al., 2017). Accordingly, the cytokinetic defects of cells expressing the hypomorphic allele *myo2-E1,* which exhibits reduced ATPase activity and actin-filament binding (Wang et al., 2020), are exacerbated during respiration and resemble those of *rlc1-S35A* cells. Therefore, respiratory carbohydrate metabolism dictates the biological relevance of Rlc1 phosphorylation in modulating Myo2 activity during *S. pombe* cytokinesis.

The p21-activated kinase Pak1 phosphorylates Rlc1 at Ser35 in vivo in a glucose-rich medium (Loo and Balasubramanian, 2008; Magliozzi et al., 2020). However, evidence from this work supports that Pak2 phosphorylates Rlc1 at this residue together with Pak1 for adequate CAR contractility during respiration. Accordingly, cytokinetic and respiratory growth defects similar to those reported for *rlc1-S35A* cells were observed in the absence of both Pak1 and Pak2 activity. Pak2 expression is induced during respiratory growth by a transcriptional mechanism involving the Ste11 transcription factor. In turn, Ste11 expression is activated by the Rst2 transcription factor, whose activity is repressed by the cAMP-PKA pathway in the presence of glucose (Kunitomo et al., 2000), making Pak2 available during respiration. In this situation, Pak2 may boost PAK activity by acting on multiple targets, some of them redundantly with Pak1. In addition to Rlc1, phosphoproteomic screens have identified other Pak1 substrates that are functional during cytokinesis and polarized growth (Magliozzi et al., 2020). However, since Pak2 only localizes to the CAR during respiratory growth, its functional redundancy with Pak1 might be limited to cytokinesis-associated proteins.

In animal cells, both de novo actin disposal at the division site and cortical actin transport/flow contribute to CAR assembly (Cao and Wang, 1990; Khaliullin et al., 2018; White and Borisy, 1983). In fission yeast cells, which lack an actin filament cortex, the CAR is mainly assembled by Myo2 from actin filaments nucleated de novo at the cytokinesis nodes by the essential formin Cdc12, and partly from Cdc12-nucleating actin cables pulled from the non-equatorial zone (Huang et al., 2012; Pelham and Chang, 2002). For3, the formin that assembles the actin cables involved in polarized secretion and growth, also contributes to CAR formation in fission yeast (Coffman et al., 2013; Gómez-Gil et al., 2020). In turn, the activated SAPK pathway down-regulates CAR assembly and stability in response to stress by reducing For3 levels (Gómez-Gil et al., 2020; Madrid et al., 2021). Like animal cells, fission yeast cells undergo endogenous oxidative stress during respiration (Farrugia and Balzan, 2012), which leads to Sty1 activation and downregulation of For3 levels and actin filament availability. Hence, in this metabolic context, the regulation of Myo2 function by Rlc1 phosphorylation during cytokinesis becomes critical due to a decrease in For3-nucleated actin filaments. Accordingly, restoring actin filament availability by various strategies, including the expression of a constitutively active For3 version, limiting For3 downregulation in SAPK-less mutants, or attenuating endogenous oxidative stress with antioxidants (GSH), rescued cytokinesis and cell growth during respiration in both *rlc1-S35A* and *myo2-E1* mutants. The number of actin filaments at the CAR is reduced by approximately half in *myo2-E1* cells (Malla et al., 2022). Enhanced nucleation of actin filaments by For3 may therefore alleviate their defective actin-binding and motor activity during cytokinesis.

Metabolic reprogramming drives the actin cytoskeletal rearrangements that occur during cell response to external forces, epithelial-to-mesenchymal transition, and cell migration (Bays et al., 2017; Shiraishi et al., 2015; DeWane et al., 2021). However, an open question is how changes in cell metabolism trigger actin cytoskeleton remodeling (DeWane et al., 2021). Our observations reveal a sophisticated adaptive interplay between modulation of myosin II function by Rlc1 phosphorylation and environmentally controlled formin availability, which becomes critical for a successful cytokinesis during a respiratory carbohydrate metabolism (Figure 6—figure supplement 2). Collectively, these findings provide a remarkable example of how carbohydrate metabolism dictates the relative importance of different sources of actin filaments for CAR dynamics during cellular division.

## Materials and methods

**Key resources table:** 

Reagent type (species) or resource	Designation	Source or reference	Identifiers	Additional information
Antibody	anti-Phospho-p38 (rabbit polyclonal)	Cell Signaling	Cat# 9211, RRID:AB_331641	WB (1:1000)
Antibody	anti-HA (mouse monoclonal)	Roche	Cat# 11 583 816 001, RRID:AB_514505	WB (1:1000)
Antibody	anti-GFP (mouse monoclonal)	Roche	Cat# 11 814 460 001, RRID:AB_390913	WB (1:1000)
Antibody	HRP-conjugated anti-HA antibody (rat monoclonal)	Roche	Cat# 12 013 819 001, RRID:AB_390917	WB (1:3000)
Antibody	anti-Cdk1/Cdc2 (PSTAIR)(rabbit polyclonal)	Millipore	Cat#: 06–923; RRID:AB_310302	WB (1:1000)
Antibody	anti-Mouse IgG- peroxidase (goat polyclonal)	Sigma Aldrich	Cat#: A5278; RRID:AB_258232	WB (1:2000)
Antibody	anti-Rabbit IgG- peroxidase (goat polyclonal)	Sigma Aldrich	Cat#: A6667; RRID:AB_258307	WB (1:2000)
Commercial assay, kit	ECL Western Blotting Reagents	GE-Healthcare	Cat#: RPN2106	
Chemical compound, drug	β-estradiol	Sigma Aldrich	Cat#: E2758	10–500 µM
Chemical compound, drug	PhosTag acrylamide	Wako Chemical	Cat#: 300–93523	15 µM
Chemical compound, drug	PP1 Analog III, 3-MB-PP1	Sigma Aldrich	Cat#: 529582	1 μM
Chemical compound, drug	Alexa fluor 488-conjugated phalloidin	Thermo Fischer Scientific	Cat#: A12379	200 units/ml (~6.6 µM)
Chemical compound, drug	Soybean lectin	Sigma Aldrich	Cat#: L2650	1 mg/ml
Software, algorithm	ImageJ	ImageJ	https://imagej.net/Fiji/Downloads	Quantification of Western blots and microscopic analysis
Software, algorithm	Graphpad Prism 9.0.2	Graphpad	https://www.graphpad.com/scientific-software/prism//	Statistical analysis and graphs representation
Software, algorithm	Ilastik	Ilastik	https://www.ilastik.org/	Segmentation toolkit
Other	μ-Slide 8 well	Ibidi	Cat#: 80826	For time-lapse imaging of CAR dynamics. (Microscopy analysis; Materials and Methods section)

### Strain construction

*S. pombe* strains used in this work are listed in Supplementary file 1. Several deletion strains were obtained from the Bioneer mutant library (Kim et al., 2010), while null mutants in the *rlc1^+^, pka1*^+^, *ste11*^+^, and *rst2*^+^ genes were obtained by ORF deletion and replacement with G418 (kanR), nourseothricin (NAT), or hygromycin B cassettes using a PCR-mediated strategy (Hentges et al., 2005; Sato et al., 2005) and the oligonucleotides described in . Strains expressing different genomic fusions were constructed either by transformation or by random spore analysis of appropriate crosses in sporulation agar (SPA) medium (Petersen and Russell, 2016). To generate a strain expressing an integrated Rlc1-HA fusion, the *rlc1^+^* + plus its endogenous promoter was amplified by PCR using genomic DNA from *S. pombe* 972h^-^ wild-type strain as a template, and the 5’ and 3´oligonucleotides PromRlc1(XhoI)-FWD and Rlc1-HA(SacII)-REV (Supplementary file 2), which contain a *Xho*I restriction site and an extended DNA sequence encoding a HA C-terminal tag plus a *Sac*II site, respectively. The *Xho*I-*Sac*II digested PCR fragment was cloned into plasmid pJK210 (Keeney and Boeke, 1994), sequenced, linearized with *Bmg*BI, and transformed into an *rlc1Δ ura4.294* strain. To obtain a strain expressing an integrative Rlc1-HA fusion under the control of the β-estradiol promoter (Ohira et al., 2017), the *rlc1^+^* ORF fused to a 3 + HA tag was amplified by PCR using the 5’ and 3-oligonucleotides Rlc1 (SmaI)- FWD and Rlc1-HA (SacII)-REV, containing *Sma*I and *Sac*II restriction sites, respectively. The amplified PCR product was cloned into a modified plasmid pJK210 containing a β-estradiol regulated promoter Z_3_EV (Ohira et al., 2017), and the resulting construct was linearized with *Stu*I, and transformed into an *rlc1Δ ura4.294* strain. To obtain a strain expressing an integrative Rlc1-GFP fusion, DNA encoding an Rlc1-GFP fusion under the endogenous promoter was amplified by PCR using as a template genomic DNA from a *S. pombe* strain expressing a genomic Rlc1-GFP fusion (Supplementary file 1) and the 5’ and 3’ oligonucleotides PromRlc1(XhoI)-FWD and Rlc1-GFP(SacII)-REV containing *Sma*I and *Sac*II restriction sites, respectively. In all cases, *ura4^+^* + were obtained, and the correct integration and expression of the Rlc1-HA and Rlc1-GFP fusions under either the endogenous or the β-estradiol regulated promoters were verified by both PCR and Western blot analysis. To generate strains expressing Rlc1-GFP versions with mutations at residues Ser35 and Ser36 to alanine or aspartic acid, the pJK210 plasmid containing the Rlc1-GFP fusion was used as a template for site-directed mutagenesis by PCR, using specific mutagenic oligonucleotides described in Supplementary file 2. The mutagenized plasmids were then linearized with *Bmg*BI and transformed into an *rlc1Δ ura4.294* strain.

The *S. pombe* strain expressing a genomic Pak2-3GFP fusion was obtained in two consecutive steps. First, the *pak2^+^* + plus its endogenous promoter was amplified by PCR using genomic DNA from the *S. pombe* 972h^-^ wild-type strain as a template, and the 5’ and 3’ oligonucleotides PromPak2(XhoI)-FWD (*Xho*I site) and Pak2GFP(SmaI/XmaI)-REV (*Sma*I site) (Supplementary file 2). The PCR product was cloned in a frame into a pJK210 plasmid containing a GFP C-terminal tag. In a second step, this construct was linearized with *Sma*I and two additional GFP tags were added by a Gibson assembly approach. Finally, the resulting plasmid was linearized with *Bmg*BI and transformed into a *pak2Δ ura4.294* strain. To introduce the mutations at the two putative Ste11-binding motifs (TR box) in the Pak2 promoter, the pJK210-Pak2-3GFP plasmid was subjected to sequential site-directed mutagenesis by PCR. In this way, the conserved G in each motif was replaced by A by using the mutagenic oligonucleotides described in Supplementary file 2. To generate a strain that produces a Pak2-GFP fusion under the control of Pak1 promoter, the Pak1 5’-UTR sequence was amplified by PCR using genomic DNA from the wild-type *S. pombe* 972h^-^ strain, and assembled by Gibson cloning to a PCR-amplified Pak2-GFP fragment and the pJK210 plasmid linearized with *Sma*I. The resulting plasmid was digested with *Bmg*BI and transformed into a *pak2Δ ura4.294* strain.

### Media and growth conditions

In liquid culture experiments, fission yeast strains were grown overnight with shaking at 28 °C in YES-glucose medium containing 0.6% yeast extract, 2% glucose and supplemented with adenine, leucine, histidine, or uracil (100  mg/liter) (Prieto-Ruiz et al., 2020). The next day, cultures were diluted to an OD_600_ of 0.01 and incubated to a final OD_600_ of 0.2. Then, cells were recovered by filtration, washed three times, and shifted to either YES-glucose or YES-glycerol (0.6% yeast extract, 0.08% glucose, 0.86% glycerol, plus supplements), and incubated at 28 °C for 4 or 8 hr before imaging. In experiments performed with the *myo2-E1* mutant, cells harvested from cultures at 28 °C were resuspended in YES-glucose or YES-glycerol, incubated at 30 °C for 2 hr, and then at 28 °C for the remainder of the experiment. In experiments with cells expressing the analog-sensitive Cdc2 (CDK) kinase version *cdc2-asM17* (Aoi et al., 2014), cells from log-phase liquid cultures in YES-glucose (OD_600_ 0.5), were treated with 1 µM 3-NM-PP1 (Sigma-Aldrich, 529581) for 3.5 hr, recovered by filtration, washed, and resuspended in YES-glucose medium. In experiments with strains expressing an analog-sensitive Pak1 kinase version *pak1-M460A*, log-phase liquid cultures were split in two and incubated for different times in YES-glucose medium treated with 10 µM 3-BrB-PP1 (Abcam, ab143756), or in medium lacking the analog kinase inhibitor. For nitrogen starvation experiments, strains growing exponentially in Edinburgh Minimal Medium (EMM2) (Moreno et al., 1991) containing 2% glucose (OD_600_ 0.5), were recovered by filtration and resuspended in the same medium without ammonium chloride for the indicated times. For the plate assays of growth stress sensitivity, *S. pombe* wild-type and mutant strains were grown in YES-glucose liquid medium to an OD_600_ of 1.2, recovered by centrifugation, resuspended in YES to a density of 10^7^ cells/ml, and appropriate decimal dilutions were spotted on YES-glucose, or YES-glycerol solid plates (2% agar). The plates were incubated for 3  days (YES-glucose) or 5 days (YES-glycerol), at different temperatures (28 °C, 30 °C, 32 °C, and/or 34 °C), depending on the experiment, and then photographed. All the assays were repeated at least three times with similar results. Representative experiments are shown in the corresponding figures. When required, solid and/or liquid media were supplemented with varying amounts of β-estradiol (Sigma-Aldrich, RPN2106) or reduced glutathione (GSH; Sigma-Aldrich, G6013).

### Microscopy analysis

For *time-lapse* imaging of CAR dynamics, 300 µl of cells grown exponentially for 4 hr in YES-glucose or YES-glycerol liquid medium, and prepared as described above, were added to one well of a μ-Slide eight well chamber (Ibidi, 80826) previously coated with 10 μl of 1 mg/ml soybean lectin (Sigma-Aldrich, L2650) (Gómez-Gil et al., 2020). GSH was added to the medium as required, to at a final concentration of 0.3 mM. Cells were allowed to sediment in the culture media and adhere to the bottom of the well for 1 min, and images were taken every 2.5 min for 2 hr in YES-glucose cultures or every 5 min for 8 hr in YES-glycerol cultures. Experiments were performed at 28 °C, and single mid-planes were taken from a set of six stacks (0.61 µm each) at the indicated time points. Time-lapse images were acquired using a Leica Stellaris eight confocal microscope with a 63 X/1.40 Plan Apo objective controlled by the LAS X software. The time for node condensation and ring maturation includes the time from SPB separation to the onset of CR constriction. Ring constriction and disassembly time include the time from the first frame of ring constriction to the last frame where the ring is completely constricted and disassembled. The total time for ring assembly and contraction is the sum of these two values. *n* is the total number of cells scored from at least three independent experiments. Ring constriction rates were measured manually as the average circumference ± SD of wild-type and mutant Rlc1-GFP versions at contractile rings in each time frame starting from the first frame of CAR constriction. n=30 cells were measured for each strain and growth condition. Statistical comparison between the two groups was performed by unpaired Student’s *t-test*.

For actin staining with Alexa-Fluor phalloidin, 5 ml mid-log cultures were grown in YES-glucose (OD_600_ 0.5) or YES-glycerol (OD_600_ 0.2) for 12 hr after the media shift. Cells were fixed by immersion for 1 h in 3.7% formaldehyde in PEM buffer (10 mM EGTA; 1 mM MgSO_4_; 100 mM PIPES pH 6.9, 75 mM sucrose, and 0.1% Triton X-100). After three washes with PEM, the cell pellets were resuspended in 20 µl of cold 40% methanol solution, stained with 8 µl of 5 mg/ml Alexa Fluor 488-conjugated phalloidin (Thermo Fisher Scientific, A12379), and incubated overnight at 4 °C in the dark on a rotary platform. Images of stained cells were captured from samples spotted on glass slides using a Leica Stellaris eight confocal microscope with a 100 X/1.40 Plan Apo objective (seven stacks of 0.3 µm each). For actin segmentation analysis, the Ilastik routine using the pixel classification tool (Berg et al., 2019) was trained with two representative images, one of the cells growing in YES-glucose medium and one of the cells growing in YES-glycerol. Training involved drawing cables, patches, and backgrounds in three different colors. Once the program was trained, the remaining images from the different experiments were uploaded to Ilastik to perform the segmentation routine. The resulting images were then exported to ImageJ (Schneider et al., 2012) and the segmented cells at G2 were analyzed using the color histogram tool to obtain the specific areas corresponding to cables and patches. Data from n≥40 cells growing in YES-glycerol were obtained for each cell by dividing the cable area by the patch area, and the ratio was normalized with respect to the average obtained from wild-type cells growing in YES-glucose medium. For For3-GFP quantification, Ilastik was trained by drawing For3-GFP dots, GFP background, and image background in three different colors. The ratio of For3-GFP spots to cytosol was calculated by dividing the area of For3-GFP spots by the area of GFP background of at least n≥40 cells in G2 or late M (dividing cells) and normalized to the average of the glucose ratio.

For cell wall staining, cells were recovered from 1 ml aliquots by centrifugation, stained with 1 µl of 0.5 mg/ml calcofluor white, and images were acquired from samples spotted on glass slides using a Leica Stellaris 8 confocal microscope with a 63 X/1.40 Plan Apo objective (six stacks of 0.61 µm each). The percentage of septated (one septa), multiseptated (two or more septa), and lysed cells was calculated at the indicated time points for each strain and condition from three independent experiments. n≥100 cells were counted from multiple images taken during each replicate.

### Western blot analysis

To determine the level of Rlc1-GFP fusion and/or its phosphorylation status, 10 ml samples of fission yeast cultures were collected and precipitated with TCA (Grallert and Hagan, 2017). Protein extracts were resolved on 12% SDS-PAGE gels containing 30 μM Phos-tag acrylamide (Wako, AAL-107), transferred to nitrocellulose blotting membranes, and immunoblotted with a mouse monoclonal anti-GFP antibody (Roche, 11 814 460 001, RRID:AB_390913). Rabbit monoclonal anti-PSTAIR (anti-Cdc2; Sigma-Aldrich, 06–923, RRID:AB_310302) was used as a loading control. Immunoreactive bands were detected using anti-mouse (Abcam, ab205719, RRID:AB_2755049), and anti-rabbit HRP-conjugated secondary antibodies (Abcam, ab205718, RRID:AB_2819160), and the ECL system (GE-Healthcare, RPN2106). For the detection of Pak1-GFP, Pak2-3GFP, For3-3GFP, and For3(DAD)–2GFP fusions, protein extracts obtained after TCA-precipitation (Pak1-GFP and Pak2-3GFP fusions) or under native conditions (For3-3GFP and For3(DAD)–2GFP fusions) were resolved on 6% SDS-PAGE gels, transferred to Hybond-ECL membranes, and incubated with a mouse monoclonal anti-GFP antibody (Roche) and anti-cdc2 (PSTAIR) as a loading control. In all cases, the immunoreactive bands were revealed using anti-mouse or anti-rabbit HRP-conjugated secondary antibodies and the ECL system. Detection of Sty1 phosphorylation and total protein levels in strains expressing a genomic Sty1-HA fusion was performed exactly as described in Gómez-Gil et al., 2020. Dual phosphorylation of Sty1 was detected using a rabbit polyclonal anti-phospho-p38 antibody (Cell Signaling, 9211, RRID:AB_331641). Total Sty1 was detected in *S. pombe* extracts using a mouse monoclonal anti-HA antibody (12CA5, Roche). Immunoreactive bands were detected using anti-mouse or anti-rabbit HRP-conjugated secondary antibodies (Abcam) and the ECL system.

Densitometric quantification of Western blot experiments from 16-bit. jpg digital images of blots were performed using ImageJ (Schneider et al., 2012). The bands of interest plus background were drawn as rectangles and a profile plot (peak) was generated for each band. To reduce background noise in the bands, each peak above the baseline was manually closed using the straight-line tool. Measurement of the closed peaks was performed using the wand tool. Relative units (R.U.) of For3 levels were estimated by determining the signal ratio of the corresponding anti-GFP (total For3) blot with respect to the anti-cdc2 blot (internal control) at each time point. Quantification data correspond to experiments performed as biological triplicates. Mean relative units ± SD is shown.

### Statistical analysis

Statistical analysis was performed using prism 9.0.2. software (GraphPad), and results are represented as violin plots or mean ± SD, unless otherwise stated. Comparisons between two groups were calculated using unpaired two-tailed Student’s t-tests, whereas comparisons between more than two groups were calculated using one-way ANOVA with Bonferroni’s multiple comparison test. We observed normal distribution and no difference in variance between groups in individual comparisons. Statistical significance: *p<0.05; **p<0.005; ***p<0.0005; ****p<0.0001. Further details of statistical analysis are given in the figure legends.

## Data Availability

All data generated or analysed during this study are included in the manuscript and supporting files.

## Additional files

MDAR checklist

Supplementary file 1. S.pombe strains used in this study.

Supplementary file 2. Oligonucleotides and DNA fragments used in this study.

## References

[bib1] Alonso-Matilla R, Thiyagarajan S, O’Shaughnessy B (2019). Sliding filament and fixed filament mechanisms contribute to ring tension in the cytokinetic contractile ring. Cytoskeleton.

[bib2] Aoi Y, Kawashima SA, Simanis V, Yamamoto M, Sato M (2014). Optimization of the analogue-sensitive cdc2/cdk1 mutant by in vivo selection eliminates physiological limitations to its use in cell cycle analysis. Open Biology.

[bib3] Balasubramanian MK, Bi E, Glotzer M (2004). Comparative analysis of cytokinesis in budding yeast, fission yeast and animal cells. Current Biology.

[bib4] Bao J, Jana SS, Adelstein RS (2005). Vertebrate nonmuscle myosin II isoforms rescue small interfering RNA-induced defects in COS-7 cell cytokinesis. The Journal of Biological Chemistry.

[bib5] Bays JL, Campbell HK, Heidema C, Sebbagh M, DeMali KA (2017). Linking E-cadherin mechanotransduction to cell metabolism through force-mediated activation of AMPK. Nature Cell Biology.

[bib6] Berg S, Kutra D, Kroeger T, Straehle CN, Kausler BX, Haubold C, Schiegg M, Ales J, Beier T, Rudy M, Eren K, Cervantes JI, Xu B, Beuttenmueller F, Wolny A, Zhang C, Koethe U, Hamprecht FA, Kreshuk A (2019). Ilastik: interactive machine learning for (bio)image analysis. Nature Methods.

[bib7] Cao LG, Wang YL (1990). Mechanism of the formation of contractile ring in dividing cultured animal cells. I. recruitment of preexisting actin filaments into the cleavage furrow. The Journal of Cell Biology.

[bib8] Chang F, Drubin D, Nurse P (1997). Cdc12p, a protein required for cytokinesis in fission yeast, is a component of the cell division ring and interacts with profilin. The Journal of Cell Biology.

[bib9] Coffman VC, Sees JA, Kovar DR, Wu JQ (2013). The formins CDC12 and for3 cooperate during contractile ring assembly in cytokinesis. The Journal of Cell Biology.

[bib10] Craig R, Smith R, Kendrick-Jones J (1983). Light-Chain phosphorylation controls the conformation of vertebrate non-muscle and smooth muscle myosin molecules. Nature.

[bib11] DeWane G, Salvi AM, DeMali KA (2021). Fueling the cytoskeleton - links between cell metabolism and actin remodeling. Journal of Cell Science.

[bib12] Farrugia G, Balzan R (2012). Oxidative stress and programmed cell death in yeast. Frontiers in Oncology.

[bib13] Garrido-Casado M, Asensio-Juárez G, Vicente-Manzanares M (2021). Nonmuscle myosin II regulation directs its multiple roles in cell migration and division. Annual Review of Cell and Developmental Biology.

[bib14] Gómez-Gil E, Martín-García R, Vicente-Soler J, Franco A, Vázquez-Marín B, Prieto-Ruiz F, Soto T, Pérez P, Madrid M, Cansado J (2020). Stress-activated MAPK signaling controls fission yeast actomyosin ring integrity by modulating formin for3 levels. eLife.

[bib15] Grallert A, Hagan IM (2017). Preparation of protein extracts from *Schizosaccharomyces pombe* using trichloroacetic acid precipitation. Cold Spring Harbor Protocols.

[bib16] Green RA, Paluch E, Oegema K (2012). Cytokinesis in animal cells. Annual Review of Cell and Developmental Biology.

[bib17] Hentges P, Van Driessche B, Tafforeau L, Vandenhaute J, Carr AM (2005). Three novel antibiotic marker cassettes for gene disruption and marker switching in *Schizosaccharomyces pombe*. Yeast.

[bib18] Huang J, Huang Y, Yu H, Subramanian D, Padmanabhan A, Thadani R, Tao Y, Tang X, Wedlich-Soldner R, Balasubramanian MK (2012). Nonmedially assembled F-actin cables incorporate into the actomyosin ring in fission yeast. The Journal of Cell Biology.

[bib19] Jordan P, Karess R (1997). Myosin light chain-activating phosphorylation sites are required for oogenesis in *Drosophila*. The Journal of Cell Biology.

[bib20] Keeney JB, Boeke JD (1994). Efficient targeted integration at leu1-32 and ura4-294 in *Schizosaccharomyces pombe*. Genetics.

[bib21] Khaliullin RN, Green RA, Shi LZ, Gomez-Cavazos JS, Berns MW, Desai A, Oegema K (2018). A positive-feedback-based mechanism for constriction rate acceleration during cytokinesis in *Caenorhabditis elegans*. eLife.

[bib22] Kim D-U, Hayles J, Kim D, Wood V, Park H-O, Won M, Yoo H-S, Duhig T, Nam M, Palmer G, Han S, Jeffery L, Baek S-T, Lee H, Shim YS, Lee M, Kim L, Heo K-S, Noh EJ, Lee A-R, Jang Y-J, Chung K-S, Choi S-J, Park J-Y, Park Y, Kim HM, Park S-K, Park H-J, Kang E-J, Kim HB, Kang H-S, Park H-M, Kim K, Song K, Song KB, Nurse P, Hoe K-L (2010). Analysis of a genome-wide set of gene deletions in the fission yeast *Schizosaccharomyces pombe*. Nature Biotechnology.

[bib23] Komatsu S, Yano T, Shibata M, Tuft RA, Ikebe M (2000). Effects of the regulatory light chain phosphorylation of myosin II on mitosis and cytokinesis of mammalian cells. The Journal of Biological Chemistry.

[bib24] Kovar DR, Kuhn JR, Tichy AL, Pollard TD (2003). The fission yeast cytokinesis formin cdc12p is a barbed end actin filament capping protein gated by profilin. The Journal of Cell Biology.

[bib25] Kunitomo H, Higuchi T, Iino Y, Yamamoto M (2000). A zinc-finger protein, rst2p, regulates transcription of the fission yeast ste11(+) gene, which encodes A pivotal transcription factor for sexual development. Molecular Biology of the Cell.

[bib26] Laplante C, Berro J, Karatekin E, Hernandez-Leyva A, Lee R, Pollard TD (2015). Three myosins contribute uniquely to the assembly and constriction of the fission yeast cytokinetic contractile ring. Current Biology.

[bib27] Laplante C, Huang F, Tebbs IR, Bewersdorf J, Pollard TD (2016). Molecular organization of cytokinesis nodes and contractile rings by super-resolution fluorescence microscopy of live fission yeast. PNAS.

[bib28] Laporte D, Coffman VC, Lee IJ, Wu JQ (2011). Assembly and architecture of precursor nodes during fission yeast cytokinesis. The Journal of Cell Biology.

[bib29] Le Goff X, Motegi F, Salimova E, Mabuchi I, Simanis V (2000). The *S. pombe* rlc1 gene encodes a putative myosin regulatory light chain that binds the type II myosins myo3p and myo2p. Journal of Cell Science.

[bib30] Loo TH, Balasubramanian M (2008). *Schizosaccharomyces pombe* pak-related protein, pak1p/orb2p, phosphorylates myosin regulatory light chain to inhibit cytokinesis. The Journal of Cell Biology.

[bib31] Ma X, Jana SS, Conti MA, Kawamoto S, Claycomb WC, Adelstein RS (2010). Ablation of nonmuscle myosin II-B and II-C reveals a role for nonmuscle myosin II in cardiac myocyte karyokinesis. Molecular Biology of the Cell.

[bib32] Madrid M, Soto T, Franco A, Paredes V, Vicente J, Hidalgo E, Gacto M, Cansado J (2004). A cooperative role for atf1 and pap1 in the detoxification of the oxidative stress induced by glucose deprivation in *Schizosaccharomyces pombe*. The Journal of Biological Chemistry.

[bib33] Madrid M, Gómez-Gil E, Cansado J (2021). Negative control of cytokinesis by stress-activated MAPK signaling. Current Genetics.

[bib34] Magliozzi JO, Sears J, Cressey L, Brady M, Opalko HE, Kettenbach AN, Moseley JB (2020). Fission yeast pak1 phosphorylates anillin-like mid1 for spatial control of cytokinesis. The Journal of Cell Biology.

[bib35] Malecki M, Bitton DA, Rodríguez-López M, Rallis C, Calavia NG, Smith GC, Bähler J (2016). Functional and regulatory profiling of energy metabolism in fission yeast. Genome Biology.

[bib36] Malina C, Yu R, Björkeroth J, Kerkhoven EJ, Nielsen J (2021). Adaptations in metabolism and protein translation give rise to the crabtree effect in yeast. PNAS.

[bib37] Malla M, Pollard TD, Chen Q (2022). Counting actin in contractile rings reveals novel contributions of cofilin and type II myosins to fission yeast cytokinesis. Molecular Biology of the Cell.

[bib38] Mangione MC, Gould KL (2019). Molecular form and function of the cytokinetic ring. Journal of Cell Science.

[bib39] Martin SG, Rincón SA, Basu R, Pérez P, Chang F (2007). Regulation of the formin for3p by cdc42p and bud6p. Molecular Biology of the Cell.

[bib40] Mata J, Bähler J (2006). Global roles of ste11p, cell type, and pheromone in the control of gene expression during early sexual differentiation in fission yeast. PNAS.

[bib41] McCollum D, Balasubramanian MK, Pelcher LE, Hemmingsen SM, Gould KL (1995). *Schizosaccharomyces pombe* cdc4+ gene encodes a novel EF-hand protein essential for cytokinesis. The Journal of Cell Biology.

[bib42] McDonald NA, Lind AL, Smith SE, Li R, Gould KL (2017). Nanoscale architecture of the *Schizosaccharomyces pombe* contractile ring. eLife.

[bib43] Moreno S, Klar A, Nurse P (1991). Molecular genetic analysis of fission yeast *Schizosaccharomyces pombe*. Methods in Enzymology.

[bib44] Naqvi NI, Wong KC, Tang X, Balasubramanian MK (2000). Type II myosin regulatory light chain relieves auto-inhibition of myosin-heavy-chain function. Nature Cell Biology.

[bib45] Ohira MJ, Hendrickson DG, Scott McIsaac R, Rhind N (2017). An estradiol-inducible promoter enables fast, graduated control of gene expression in fission yeast. Yeast.

[bib46] Okada H, Wloka C, Wu J-Q, Bi E (2019). Distinct roles of myosin-II isoforms in cytokinesis under normal and stressed conditions. IScience.

[bib47] Ostrow BD, Chen P, Chisholm RL (1994). Expression of a myosin regulatory light chain phosphorylation site mutant complements the cytokinesis and developmental defects of *Dictyostelium* RMLC null cells. The Journal of Cell Biology.

[bib48] Palani S, Chew TG, Ramanujam S, Kamnev A, Harne S, Chapa-Y-Lazo B, Hogg R, Sevugan M, Mishra M, Gayathri P, Balasubramanian MK (2017). Motor activity dependent and independent functions of myosin II contribute to actomyosin ring assembly and contraction in *Schizosaccharomyces pombe*. Current Biology.

[bib49] Pelham RJ, Chang F (2002). Actin dynamics in the contractile ring during cytokinesis in fission yeast. Nature.

[bib50] Pérez P, Cansado J (2010). Cell integrity signaling and response to stress in fission yeast. Current Protein & Peptide Science.

[bib51] Petersen J, Russell P (2016). Growth and the environment of *Schizosaccharomyces pombe*. Cold Spring Harbor Protocols.

[bib52] Pollard TD, Wu JQ (2010). Understanding cytokinesis: Lessons from fission yeast. Nature Reviews. Molecular Cell Biology.

[bib53] Pollard LW, Bookwalter CS, Tang Q, Krementsova EB, Trybus KM, Lowey S (2017). Fission yeast myosin myo2 is down-regulated in actin affinity by light chain phosphorylation. PNAS.

[bib54] Prieto-Ruiz F, Vicente-Soler J, Franco A, Gómez-Gil E, Sánchez-Marinas M, Vázquez-Marín B, Aligué R, Madrid M, Moreno S, Soto T, Cansado J (2020). RNA-binding protein rnc1 regulates cell length at division and acute stress response in fission yeast through negative feedback modulation of the stress-activated mitogen-activated protein kinase pathway. MBio.

[bib55] Rincon SA, Paoletti A (2016). Molecular control of fission yeast cytokinesis. Seminars in Cell & Developmental Biology.

[bib56] Sato M, Dhut S, Toda T (2005). New drug-resistant cassettes for gene disruption and epitope tagging in *Schizosaccharomyces pombe*. Yeast.

[bib57] Schneider CA, Rasband WS, Eliceiri KW (2012). Nih image to imagej: 25 years of image analysis. Nature Methods.

[bib58] Sells MA, Barratt JT, Caviston J, Ottilie S, Leberer E, Chernoff J (1998). Characterization of Pak2p, a pleckstrin homology domain-containing, p21-activated protein kinase from fission yeast. The Journal of Biological Chemistry.

[bib59] Shiraishi T, Verdone JE, Huang J, Kahlert UD, Hernandez JR, Torga G, Zarif JC, Epstein T, Gatenby R, McCartney A, Elisseeff JH, Mooney SM, An SS, Pienta KJ (2015). Glycolysis is the primary bioenergetic pathway for cell motility and cytoskeletal remodeling in human prostate and breast cancer cells. Oncotarget.

[bib60] Sladewski TE, Previs MJ, Lord M (2009). Regulation of fission yeast myosin-II function and contractile ring dynamics by regulatory light-chain and heavy-chain phosphorylation. Molecular Biology of the Cell.

[bib61] Stark BC, Sladewski TE, Pollard LW, Lord M (2010). Tropomyosin and myosin-II cellular levels promote actomyosin ring assembly in fission yeast. Molecular Biology of the Cell.

[bib62] Sugimoto A, Iino Y, Maeda T, Watanabe Y, Yamamoto M (1991). *Schizosaccharomyces pombe* ste11+ encodes a transcription factor with an HMG motif that is a critical regulator of sexual development. Genes & Development.

[bib63] Trybus KM, Lowey S (1984). Conformational states of smooth muscle myosin. Effects of light chain phosphorylation and ionic strength. The Journal of Biological Chemistry.

[bib64] Trybus KM (1989). Filamentous smooth muscle myosin is regulated by phosphorylation. The Journal of Cell Biology.

[bib65] Vavylonis D, Wu JQ, Hao S, O’Shaughnessy B, Pollard TD (2008). Assembly mechanism of the contractile ring for cytokinesis by fission yeast. Science.

[bib66] Wang K, Okada H, Bi E (2020). Comparative analysis of the roles of non-muscle myosin-iis in cytokinesis in budding yeast, fission yeast, and mammalian cells. Frontiers in Cell and Developmental Biology.

[bib67] White JG, Borisy GG (1983). On the mechanisms of cytokinesis in animal cells. Journal of Theoretical Biology.

[bib68] Yang P, Kansra S, Pimental RA, Gilbreth M, Marcus S (1998). Cloning and characterization of shk2, a gene encoding a novel p21-activated protein kinase from fission yeast. The Journal of Biological Chemistry.

[bib69] Yuan CC, Muthu P, Kazmierczak K, Liang J, Huang W, Irving TC, Kanashiro-Takeuchi RM, Hare JM, Szczesna-Cordary D (2015). Constitutive phosphorylation of cardiac myosin regulatory light chain prevents development of hypertrophic cardiomyopathy in mice. PNAS.

[bib70] Zambon P, Palani S, Kamnev A, Balasubramanian MK (2017). Myo2P is the major motor involved in actomyosin ring contraction in fission yeast. Current Biology.

[bib71] Zuin A, Gabrielli N, Calvo IA, García-Santamarina S, Hoe K-L, Kim DU, Park H-O, Hayles J, Ayté J, Hidalgo E (2008). Mitochondrial dysfunction increases oxidative stress and decreases chronological life span in fission yeast. PLOS ONE.

