# Supplementary figures and images for: Myosin II regulatory light chain phosphorylation and formin availability modulate cytokinesis upon changes in carbohydrate metabolism
Source: eLife. 2023 Feb 24;12:e83285. doi: 10.7554/eLife.83285 (PMC10005788; doi:10.7554/eLife.83285)

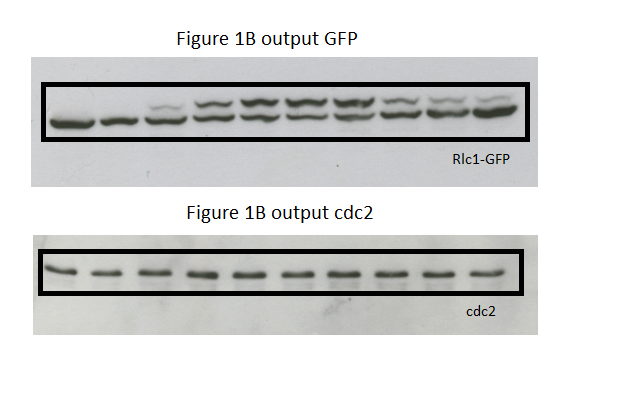

Supplement: Figure 1—source data 2. [file elife-83285-fig1-data2.zip › Figure 1B uncroped labeled gels.png]

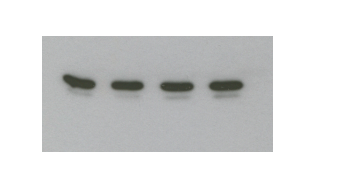

Supplement: Figure 1—source data 2. [file elife-83285-fig1-data2.zip › Figure 1A cdc2.png]

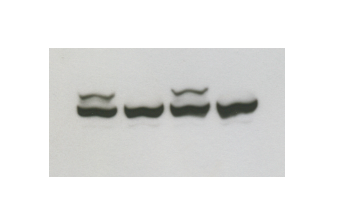

Supplement: Figure 1—source data 2. [file elife-83285-fig1-data2.zip › Figure 1A GFP.png]

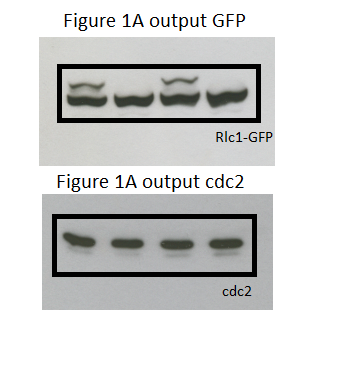

Supplement: Figure 1—source data 2. [file elife-83285-fig1-data2.zip › Figure 1A uncroped labeled gels.png]

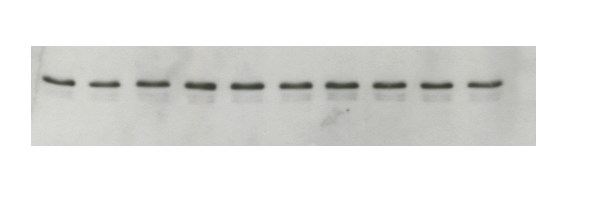

Supplement: Figure 1—source data 2. [file elife-83285-fig1-data2.zip › Figure 1B cdc2.png]

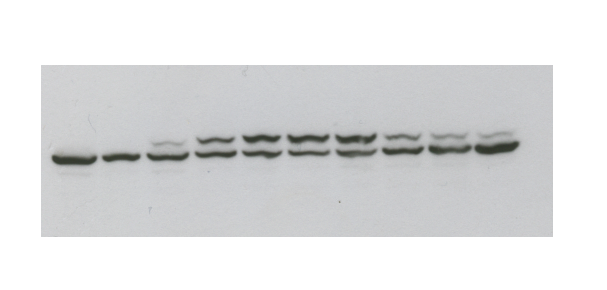

Supplement: Figure 1—source data 2. [file elife-83285-fig1-data2.zip › Figure 1B GFP.png]

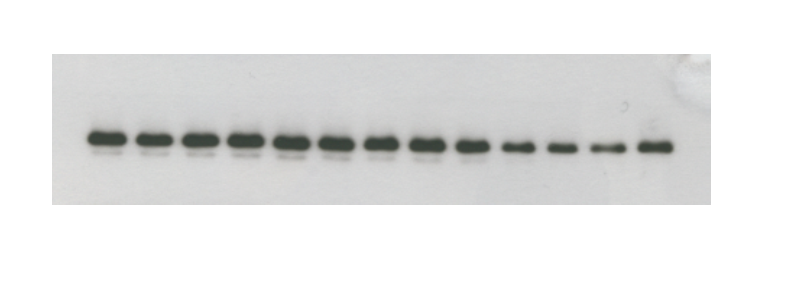

Supplement: Figure 1—figure supplement 1—source data 2. [file elife-83285-fig1-figsupp1-data2.zip › Figure 1 supplement B cdc2.png]

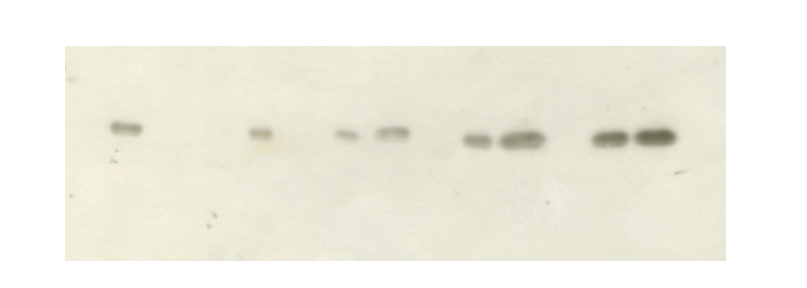

Supplement: Figure 1—figure supplement 1—source data 2. [file elife-83285-fig1-figsupp1-data2.zip › Figure 1 supplement B HA.png]

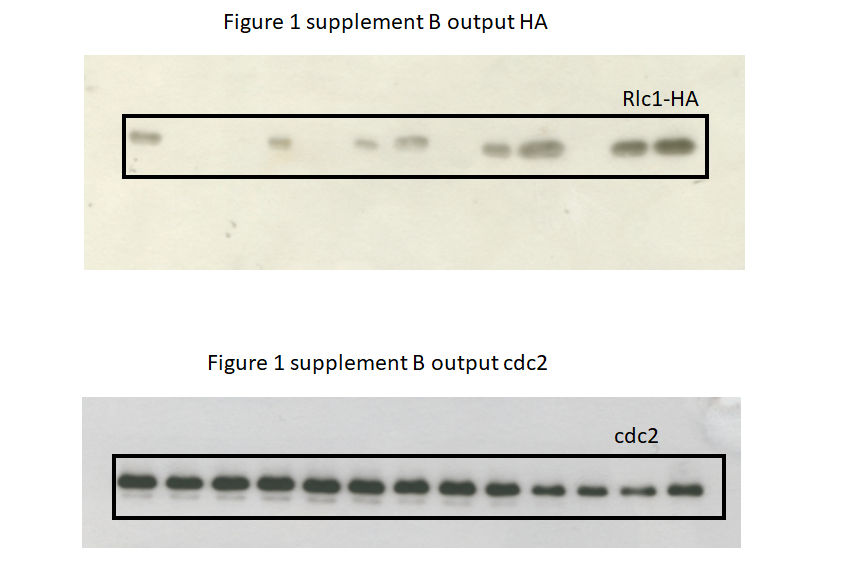

Supplement: Figure 1—figure supplement 1—source data 2. [file elife-83285-fig1-figsupp1-data2.zip › Figure 1 supplement B uncroped labeled gels.png]

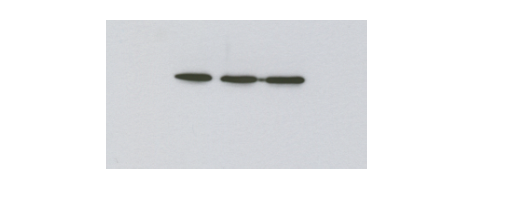

Supplement: Figure 2—source data 2. [file elife-83285-fig2-data2.zip › Figure 2D unperturbed growth.png]

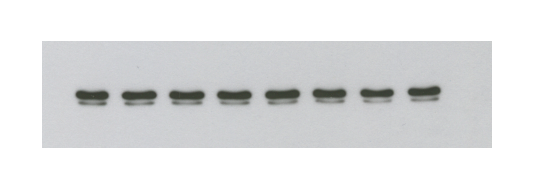

Supplement: Figure 2—source data 2. [file elife-83285-fig2-data2.zip › Figure 2A cdc2.png]

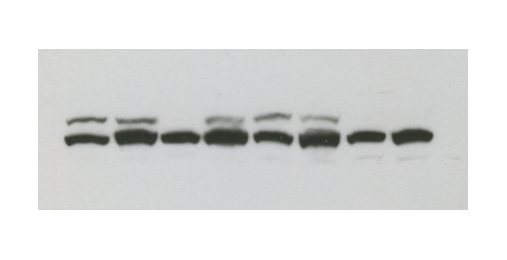

Supplement: Figure 2—source data 2. [file elife-83285-fig2-data2.zip › Figure 2A GFP.png]

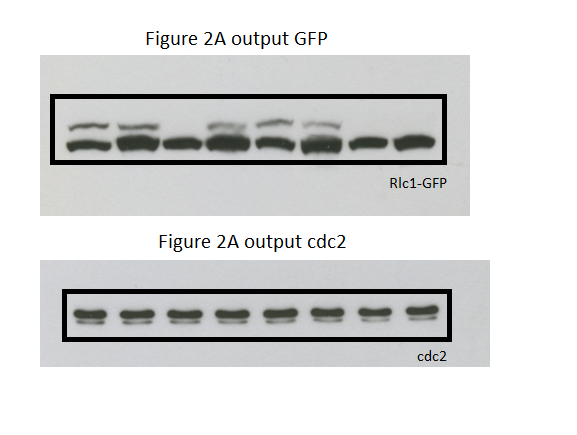

Supplement: Figure 2—source data 2. [file elife-83285-fig2-data2.zip › Figure 2A uncroped labeled gels.png]

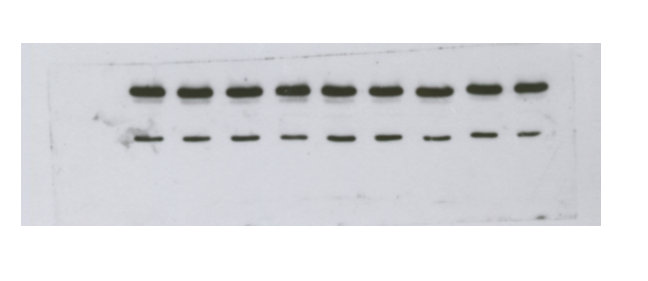

Supplement: Figure 2—source data 2. [file elife-83285-fig2-data2.zip › Figure 2C cdc2.png]

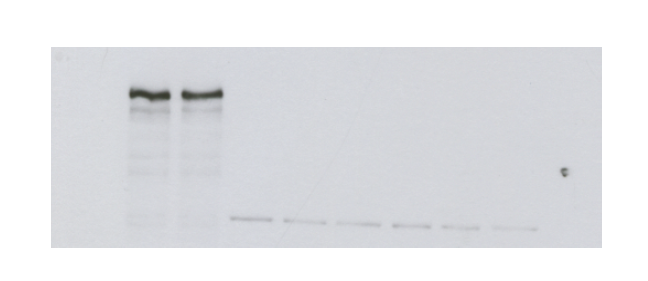

Supplement: Figure 2—source data 2. [file elife-83285-fig2-data2.zip › Figure 2C GFP.png]

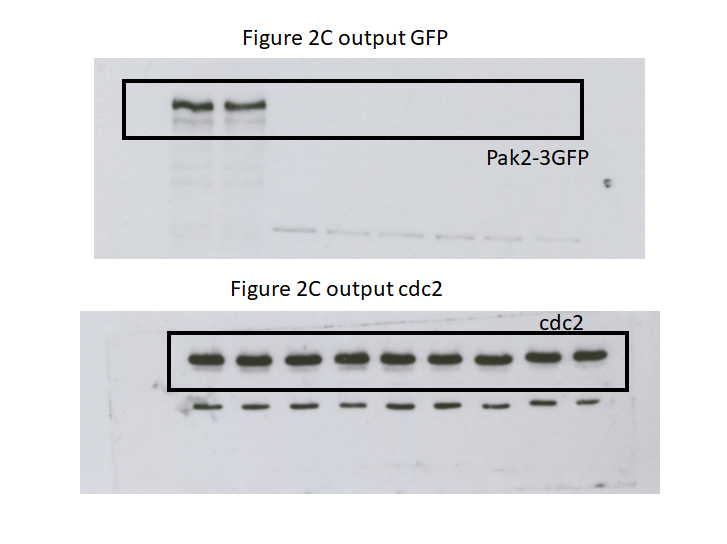

Supplement: Figure 2—source data 2. [file elife-83285-fig2-data2.zip › Figure 2C uncroped labeled gels.png]

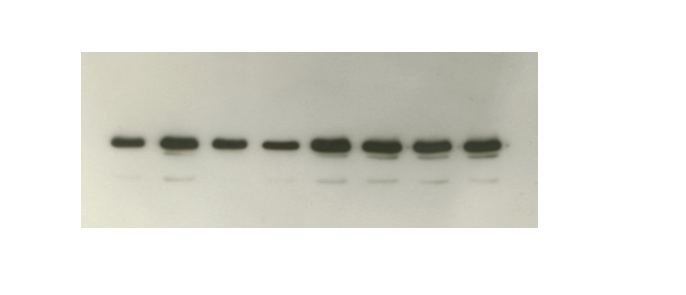

Supplement: Figure 2—source data 2. [file elife-83285-fig2-data2.zip › Figure 2D glycerol cdc2.png]

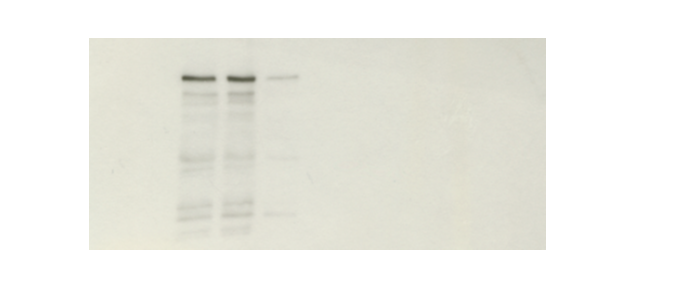

Supplement: Figure 2—source data 2. [file elife-83285-fig2-data2.zip › Figure 2D glycerol GFP.png]

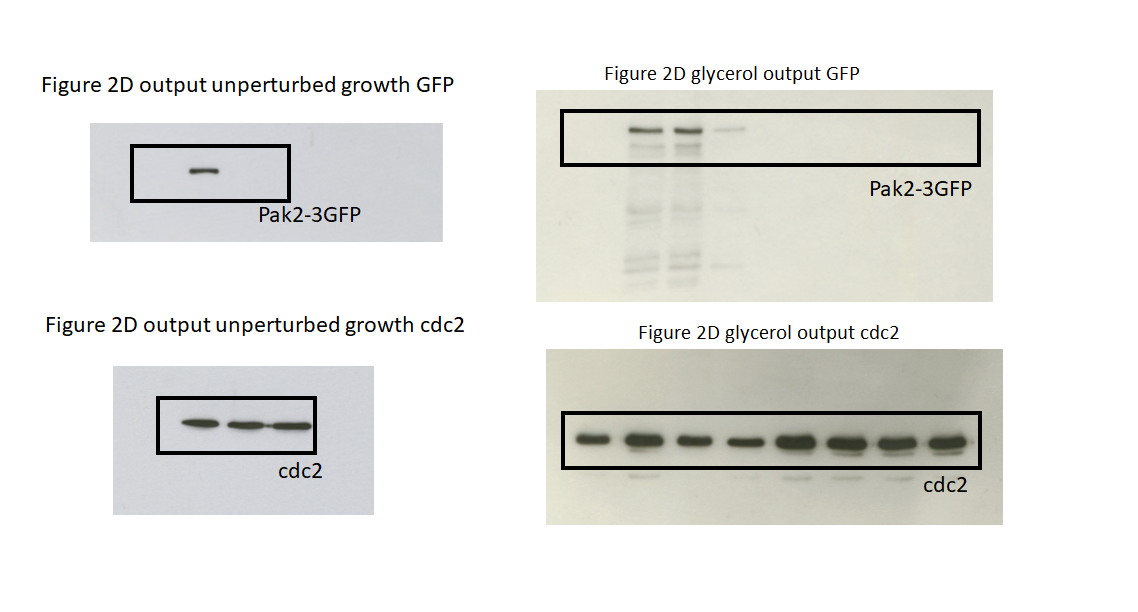

Supplement: Figure 2—source data 2. [file elife-83285-fig2-data2.zip › Figure 2D uncroped labeled gels.png]

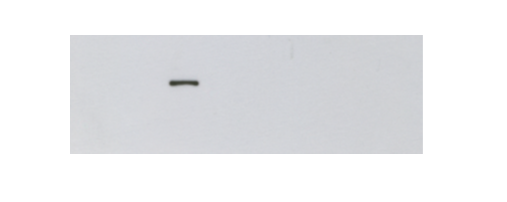

Supplement: Figure 2—source data 2. [file elife-83285-fig2-data2.zip › Figure 2D unperturbed growth GFP.png]

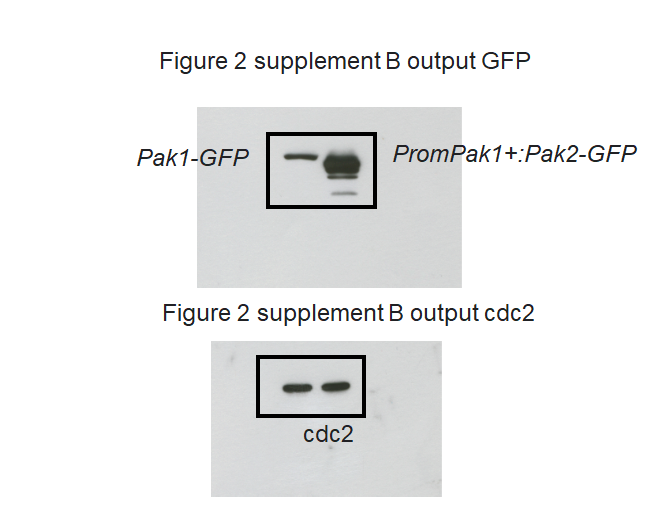

Supplement: Figure 2—figure supplement 1—source data 2. [file elife-83285-fig2-figsupp1-data2.zip › Figure 2 supplement D uncroped labeled gels.png]

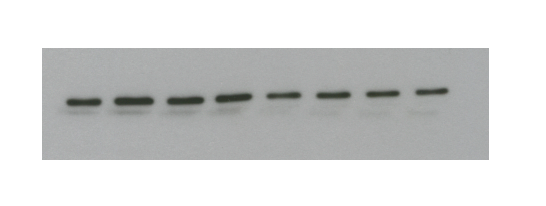

Supplement: Figure 2—figure supplement 1—source data 2. [file elife-83285-fig2-figsupp1-data2.zip › Figure 2 Supplement A cdc2.png]

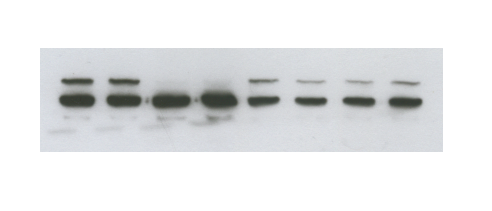

Supplement: Figure 2—figure supplement 1—source data 2. [file elife-83285-fig2-figsupp1-data2.zip › Figure 2 Supplement A GFP.png]

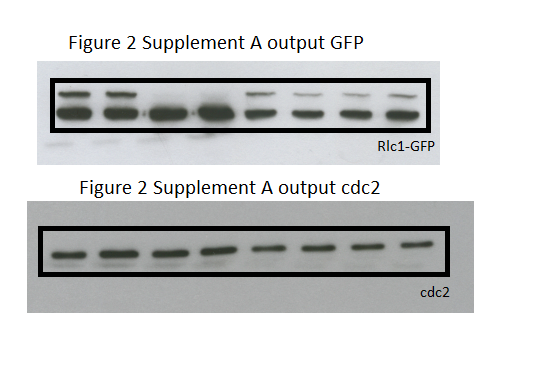

Supplement: Figure 2—figure supplement 1—source data 2. [file elife-83285-fig2-figsupp1-data2.zip › Figure 2 Supplement A uncroped labeled gels.png]

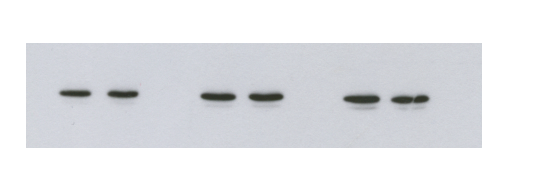

Supplement: Figure 2—figure supplement 1—source data 2. [file elife-83285-fig2-figsupp1-data2.zip › Figure 2 Supplement B cdc2.png]

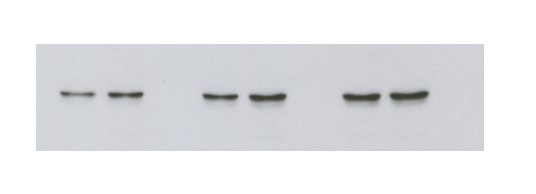

Supplement: Figure 2—figure supplement 1—source data 2. [file elife-83285-fig2-figsupp1-data2.zip › Figure 2 Supplement B GFP.png]

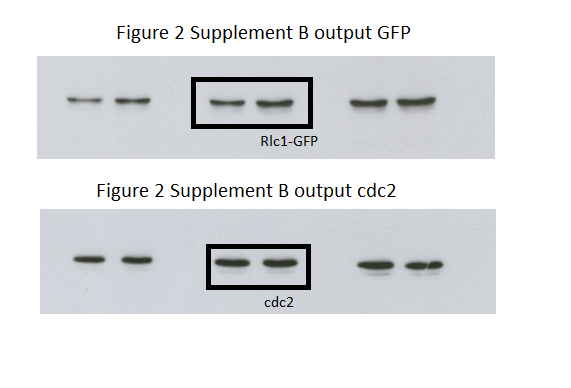

Supplement: Figure 2—figure supplement 1—source data 2. [file elife-83285-fig2-figsupp1-data2.zip › Figure 2 Supplement B uncroped labeled gels.png]

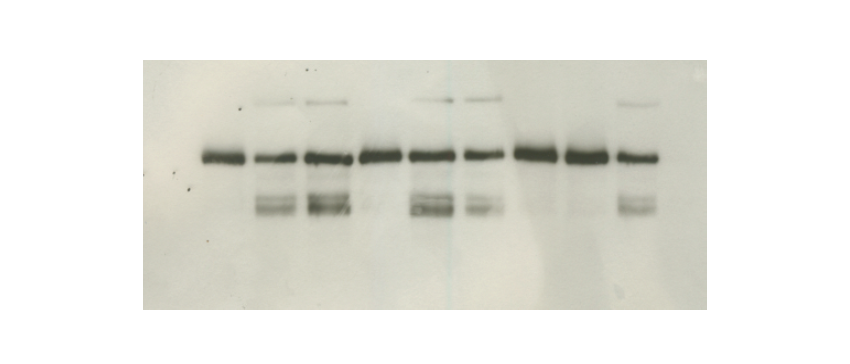

Supplement: Figure 2—figure supplement 1—source data 2. [file elife-83285-fig2-figsupp1-data2.zip › Figure 2 Supplement C GFP lower exposure.png]

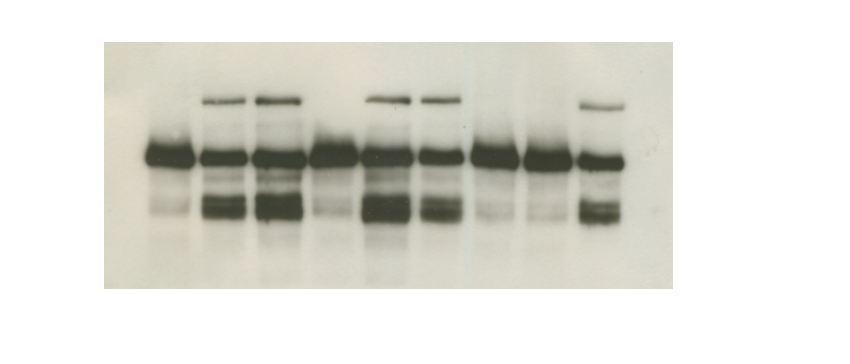

Supplement: Figure 2—figure supplement 1—source data 2. [file elife-83285-fig2-figsupp1-data2.zip › Figure 2 Supplement C GFP overexposure.png]

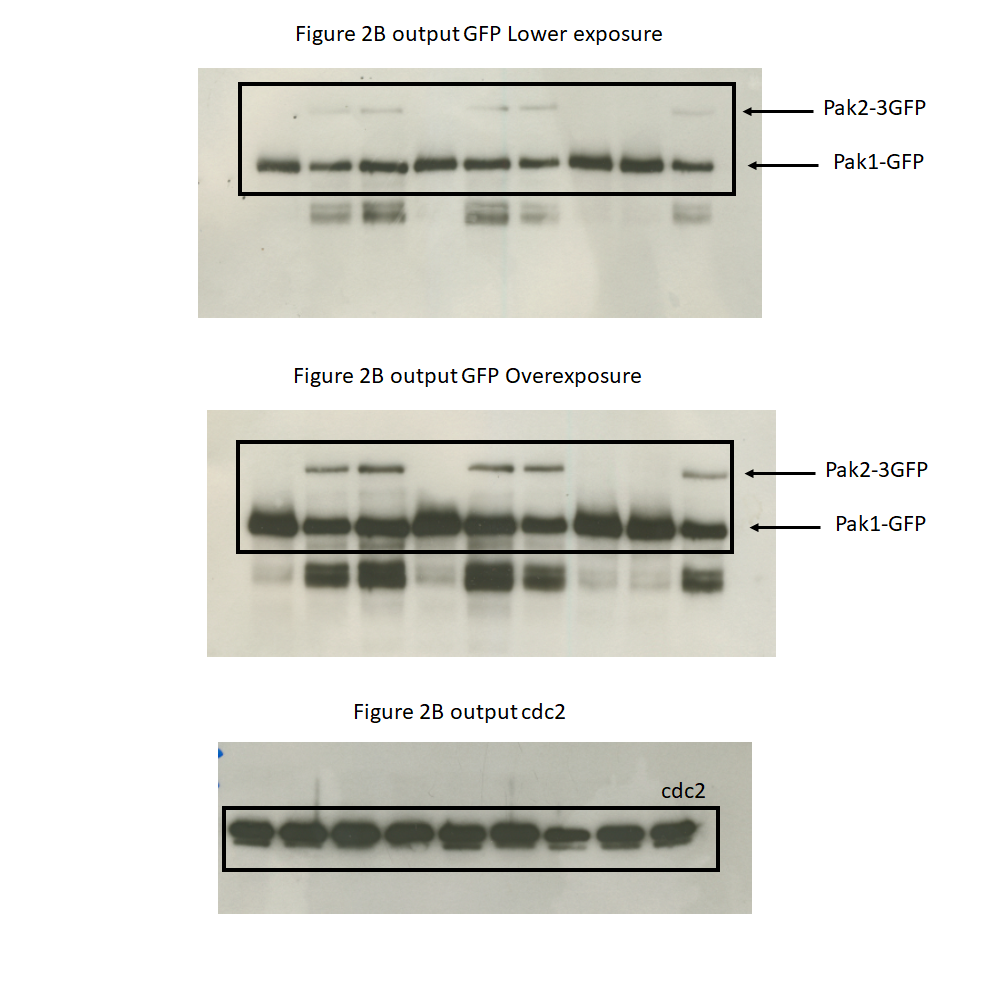

Supplement: Figure 2—figure supplement 1—source data 2. [file elife-83285-fig2-figsupp1-data2.zip › Figure 2 Supplement C uncroped labeled gels.png]

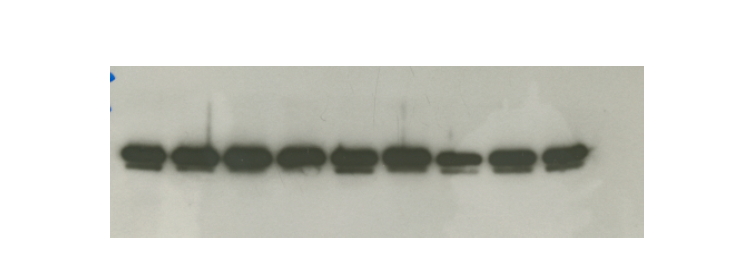

Supplement: Figure 2—figure supplement 1—source data 2. [file elife-83285-fig2-figsupp1-data2.zip › Figure 2 Supplement C cdc2.png]

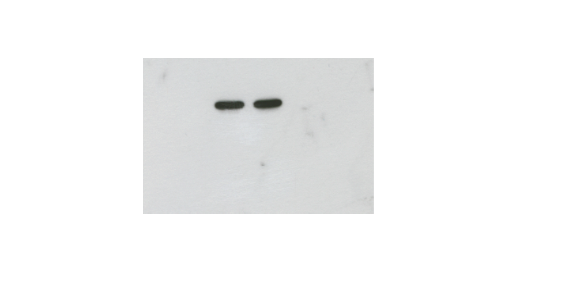

Supplement: Figure 2—figure supplement 1—source data 2. [file elife-83285-fig2-figsupp1-data2.zip › Figure 2 supplement D cdc2.png]

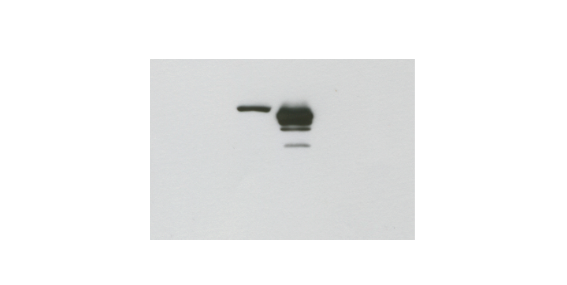

Supplement: Figure 2—figure supplement 1—source data 2. [file elife-83285-fig2-figsupp1-data2.zip › Figure 2 supplement D GFP.png]

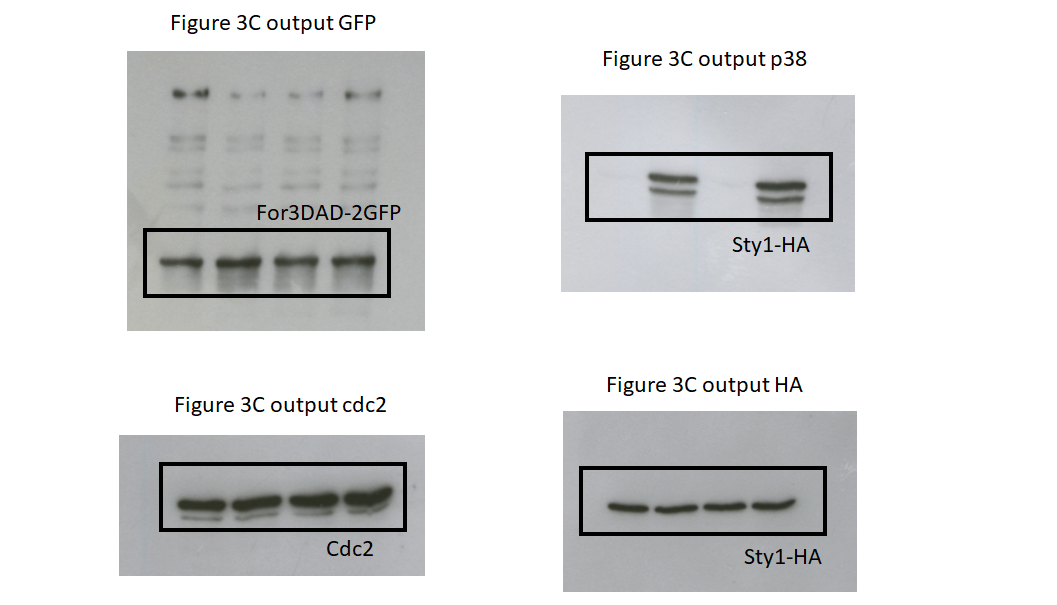

Supplement: Figure 3—source data 2. [file elife-83285-fig3-data2.zip › Figure 3C uncroped labeled gels.png]

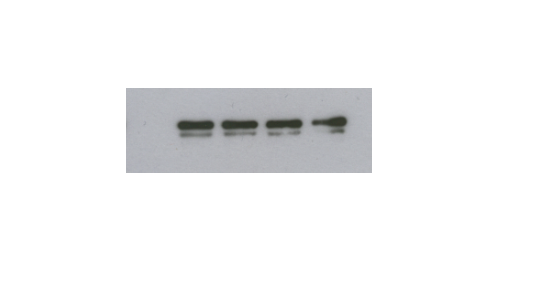

Supplement: Figure 3—source data 2. [file elife-83285-fig3-data2.zip › Figure 3A cdc2.png]

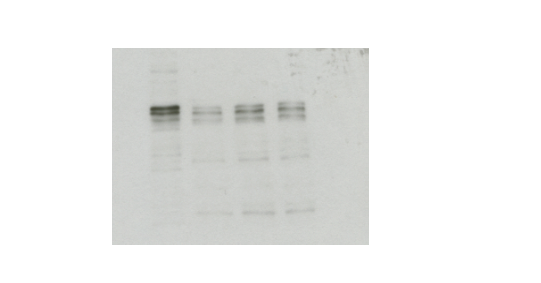

Supplement: Figure 3—source data 2. [file elife-83285-fig3-data2.zip › Figure 3A GFP.png]

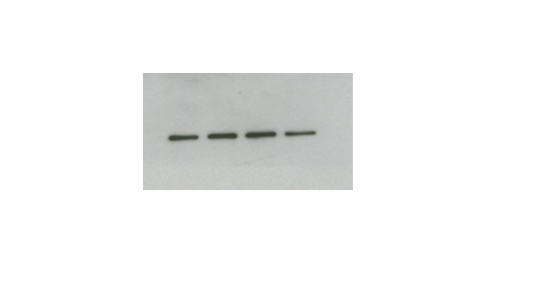

Supplement: Figure 3—source data 2. [file elife-83285-fig3-data2.zip › Figure 3A HA.png]

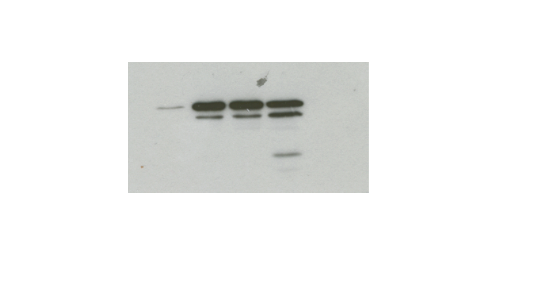

Supplement: Figure 3—source data 2. [file elife-83285-fig3-data2.zip › Figure 3A p38.png]

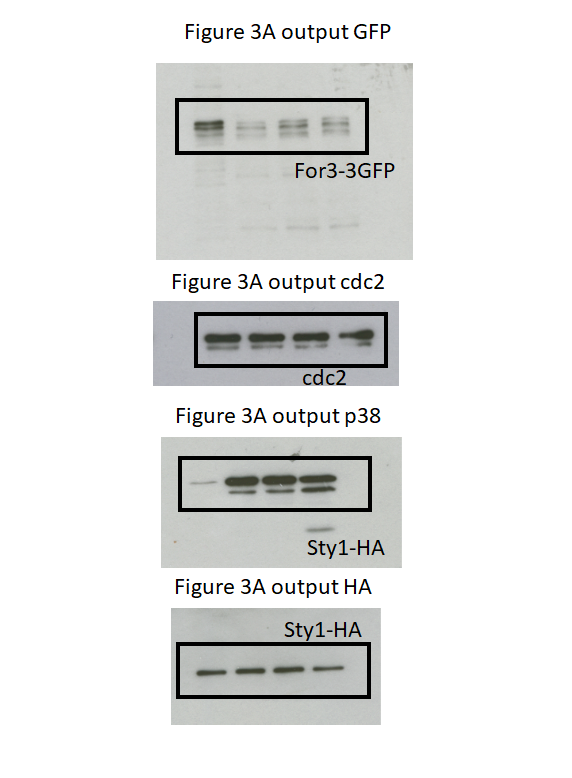

Supplement: Figure 3—source data 2. [file elife-83285-fig3-data2.zip › Figure 3A uncroped labeled gels.png]

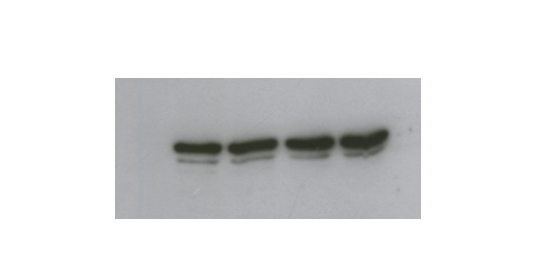

Supplement: Figure 3—source data 2. [file elife-83285-fig3-data2.zip › Figure 3C cdc2.png]

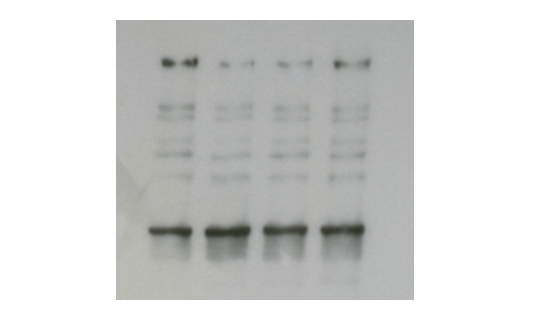

Supplement: Figure 3—source data 2. [file elife-83285-fig3-data2.zip › Figure 3C GFP.png]

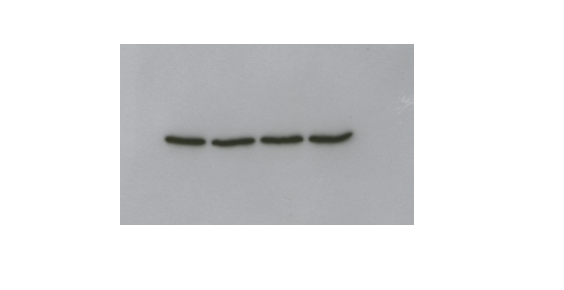

Supplement: Figure 3—source data 2. [file elife-83285-fig3-data2.zip › Figure 3C HA.png]

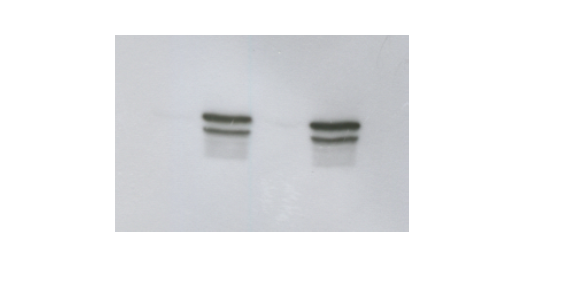

Supplement: Figure 3—source data 2. [file elife-83285-fig3-data2.zip › Figure 3C p38.png]

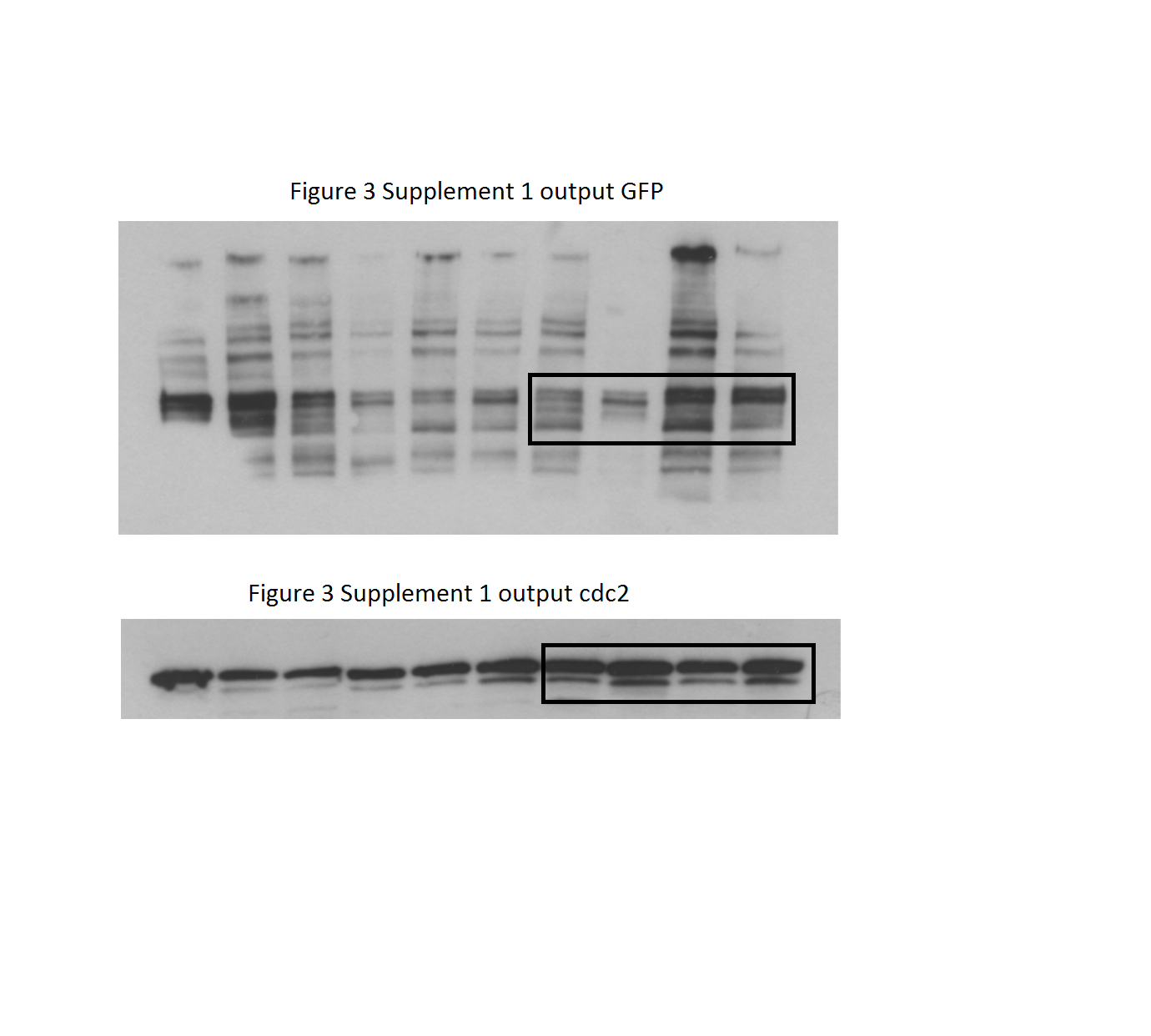

Supplement: Figure 3—figure supplement 1—source data 2. [file elife-83285-fig3-figsupp1-data2.zip › Figure 3 supplement F uncroped labeled gels.png]

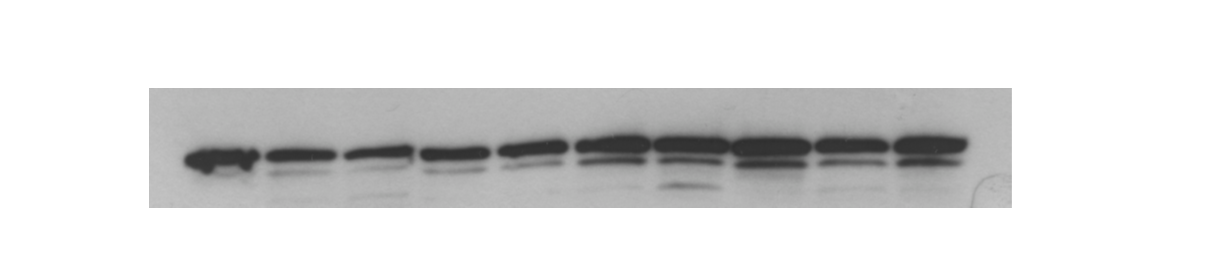

Supplement: Figure 3—figure supplement 1—source data 2. [file elife-83285-fig3-figsupp1-data2.zip › Figure 3 supplement F cdc2.png]

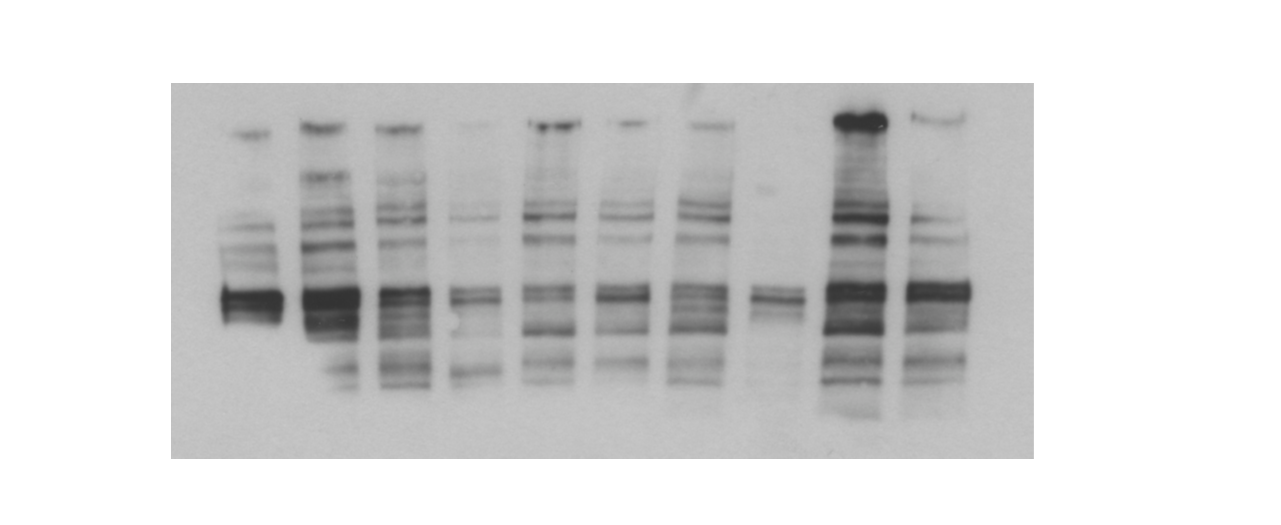

Supplement: Figure 3—figure supplement 1—source data 2. [file elife-83285-fig3-figsupp1-data2.zip › Figure 3 supplement F GFP.png]

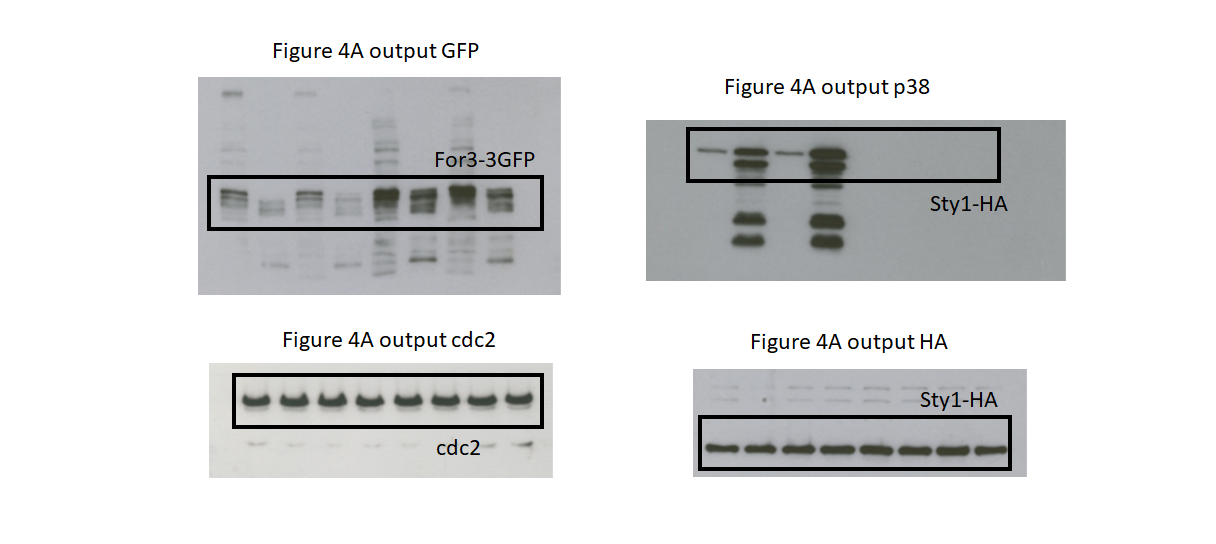

Supplement: Figure 4—source data 2. [file elife-83285-fig4-data2.zip › Figure 4A uncroped labeled gels.png]

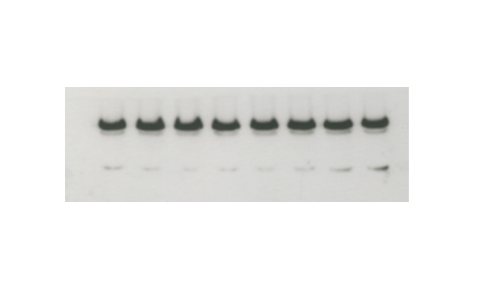

Supplement: Figure 4—source data 2. [file elife-83285-fig4-data2.zip › Figure 4A cdc2.png]

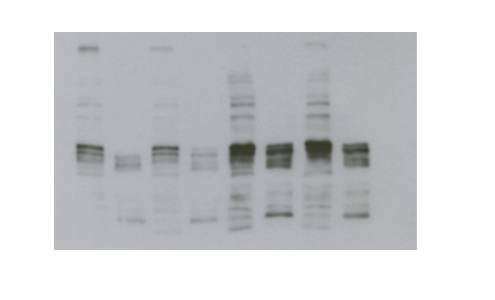

Supplement: Figure 4—source data 2. [file elife-83285-fig4-data2.zip › Figure 4A GFP.png]

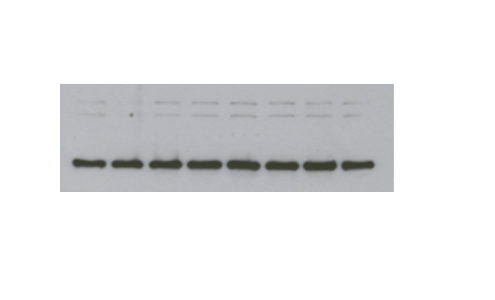

Supplement: Figure 4—source data 2. [file elife-83285-fig4-data2.zip › Figure 4A HA.png]

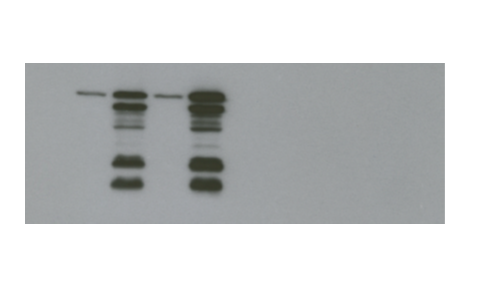

Supplement: Figure 4—source data 2. [file elife-83285-fig4-data2.zip › Figure 4A p38.png]

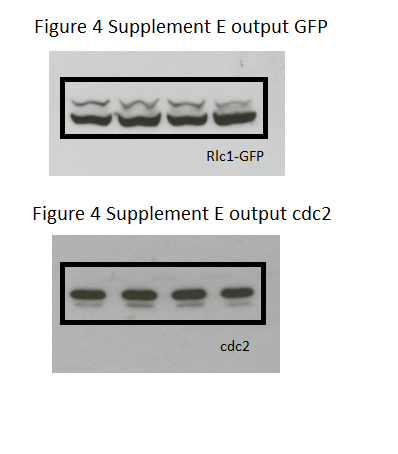

Supplement: Figure 4—figure supplement 1—source data 2. [file elife-83285-fig4-figsupp1-data2.zip › Figure 4 supplement E uncroped labeled gels.png]

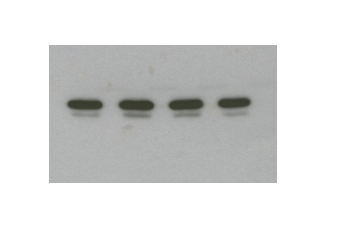

Supplement: Figure 4—figure supplement 1—source data 2. [file elife-83285-fig4-figsupp1-data2.zip › Figure 4 supplement E cdc2.png]

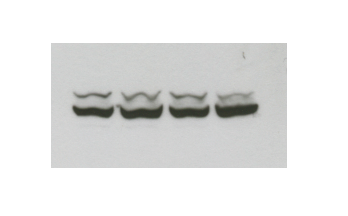

Supplement: Figure 4—figure supplement 1—source data 2. [file elife-83285-fig4-figsupp1-data2.zip › Figure 4 supplement E GFP.png]

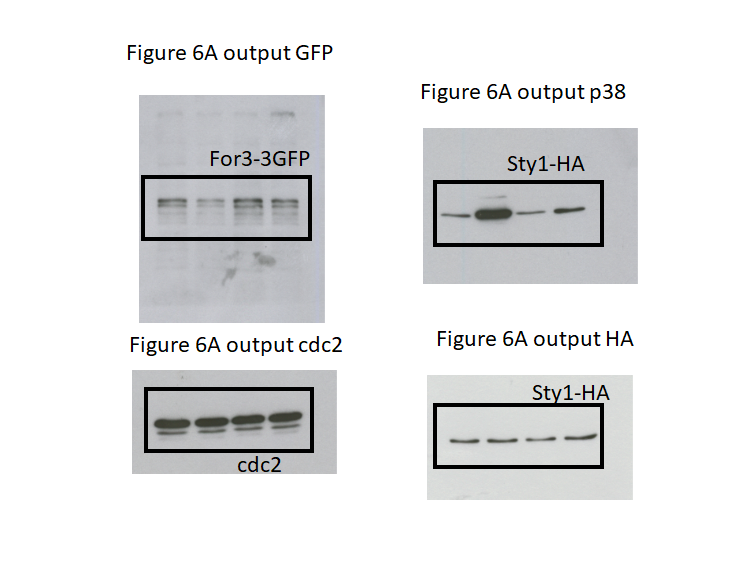

Supplement: Figure 6—source data 2. [file elife-83285-fig6-data2.zip › Figure 6A uncroped labeled gels.png]

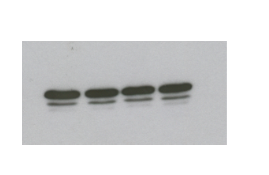

Supplement: Figure 6—source data 2. [file elife-83285-fig6-data2.zip › Figure 6A cdc2.png]

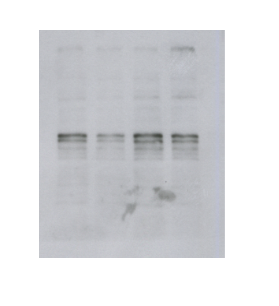

Supplement: Figure 6—source data 2. [file elife-83285-fig6-data2.zip › Figure 6A GFP.png]

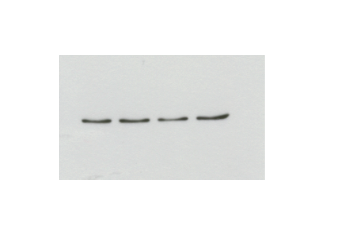

Supplement: Figure 6—source data 2. [file elife-83285-fig6-data2.zip › Figure 6A HA.png]

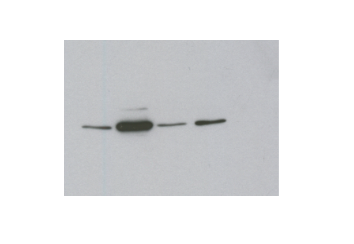

Supplement: Figure 6—source data 2. [file elife-83285-fig6-data2.zip › Figure 6A p38.png]
