# Supplementary material for: Myosin II regulatory light chain phosphorylation and formin availability modulate cytokinesis upon changes in carbohydrate metabolism
Source: eLife. 2023 Feb 24;12:e83285. doi: 10.7554/eLife.83285 (PMC10005788; doi:10.7554/eLife.83285)
Supplement: Supplementary file 1. [file elife-83285-supp1.docx]

**Supplementary File 1.** *S. pombe* strains used in this study.

| **Strain** | **Genotype** | **Source/Reference** |
| --- | --- | --- |
|  | **Figure 1** |  |
| FPR645 | *h^-^ rlc1::kanR ade6-M216 leu1-32 ura4.294* | This work |
| FPR961 | *h^+^ Pcp1-GFP:kanR rlc1::kanR Rlc1-GFP::ura4^+^ ade6-M216 leu1-32* | This work |
| FPR965 | *h^+^ Pcp1-GFP:kanR rlc1::kanR Rlc1(S35A)-GFP::ura4^+^ ade6-M216 leu1-32* | This work |
| FPR968 | *h^-^ Pcp1-GFP:kanR rlc1::kanR Rlc1(S36A)-GFP::ura4^+^ ade6-M216 leu1-32* | This work |
| FPR971 | *h^?^ Pcp1-GFP:kanR rlc1::kanR Rlc1(S35AS36A)-GFP::ura4^+^ ade6-M216 leu1-32* | This work |
| FPR1700 | *h^?^ Pcp1-GFP:kanR rlc1::kanR Rlc1(S35D)-GFP::ura4^+^ ade6-M216 leu1-32* | This work |
| FPR1698 | *h^?^ Pcp1-GFP:kanR rlc1::kanR Rlc1(S35DS36D)-GFP::ura4^+^ ade6-M216 leu1-32* | This work |
| FPR667 | *h^?^ Pcp1-GFP:kanR Rlc1-GFP:kanR cdc2ASM17:bsdR ade6-M216 leu1-32* | This work |
|  | **Figure 2** |  |
| MBY4489 | *h^-^ pak1-2xMyc-GFP::ura4^+^* | Loo *et al.,* 2008 |
| MBY5064 | *h^-^ pak1-M460G-2xMyc-GFP::ura4^+^* | Loo *et al.,* 2008 |
| FPR1621 | *h^?^ pak1-2xMyc-GFP::ura4^+^ rlc1::kanR Rlc1-GFP::ura4^+^ ade6-M216 leu1-32* | This work |
| FPR1635 | *h^?^ pak1M460G-2xMyc-GFP::ura4^+^ rlc1::kanR Rlc1-GFP::ura4^+^ade6-M216 leu1-32* | This work |
| FPR1641 | *h^?^ pak1-2xMyc-GFP::ura4^+^ rlc1::kanR shk2::kanR Rlc1-GFP::ura4^+^ ade6-M216 leu1-32* | This work |
| FPR1668 | *h^?^ pak1M460G-2xMyc-GFP::ura4^+^ rlc1::kanR shk2::kanR Rlc1-GFP::ura4^+^  ade6-M216 leu1-32* | This work |
| FPR1269 | *h^?^ pak1-2xMyc-GFP::ura4^+^ Pcp1-mcherry::ura4^+^ Rlc1-mcherry::kanR ade6-M216 leu1-32 ura4.D18* | This work |
| FPR1281 | *h^?^ pak1M460G-2xMyc-GFP::ura4^+^ Pcp1-mcherry::ura4^+^ Rlc1-mcherry::kanR ade6-M216 leu1-32 ura4.D18* | This work |
| FPR1272 | *h^?^ pak1-2xMyc-GFP::ura4^+^ Pcp1-mcherry::ura4^+^ Rlc1-mcherry::kanR shk2::kanR ade6-M216 leu1-32 ura4.D18* | This work |
| FPR1282 | *h^?^ pak1M460G-2xMyc-GFP::ura4^+^ Pcp1-mcherry::ura4^+^ Rlc1-mcherry::kanR shk2::kanR ade6-M216 leu1-32 ura4.D18* | This work |
| PPG4.69 | *h^+^ pak1-GFP::kanR leu1-32 ura4.D18* | Pilar Pérez |
| FPR1530 | *h^-^ shk2::hphR prompak1^+^shk2-GFP::ura4^+^ ade6-M216 leu1-32* | This work |
| FPR1460 | *h^-^ shk2::hphR shk2-3xGFP::ura4^+^ ade6-M216 leu1-32* | This work |
| FPR1554 | *h^-^ shk2::hphR shk2-3xGFP::ura4^+^ ste11::natR ade6-M216 leu1-32* | This work |
| FPR1481 | *h^-^ shk2::hphR promste11^+^A/G:shk2-3xGFP::ura4^+^ ade6-M216 leu1-32* | This work |
| FPR1607 | *h^?^ shk2::hphR shk2-3xGFP::ura4^+^ pka1::kanR ade6-M216 leu1-32* | This work |
| FPR1608 | *h^?^ shk2::hphR shk2-3xGFP::ura4^+^ rst2::natR ade6-M216 leu1-32* | This work |
| FPR1611 | *h^?^ shk2::hphR shk2-3xGFP::ura4^+^ pka1::kanR rst2::natR ade6-M216 leu1-32* | This work |
|  | **Figure 3** |  |
| E888 | *h^+^ for3-3xGFP::ura4^+^ sty1-HA6H::ura4^+^ ade6-M216 leu1-32 ura4-D18* | Gomez-Gil *et al.,* 2020 |
| FPR1441 | *h^?^ for3DAD-2xGFP::kanR sty1-HA6H::ura4^+^ rlc1::kanR rlc1-GFP::ura4^+^ ade6-M216 leu1-32 ura4-D18* | This work |
| FPR1443 | *h^?^ for3DAD-2xGFP::kanR sty1-HA6H::ura4^+^ rlc1::kanR rlc1(S35A)-GFP::ura4^+^ ade6-M216 leu1-32 ura4-D18* | This work |
| FPR961 | *h^+^ Pcp1-GFP:kanR rlc1::kanR Rlc1-GFP::ura4^+^ ade6-M216 leu1-32* | This work |
| FPR965 | *h^+^ Pcp1-GFP:kanR rlc1::kanR Rlc1(S35A)-GFP::ura4^+^ ade6-M216 leu1-32* | This work |
| FPR1292 | *h^?^ Pcp1-GFP:kanR rlc1::kanR Rlc1-GFP::ura4^+^ for3DAD ade6-M216 leu1-32* | This work |
| FPR1296 | *h^?^ Pcp1-GFP:kanR rlc1::kanR Rlc1(S35A)-GFP::ura4^+^ for3DAD ade6-M216 leu1-32* | This work |
| FPR1340 | *h^?^ Pcp1-mcherry::ura4+ Rlc1-mcherry::kanR for3-3xGFP::ura4^+^ pak1-M460G-2xMyc-GFP::ura4^+^ shk2::kanR ade6-M216 leu1-32 ura4.D18* | This work |
| FPR1345 | *h^?^ Pcp1-mcherry::ura4^+^ Rlc1-mcherry::kanR pak1-M460G-2xMyc-GFP::ura4^+^ for3DAD-2xGFP::kanR shk2::kanR ade6-M216 leu1-32 ura4.D18* | This work |
| FPR1089 | *h^?^ for3-3xGFP::ura4^+^ rlc1::kanR rlc1-HA::ura4+ kanR ade6-M216 leu1-32* | This work |
| FPR1094 | *h^?^ for3-3xGFP::ura4^+^ rlc1::kanR rlc1(S35A)-HA::ura4^+^ kanR ade6-M216 leu1-32* | This work |
| FPR1410 | *h^?^ for3DAD-2xGFP::kanR rlc1::kanR rlc1-HA::ura4^+^ kanR ade6-M216 leu1-32* | This work |
| FPR1415 | *h^?^ for3DAD-2xGFP::kanR rlc1::kanR rlc1(S35A)-HA::ura4^+^ kanR ade6-M216 leu1-32* | This work |
|  | **Figure 4** |  |
| FPR1000 | *h^+^ for3-3xGFP::ura4^+^ sty1-HA6H::ura4^+^ rlc1::kanR rlc1-GFP::ura4^+^ ade6-M216 leu1-32* | This work |
| FPR1048 | *h^+^ for3-3xGFP::ura4^+^ sty1-HA6H::ura4^+^ rlc1::kanR rlc1(S35A)-GFP::ura4^+^ ade6-M216 leu1-32* | This work |
| FPR1054 | *h^?^ for3-3xGFP::ura4^+^ sty1-HA6H::ura4^+^ rlc1::kanR rlc1-GFP::ura4^+^ wis1::his ade6-M216 leu1-32* | This work |
| FPR1059 | *h^?^ for3-3xGFP::ura4^+^ sty1-HA6H::ura4^+^ rlc1::kanR rlc1(S35A)-GFP::ura4^+^ wis1::his ade6-M216 leu1-32* | This work |
| FPR727 | *h^-^ rlc1::kanR rlc1-HA::ura4^+^ ade6-M216 leu1-32* | This work |
| FPR645 | *h^-^ rlc1::kanR ade6-M216 leu1-32 ura4.294* | This work |
| FPR730 | *h^-^ rlc1::kanR rlc1(S35A)-HA::ura4^+^ ade6-M216 leu1-32* | This work |
| FPR1228 | *h^-^ rlc1::kanR rlc1-HA::ura4^+^ wis1::his ade6-M216 leu1-32* | This work |
| FPR1231 | *h^-^ rlc1::kanR wis1::his ade6-M216 leu1-32* | This work |
| FPR1237 | *h^-^ rlc1::kanR rlc1(S35A)-HA::ura4^+^ wis1::his ade6-M216 leu1-32* | This work |
| FPR961 | *h^+^ Pcp1-GFP:kanR rlc1::kanR Rlc1-GFP::ura4^+^ ade6-M216 leu1-32* | This work |
| FPR965 | *h^+^ Pcp1-GFP:kanR rlc1::kanR Rlc1(S35A)-GFP::ura4^+^ ade6-M216 leu1-32* | This work |
| FPR1233 | *h^?^ Pcp1-GFP:kanR rlc1::kanR Rlc1-GFP::ura4^+^ wis1::his ade6-M216 leu1-32* | This work |
| FPR1235 | *h^?^ Pcp1-GFP:kanR rlc1::kanR Rlc1(S35A)-GFP::ura4^+^ wis1::his ade6-M216 leu1-32* | This work |
| FPR1361 | *h^?^ Pcp1-GFP:kanR rlc1::kanR Rlc1-GFP::ura4^+^ pyp1::kanR ade6-M216 leu1-32* | This work |
| FPR1406 | *h^?^ Pcp1-GFP:kanR rlc1::kanR Rlc1(S35A)-GFP::ura4^+^ pyp1::hphR ade6-M216 leu1-32* | This work |
|  | **Figure 5** |  |
| MM1 | *h^+^ ade6-M216 leu1-32 ura4.D18* | Madrid *et al.,* 2006 |
| FPR876 | *h- myo2.E1 ade6-M216 leu1-32 ura4.D18* | Balasubramanian *et al.,* 1998 |
| FPR462 | *h^+^ myo3::kanR ade6-M216 leu1-32 ura4.D18* | Lab Stock |
| FPR441 | *h^+^ cdc4-8 ade6-M216 leu1-32 ura4.D18* | Lab Stock |
| FPR645 | *h^-^ rlc1::kanR ade6-M216 leu1-32 ura4.294* | This work |
| FPR1461 | *h^?^ Pcp1-GFP:kanR rlc1::kanR Rlc1-GFP::ura4^+^ myo2.E1 ade6-M216 leu1-32* | This work |
| FPR1498 | *h^?^ Pcp1-GFP:kanR rlc1::kanR Rlc1-GFP::ura4^+^ myo2.E1 for3DAD ade6-M216 leu1-32* | This work |
|  | **Figure 6** |  |
| E888 | *h^+^ for3-3xGFP::ura4^+^ sty1-HA6H::ura4^+^ ade6-M216 leu1-32 ura4-D18* | Gomez-Gil *et al.,* 2020 |
| FPR961 | *h^+^ Pcp1-GFP:kanR rlc1::kanR Rlc1-GFP::ura4^+^ ade6-M216 leu1-32* | This work |
| FPR965 | *h^+^ Pcp1-GFP:kanR rlc1::kanR Rlc1(S35A)-GFP::ura4^+^ ade6-M216 leu1-32* | This work |
| FPR1461 | *h^?^ Pcp1-GFP:kanR rlc1::kanR Rlc1-GFP::ura4^+^ myo2.E1 ade6-M216 leu1-32* | This work |
| FPR1233 | *h^?^ Pcp1-GFP:kanR rlc1::kanR Rlc1-GFP::ura4^+^ wis1::his ade6-M216 leu1-32* | This work |
| FPR727 | *h^-^ rlc1::kanR rlc1-HA::ura4^+^ ade6-M216 leu1-32* | This work |
| FPR645 | *h^-^ rlc1::kanR ade6-M216 leu1-32 ura4.294* | This work |
| FPR730 | *h^-^ rlc1::kanR rlc1(S35A)-HA::ura4^+^ ade6-M216 leu1-32* | This work |
| FPR1489 | *h^?^ rlc1::kanR rlc1-HA::ura4^+^ myo2.E1 ade6-M216 leu1-32* | This work |
| FPR1142 | *h^?^ pak1M460G-2xMyc-GFP::ura4^+^ rlc1::kanR shk2::kanR rlc1-HA::ura4^+^  ade6-M216 leu1-32* | This work |
| FPR1228 | *h^-^ rlc1::kanR rlc1-HA::ura4^+^ wis1::his ade6-M216 leu1-32* | This work |
| FPR1130 | *h^?^ rlc1::kanR rlc1-HA::ura4^+^ for3::natR ade6-M216 leu1-32* | This work |
| FPR1135 | *h^?^ rlc1::kanR rlc1(S35A)-HA::ura4^+^ for3::natR ade6-M216 leu1-32* | This work |
|  | **Figure 1 supplement 1** |  |
| MM1 | *h^+^ ade6-M216 leu1-32 ura4.D18* | Madrid *et al.,* 2006 |
| FPR645 | *h^-^ rlc1::kanR ade6-M216 leu1-32 ura4.294* | This work |
| FPR961 | *h^+^ Pcp1-GFP:kanR rlc1::kanR Rlc1-GFP::ura4^+^ ade6-M216 leu1-32* | This work |
| FPR965 | *h^+^ Pcp1-GFP:kanR rlc1::kanR Rlc1(S35A)-GFP::ura4^+^ ade6-M216 leu1-32* | This work |
| FPR719 | *h^-^ rlc1::kanR pZ3EVrlc1-HA::ura4^+^ adh1-Z_3_EV:leu1^+^ ade6-M216* | This work |
| FPR722 | *h^-^ rlc1::kanR pZ3EVrlc1-HA::ura4^+^ adh1-Z_3_EV:leu1^+^ ade6-M216* | This work |
| FPR520 | *h^-^ rlc1:natR* | Pilar Pérez |
| FPR518 | *h^-^ rlc1::natR* | Pilar Pérez |
| FPR521 | *h^-^ rlc1(S35A):natR* | Pilar Pérez |
| FPR522 | *h^-^ rlc1(S35D):natR* | Pilar Pérez |
|  | **Figure 1 supplement 2** |  |
| FPR961 | *h^+^ Pcp1-GFP:kanR rlc1::kanR Rlc1-GFP::ura4^+^ ade6-M216 leu1-32* | This work |
| FPR965 | *h^+^ Pcp1-GFP:kanR rlc1::kanR Rlc1(S35A)-GFP::ura4^+^ ade6-M216 leu1-32* | This work |
| FPR1656 | *h^?^ Pcp1-GFP:kanR rlc1::kanR Rlc1-GFP::ura4^+^ myo51::ura4^+^ ade6-M216 leu1-32* | This work |
| FPR1671 | *h^?^ Pcp1-GFP:kanR rlc1::kanR Rlc1(S35A)-GFP::ura4^+^ myo51::ura4^+^ ade6-M216 leu1-32* | This work |
|  | **Figure 2 supplement 1** |  |
| FPR1680 | *h^?^ rlc1::kanR rlc1-GFP::ura4^+^ shk1::natR shk1(M460A)::hphR ade6-M216 leu1-32* | This work |
| FPR961 | *h^+^ Pcp1-GFP:kanR rlc1::kanR Rlc1-GFP::ura4^+^ ade6-M216 leu1-32* | This work |
| PPG4.69 | *h^+^ pak1-GFP::kanR leu1-32 ura4.D18* | Pilar Pérez |
| FPR1530 | *h^-^ shk2::hphR prompak1^+^shk2-GFP::ura4^+^ ade6-M216 leu1-32* | This work |
| FPR1482 | *h^?^ pak1-GFP::kanR shk2::hphR shk2-3xGFP::ura4^+^ ade6-M216 leu1-32* | This work |
| FPR1559 | *h^?^ shk2::hphR prompak1^+^shk2-GFP::ura4^+^ pcp1-mcherry::ura4^+^ Rlc1-mcherry::kanR ade6-M216 leu1-32* | This work |
| FPR1132 | *h^?^ pak1-2xMyc-GFP::ura4^+^ rlc1::kanR rlc1-HA::ura4^+^  ade6-M216 leu1-32* | This work |
| FPR1141 | *h^?^ pak1M460G-2xMyc-GFP::ura4^+^ rlc1::kanR rlc1-HA::ura4^+^  ade6-M216 leu1-32* | This work |
| FPR1145 | *h^?^ pak1-2xMyc-GFP::ura4^+^ rlc1::kanR shk2::kanR rlc1-HA::ura4^+^  ade6-M216 leu1-32* | This work |
| FPR1142 | *h^?^ pak1M460G-2xMyc-GFP::ura4^+^ rlc1::kanR shk2::kanR rlc1-HA::ura4^+^  ade6-M216 leu1-32* | This work |
|  | **Figure 3 supplement 1** |  |
| FPR961 | *h^+^ Pcp1-GFP:kanR rlc1::kanR Rlc1-GFP::ura4^+^ ade6-M216 leu1-32* | This work |
| FPR1126 | *h^?^ Pcp1-GFP:kanR rlc1::kanR Rlc1-GFP::ura4^+^ for3::natR ade6-M216 leu1-32* | This work |
| FPR727 | *h^-^ rlc1::kanR rlc1-HA::ura4^+^ ade6-M216 leu1-32* | This work |
| FPR1130 | *h^?^ rlc1::kanR rlc1-HA::ura4^+^ for3::natR ade6-M216 leu1-32* | This work |
| E880 | *h^+^ for3-3xGFP::ura4^+^ ade6-M216 leu1-32 ura4-D18* | Gomez-Gil *et al.,* 2020 |
| E998 | *h^?^ for3-3xGFP::ura4^+^ mts3-1 ade6-M216 leu1-32 ura4-D18* | Gomez-Gil *et al.,* 2020 |
|  | **Figure 3 supplement 2** |  |
| E32 | *h^-^ CRIB-GFP::ura4^+^ 1 ade6-M216 leu1-32* | Lab Stock |
| E888 | *h^+^ for3-3xGFP::ura4^+^ sty1-HA6H::ura4^+^ ade6-M216 leu1-32 ura4-D18* | Gomez-Gil *et al.,* 2020 |
| FPR961 | *h^+^ Pcp1-GFP:kanR rlc1::kanR Rlc1-GFP::ura4^+^ ade6-M216 leu1-32* | This work |
| FPR965 | *h^+^ Pcp1-GFP:kanR rlc1::kanR Rlc1(S35A)-GFP::ura4^+^ ade6-M216 leu1-32* | This work |
| FPR1292 | *h^?^ Pcp1-GFP:kanR rlc1::kanR Rlc1-GFP::ura4^+^ for3DAD ade6-M216 leu1-32* | This work |
| FPR1296 | *h^?^ Pcp1-GFP:kanR rlc1::kanR Rlc1(S35A)-GFP::ura4^+^ for3DAD ade6-M216 leu1-32* | This work |
| FPR1340 | *h^?^ Pcp1-mcherry::ura4+ Rlc1-mcherry::kanR for3-3xGFP::ura4^+^ pak1-M460G-2xMyc-GFP::ura4^+^ shk2::kanR ade6-M216 leu1-32 ura4.D18* | This work |
| FPR1345 | *h^?^ Pcp1-mcherry::ura4^+^ Rlc1-mcherry::kanR pak1-M460G-2xMyc-GFP::ura4^+^ for3DAD-2xGFP::kanR shk2::kanR ade6-M216 leu1-32 ura4.D18* | This work |
|  | **Figure 4 supplement 1** |  |
| FPR961 | *h^+^ Pcp1-GFP:kanR rlc1::kanR Rlc1-GFP::ura4^+^ ade6-M216 leu1-32* | This work |
| FPR965 | *h^+^ Pcp1-GFP:kanR rlc1::kanR Rlc1(S35A)-GFP::ura4^+^ ade6-M216 leu1-32* | This work |
| FPR1233 | *h^?^ Pcp1-GFP:kanR rlc1::kanR Rlc1-GFP::ura4^+^ wis1::his ade6-M216 leu1-32* | This work |
| FPR1235 | *h^?^ Pcp1-GFP:kanR rlc1::kanR Rlc1(S35A)-GFP::ura4^+^ wis1::his ade6-M216 leu1-32* | This work |
| MM1 | *h^+^ ade6-M216 leu1-32 ura4.D18* | Madrid *et al.,* 2006 |
| FPR17 | *h^-^ win1-1 sty1-HA6H:ura4^+^ ade6-M216 leu1-32 ura4.D18* | Lab stock |
| FPR29 | *h^+^ wak1::ura4^+^ sty1-HA6H:ura4^+^ ade6-M216 leu1-32 ura4.D18* | Lab stock |
| FPR36 | *h^-^ wis1::his sty1-HA6H:ura4^+^ ade6-M216 leu1-32 ura4.D18* | Lab stock |
| FPR152 | *h^-^ sty1::ura4^+^ ade6-M216 leu1-32 ura4.D18* | Lab stock |
| FPR232 | *h^-^ atf1::ura4^+^ sty1-HA6H:ura4^+^ ade6-M216 leu1-32 ura4.D18* | Lab stock |
| FPR1000 | *h^+^ for3-3xGFP::ura4^+^ sty1-HA6H::ura4^+^ rlc1::kanR rlc1-GFP::ura4^+^ ade6-M216 leu1-32* | This work |
| FPR1048 | *h^+^ for3-3xGFP::ura4^+^ sty1-HA6H::ura4^+^ rlc1::kanR rlc1(S35A)-GFP::ura4^+^ ade6-M216 leu1-32* | This work |
| FPR1441 | *h^?^ for3DAD-2xGFP::kanR sty1-HA6H::ura4^+^ rlc1::kanR rlc1-GFP::ura4^+^ ade6-M216 leu1-32 ura4-D18* | This work |
| FPR1443 | *h^?^ for3DAD-2xGFP::kanR sty1-HA6H::ura4^+^ rlc1::kanR rlc1(S35A)-GFP::ura4^+^ ade6-M216 leu1-32 ura4-D18* | This work |
| FPR1361 | *h^?^ Pcp1-GFP:kanR rlc1::kanR Rlc1-GFP::ura4^+^ pyp1::kanR ade6-M216 leu1-32* | This work |
| FPR1406 | *h^?^ Pcp1-GFP:kanR rlc1::kanR Rlc1(S35A)-GFP::ura4^+^ pyp1::hphR ade6-M216 leu1-32* | This work |
|  | **Figure 5 supplement 1** |  |
| FPR1461 | *h^?^ Pcp1-GFP:kanR rlc1::kanR Rlc1-GFP::ura4^+^ myo2.E1 ade6-M216 leu1-32* | This work |
| FPR1498 | *h^?^ Pcp1-GFP:kanR rlc1::kanR Rlc1-GFP::ura4^+^ myo2.E1 for3DAD ade6-M216 leu1-32* | This work |
|  | **Figure 5 supplement 2** |  |
| FPR961 | *h^+^ Pcp1-GFP:kanR rlc1::kanR Rlc1-GFP::ura4^+^ ade6-M216 leu1-32* | This work |
| FPR1547 | *h^-^ Pcp1-GFP:kanR rlc1::kanR Rlc1-GFP::ura4^+^ myo3::kanR ade6-M216 leu1-32* | This work |
| FPR965 | *h^+^ Pcp1-GFP:kanR rlc1::kanR Rlc1(S35A)-GFP::ura4^+^ ade6-M216 leu1-32* | This work |
| FPR1619 | *h^?^ Pcp1-GFP:kanR rlc1::kanR Rlc1(S35A)-GFP::ura4^+^ myo3::kanR ade6-M216 leu1-32* | This work |
|  | **Figure 6 supplement 1** |  |
| FPR961 | *h^+^ Pcp1-GFP:kanR rlc1::kanR Rlc1-GFP::ura4^+^ ade6-M216 leu1-32* | This work |
| FPR965 | *h^+^ Pcp1-GFP:kanR rlc1::kanR Rlc1(S35A)-GFP::ura4^+^ ade6-M216 leu1-32* | This work |
| FPR1461 | *h^?^ Pcp1-GFP:kanR rlc1::kanR Rlc1-GFP::ura4^+^ myo2.E1 ade6-M216 leu1-32* | This work |
