# Supplementary material for: Myosin II regulatory light chain phosphorylation and formin availability modulate cytokinesis upon changes in carbohydrate metabolism
Source: eLife. 2023 Feb 24;12:e83285. doi: 10.7554/eLife.83285 (PMC10005788; doi:10.7554/eLife.83285)
Supplement: Supplementary file 2. [file elife-83285-supp2.docx]

**Supplementary File 2.** Oligonucleotides and DNA fragments used in this study.

| **OLIGONUCLEOTIDE** | **SEQUENCE 5’-3’** | **Use** |
| --- | --- | --- |
| Rlc1D-FWD | AGTTTTTTTCATTTCTTAATTCTTCCGTACTTTACTTTACAGCATAACTATATCTTATTTGATCATTTGCTCTGTTAACG CGGATCCCCGGGTTAATTAA | *rlc1^+^* deletion |
| Rlc1D-REV | TTCGTCTAAGGGAAATGGCTCAGGTTAAAAAGATAAAGTATTAGAGGGAAGAATGTGAAACATATCTGGCTGCTCTTAACGAATTCGAGCTCGTTTAAAC | *rlc1^+^* deletion |
| Rlc1D-COMP FWD | ATCCTCGCCTTACGGTGTATAA | Confirmation of *rlc1^+^* deletion |
| KAN-COMP-R | GATGTGAGAACTGTATCCTAGCAAG | Common oligonucleotide for confirmation of gene tagging |
| Rlc1-Seq1-FWD | CTGAGACTTACCAAGAGCTTGAATATC | *rlc1^+^* sequencing |
| Rlc1-S35A-FWD | **T TCT CAA AGA GTT GCT GCC CAA GCC GCT AAA CGA GCA GCT TCT GGT GCA TTT GCG CAA CTT ACT TCT TCC CAA ATT CAA G** | Rlc1 serine-35 replaced by alanine (site-directed mutagenesis) |
| Rlc1-S35A-REV | CTTGAATTTGGGAAGAAGTAAGTTGCGCAAATGCACCAGAAGCTGCTCGTTTAGCGGCTTGGGCAGCAACTCTTTGAGAA | Rlc1 serine-35 replaced by alanine (site-directed mutagenesis) |
| Rlc1-S36A-FWD | **T TCT CAA AGA GTT GCT GCC CAA GCC GCT AAA CGA GCA TCT GCT GGT GCA TTT GCG CAA CTT ACT TCT TCC CAA ATT CAA G** | Rlc1 serine-36 replaced by alanine (site-directed mutagenesis) |
| Rlc1-S36A-REV | CTTGAATTTGGGAAGAAGTAAGTTGCGCAAATGCACCAGCAGATGCTCGTTTAGCGGCTTGGGCAGCAACTCTTTGAGAA | Rlc1 serine-36 replaced by alanine (site-directed mutagenesis) |
| Rlc1-S35AS36A-FWD | **T TCT CAA AGA GTT GCT GCC CAA GCC GCT AAA CGA GCA GCT GCT GGT GCA TTT GCG CAA CTT ACT TCT TCC CAA ATT CAA G** | Rlc1 serine-35 and serine-36 replaced by alanine (site-directed mutagenesis) |
| Rlc1-S35AS36A-REV | CTTGAATTTGGGAAGAAGTAAGTTGCGCAAATGCACCAGCAGCTGCTCGTTTAGCGGCTTGGGCAGCAACTCTTTGAGAA | Rlc1 serine-35 and serine-36 replaced by alanine (site-directed mutagenesis) |
| Rlc1-S35D-FWD | **T TCT CAA AGA GTT GCT GCC CAA GCC GCT AAA CGA GCA GAT TCT GGT GCA TTT GCG CAA CTT ACT TCT TCC CAA ATT CAA G** | Rlc1 serine-35 replaced by aspartic acid (site-directed mutagenesis) |
| Rlc1-S35D-REV | CTTGAATTTGGGAAGAAGTAAGTTGCGCAAATGCACCAGAATCTGCTCGTTTAGCGGCTTGGGCAGCAACTCTTTGAGAA | Rlc1 serine-35 replaced by aspartic acid (site-directed mutagenesis) |
| Rlc1-S35DS36D-FWD | **T TCT CAA AGA GTT GCT GCC CAA GCC GCT AAA CGA GCA GAT GAT GGT GCA TTT GCG CAA CTT ACT TCT TCC CAA ATT CAA G** | Rlc1 serine-35 and serine-36 replaced by aspartic acid (site-directed mutagenesis) |
| Rlc1-S35DS36D-REV | CTTGAATTTGGGAAGAAGTAAGTTGCGCAAATGCACCATCATCTGCTCGTTTAGCGGCTTGGGCAGCAACTCTTTGAGAA | Rlc1 serine-35 and serine-36 replaced by aspartic acid (site-directed mutagenesis) |
| Rlc1 (SmaI)- FWD | TAT ATC CCG GGA TGT TCT CTT CGA AGG AAA ATT CCT | *rlc1^+^* clonning in pZ3Ev plasmid |
| Rlc1-HA (SacII)-REV | TAT ATC CGC GGT CAT GCA TAG TCC GGG ACG TCA TAG GGA TAG CCA TTG CTA TCT TTT GAC CCA GCA | *rlc1^+^* clonning in pZ3Ev plasmid |
| PromRlc1(XhoI)-FWD | **TATAACTCGAG**GGTGTGCAAGTTCAGACTC | *rlc1^+^* clonning in Pjk210 plasmid |
| Rlc1-GFP(SacII)-REV | TATTACCGCGGCAGATCTATATTACCCTG | *rlc1^+^* clonning in Pjk210 plasmid |
| Pak2-Comp-FWD | TGTAACCAATGTCATGTTCGCT | Confirmation of *pak2^+^* deletion |
| **PromPak2(XhoI)-FWD** | ACTTACTCGAGCAGTACTCCCAACTTGTTAGATAATG | *Pak2^+^* clonning in Pjk210 plasmid |
| **Pak2GFP(SmaI/XmaI)-REV** | ATTAACCCGGGATT AAT ATG GGT ATT CGC TTT GC | *Pak2^+^* clonning in Pjk210 plasmid |
| **GFP1-FWD** | tctcgcaaagcgaatacccatattaatccc AGTAAAGGAGAAGAACTTTTCACTGG | *GFP-tagging in Pak2-3GFP assembly* |
| **GFP1-REV** | tcctttactggatctTTTGTATAGTTCATCCATGCCATGTG | *GFP-tagging in Pak2-3GFP assembly* |
| **GFP2-FWD** | gatgaactatacaaaAGATCCAGTAAAGGAGAAGAACTTTTC | *GFP-tagging in Pak2-3GFP assembly* |
| **GFP2-REV** | aagttcttctcctttactgttaattaacccTTTGTATAGTTCATCCATGCCATGTG | *GFP-tagging in Pak2-3GFP assembly* |
| **PromPak1-FWD** | cgacggtatcgataagcttgatatcgaattcctgcagccc TTTAAAAGTATTTGAGTATAATAAATGAAAATTAG | *Pak2-GFP Pak1^+^ promoter assembly* |
| **PromPak1-REV** | cctcttacacttaaaagcatAGTAAATAAATTTATTAACGAAAAGGG | *Pak2-GFP Pak1^+^ promoter assembly* |
| **Pak2-GFP-FWD** | cgttaataaatttatttactATGCTTTTAAGTGTAAGAGGCGTGC | *Pak2-GFP Pak1^+^ promoter assembly* |
| **Pak2-GFP-REV** | caagggagacattccttttaCTATTTGTATAGTTCATCCATGCCATG | *Pak2-GFP Pak1^+^ promoter assembly* |
| **Nmt-TERM-FWD** | tggatgaactatacaaatagTAAAAGGAATGTCTCCCTTGCCAGTAC | *Pak2-GFP Pak1^+^ promoter assembly Pak2-GFP Pak1^+^ promoter assembly* |
| **Nmt1-TERM-REV** | ccaccgcggtggcggccgctctagaactagtggatccccc GCATTACTAATAGAAAGGATTATTTCACTTCTAATTACAC | *Pak2-GFP Pak1^+^ promoter assembly* |
| **Ste11-del-FWD** | TTAATTCTAAGAACCGTTTCATTTGTTTTATTTTCTCCCTTACTTTTACTACAATTTTTATATTTACTCTTCTCTACACACGGATCCCCGGGTTAATTAA | *ste11^+^* deletion |
| **Ste11-del-REV** | ACAAATCAGCTGCATGCTTTTGTGACGCGTTAAAAATGATCGTTTGTTGAAAACAAAGCCATGTTTGCATAGAAATATTTGAATTCGAGCTCGTTTAAAC | *ste11^+^* deletion |
| **Ste11-comp-FWD** | TCACACCAGTTTTTATTCGGTG | Confirmation of *ste11^+^* deletion |
| **PromSte11-FWD** | CATAACATTTCTTTGTTTTT  TGCATTCTCTTTATTTATAAATGGGGTTTCTTTATTTATACATTTGAGCTAAAATGTATA | *ste11^+^* promoter G replaced by A (site directed mutagenesis) |
| **PromSte11-REV** | TATACATTTTAGCTCAAATGTATAAATAAAGAAACCCCATTTATAAATAAAGAGAATGCAAAAAACAAAGAAATGTTATG | *ste11^+^* promoter G replaced by A (site directed mutagenesis) |
| **For3-Seq-F** | GGCCACACTGAACCAAAGAG | *for3^+^* sequencing |
| **Myp2-Comp-FWD** | GTGCCTAATATCCTGGCAAAAG | Confirmation of *myo3^+^* deletion |
| **For3-Comp-FWD** | CATCACATCTGATACCTGCGTT | Confirmation of *for3^+^* deletion |
| **Myo51-Comp-FWD** | TCGAAACTCAAGTTACCCGATT | Confirmation of *myo51^+^* deletion |
| **Nat-Comp-REV** | TTATTGTCAGTACTGATTAGGGGCA | Common oligonucleotide for confirmation of gene tagging |
| Ura-Comp-REV | CAAGAGACCACGTCCCAAAGG | Common oligonucleotide for confirmation of gene tagging |
